# Circulating SARS-CoV-2 spike IgG antibody responses in cancer patients following multiple COVID-19 vaccination boosters

**DOI:** 10.3389/fimmu.2025.1629473

**Published:** 2025-08-12

**Authors:** Huijing Xue, Troy J. Kemp, Hayley North, Nancy V. Roche, Thomas E. Hickey, Ligia A. Pinto

**Affiliations:** Vaccine, Immunity and Cancer Directorate, Frederick National Laboratory for Cancer Research, Frederick, MD, United States

**Keywords:** COVID-19, serology, avidity, mRNA vaccine, SARS-CoV-2

## Abstract

**Introduction:**

Individuals with cancer have a higher risk of SARS-CoV-2 infection, severe disease, hospitalization and death compared to healthy individuals. Understanding the immune response to different doses of COVID-19 vaccines in this population is essential to inform vaccine recommendations. This study aimed to compare the post-vaccination humoral immune response of people with cancer versus healthy participants via assessment of anti-spike IgG antibody levels and avidity 1 month and 6 months post-last vaccination.

**Methods:**

Circulating anti-spike IgG levels and antibody avidity were measured in sera from cancer and healthy cohorts using ELISA and chaotropic-based avidity assays.

**Results:**

In general, individuals with hematological cancers showed significantly lower antibody levels and avidity across two-, three- and four-doses compared to healthy individuals. Additionally, individuals with hematological cancers who received two doses of vaccine exhibited a significantly slower avidity development at both time points compared to healthy individuals. In contrast, individuals with solid cancers exhibited similar antibody levels and avidity compared to healthy participants. Factors including age, sex and vaccine received also influenced immune responses.

**Discussion:**

These findings suggest the need for customized vaccination strategies for vulnerable populations.

## 1 Introduction

Coronavirus disease 2019 (COVID-19), caused by infection with severe acute respiratory syndrome coronavirus 2 (SARS-CoV-2), has spread rapidly since December 2019. Studies have shown that individuals diagnosed with cancer face increased risks of developing severe COVID-19 disease and COVID-associated death compared to healthy populations (1–4). Differences in COVID-19 adverse outcomes and survival rates across various cancer subtypes further highlight the need for customized prevention, care, and treatment for individuals with cancer (5–7).

Roughly a year into the pandemic, emergency use authorization (EUA) was granted to two novel messenger ribonucleic acid (mRNA) SARS-CoV-2 vaccines based on studies demonstrating their safety and efficacy in healthy volunteers (8–12). These new vaccines were particularly novel given their use of mRNA-vaccine technology, as historically most approved vaccines have been protein-based. Once the SARS-CoV-2 vaccines became available, experts advised that individuals with cancer be given priority for COVID-19 vaccination, but no other specific guidance was distributed until later, when individuals with immunocompromising conditions were recommended to receive additional doses (10, 13). Multiple variants of concern (VOC) have emerged as the pandemic progressed, evading the protection provided by the original vaccines (14–16). In response to this, updated formulations have been produced and authorized, including a bivalent booster to target the original strain and the highly transmissible omicron variants (EUA approved late 2022), an updated monovalent vaccine targeting XBB.1.5 omicron variant of BA.2 (EUA approved September 2023), and an updated monovalent vaccine targeting the KP.2 variant of the omicron JN.1 lineage (EUA approved August 2024) (17–20). The updated formulations have provided additional protection as the virus evolved. However, a better understanding of the immunogenicity of the updated formulations in both healthy and vulnerable populations (such as patients with cancer) is still needed.

Various studies have demonstrated that vaccine-induced circulating antibody levels and vaccine efficacy wane within the first 3-6 months of vaccination in both healthy individuals and people diagnosed with cancer, resulting in a need for booster doses regardless of the viral variant in circulation (21–23). In addition, individuals with cancer have been identified as developing poor responses to initial primary vaccination (two doses), but additional doses will usually improve their serum anti-spike IgG levels and infection rates (24–27). As of October 31, 2024, the CDC has recommended that individuals with a weakened immune system stay up to date with the latest vaccination recommendations (currently three doses of the 2024-2025 formulation for individuals with immunocompromising conditions if they are previously unvaccinated, one dose if previously fully vaccinated with three doses of older formulations) and as well as receive an additional dose of the 2024-2025 formulation 6 months after the last dose (28). However, individuals with cancer were not included in the initial safety and efficacy clinical trials of the original formulations, leading to significant gaps in our understanding of the new vaccine platforms’ effectiveness, duration of imparted immunity, and safety of repeated COVID-19 booster doses in this vulnerable population (11, 12). Furthermore, significant differences in vaccine responses have been reported depending on cancer types, treatments received, and vaccine regimens (29–32). Thus, evaluating the immune response to COVID-19 vaccination in individuals with cancer is essential to better inform vaccination schedules and recommendations in these populations.

While no correlate of protection (CoP) or minimum level of antibody has been established to determine vaccine effectiveness, neutralizing antibody titers and binding antibody levels are well recognized as immunological markers that correlate with protection against severe disease (33–35). Anti-spike IgG levels significantly correlate with neutralizing activity against highly related virus types, highlighting the relevance of measuring specific antibody levels to evaluate vaccine-induced immunity (36). In addition, the quality or avidity (strength of binding) of these antibodies is an important factor influencing the overall immune response, as higher avidity is generally associated with more effective neutralization and protection (37, 38). This study aims to evaluate and compare the level and the quality (avidity) of antibody responses to different numbers of doses of SARS-CoV-2 mRNA-based vaccines in individuals with cancer compared to healthy individuals. The influence of age, sex, and vaccine formulation on the level and quality (avidity) of circulating antibodies were also evaluated.

## 2 Results

### 2.1 Demographic characteristics of the study participants

The levels of anti-SARS-CoV-2 IgG antibodies were investigated in the sera of healthy participants (n=352) and individuals with cancer (N=221). Individuals with cancer were further grouped into hematological cancer (N=79) and solid cancer (N=142) cohorts. The cancer cohorts were further divided by cancer type, specifically Multiple Myeloma (MM) (N= 64), non-MM hematological cancers (N=15), breast cancer (N=56), and other solid cancers (N=86). Details about specific cancer types in each group are listed in **Supplementary Table S1** and collected treatment information is listed in **Supplementary Table S2**. Demographic information, including age and sex of participants, are shown in **Table 1**. All participants received two (primary series) to five (primary and up to three booster) doses of mRNA SARS-CoV-2 vaccine as listed in **Table 1**. Due to the timing of participant enrollment, vaccination specifics (beyond number of doses) of most participants were only available for the last dose received. Consequently, when analyzing the effects of vaccine manufacturer, individuals were grouped based on the vaccine (mRNA-1273 from Moderna or BNT162b2 from Pfizer) of the last dose received. Sera from participants were collected and analyzed at 1-month post-last dose and 6-months post-last dose. In the two-dose cohort of this study, only samples from healthy participants and individuals with MM were available for the two-dose cohort.

**Table 1:** Demographics of study participants.

Participants	Hematological cancer			Solid cancer			Healthy Control	Total
	Total	Multiple Myeloma	Non-MM hematological	Total	Breast Cancer	Other solid		
N	79	64	15	142	56	86	352	573
Age								
Mean (SD)	65.2 (9.8)	65.9 (8.7)	62.0 (13.3)	63.7 (9.0)	62.4 (9.0)	64.5 (8.9)	47.0 (14.0)	53.4(14.7)
Range	28 - 84	43 - 84	28 - 81	41 - 81	43 - 76	41 - 81	19-77	19 - 84
Sex								
Female	44 (55.7%)	37 (57.8%)	7 (46.7%)	107 (75.4%)	56 (100.0%)	51 (59.3%)	258 (73.3%)	409 (71.4%)
Male	35 (44.3%)	27 (42.2%)	8 (53.3%)	35 (24.6%)	0 (0.0%)	35 (40.7%)	94 (26.7%)	164 (28.6%)
Race								
American Indian or Alaska Native	0 (0.0%)	0 (0.0%)	0 (0.0%)	0 (0.0%)	0 (0.0%)	0 (0.0%)	1 (0.3%)	1 (0.2%)
Asian	3 (3.8%)	3 (4.7%)	0 (0.0%)	3 (2.1%)	1 (1.8%)	2 (2.3%)	48 (13.6%)	54 (9.4%)
Black or African American	11 (13.9%)	11 (17.2%)	0 (0.0%)	2 (1.4%)	1 (1.8%)	1 (1.2%)	24 (6.8%)	37 (6.5%)
Caucasian	54 (68.4%)	39 (60.9%)	15 (100.0%)	136 (95.8%)	53 (94.6%)	83 (96.5%)	245 (69.6%)	435 (75.9%)
Multirace	0 (0.0%)	0 (0.0%)	0 (0.0%)	1 (0.7%)	1 (1.8%)	0 (0.0%)	14 (4.0%)	15 (2.6%)
Other	11 (13.9%)	11 (17.2%)	0 (0.0%)	0 (0.0%)	0 (0.0%)	0 (0.0%)	16 (4.5%)	27 (4.7%)
Unknown	0 (0.0%)	0 (0.0%)	0 (0.0%)	0 (0.0%)	0 (0.0%)	0 (0.0%)	4 (1.1%)	4 (0.7%)
Ethnicity								
Hispanic or Latino	3 (3.8%)	3 (4.7%)	0 (0.0%)	0 (0.0%)	0 (0.0%)	0 (0.0%)	24 (6.8%)	27 (4.7%)
Not Hispanic or Latino	23 (29.1%)	9 (14.1%)	14 (93.3%)	142 (100.0%)	56 (100.0%)	86 (100.0%)	328 (93.2%)	493 (86.0%)
Not Reported	53 (67.1%)	52 (81.2%)	1 (6.7%)	0 (0.0%)	0 (0.0%)	0 (0.0%)	0 (0.0%)	53 (9.2%)
Dose								
2	24 (25.8%)	24 (32.9%)	0 (0.0%)	0 (0.0%)	0 (0.0%)	0 (0.0%)	31 (7.4%)	55 (8.1%)
3	38 (40.9%)	34 (46.6%)	4 (20.0%)	26 (16.1%)	13 (20.3%)	13 (13.4%)	244 (58.0%)	308 (45.6%)
4	23 (24.7%)	13 (17.8%)	10 (50.0%)	41 (25.5%)	14 (21.9%)	27 (27.8%)	82 (19.5%)	146 (21.6%)
5	8 (8.6%)	2 (2.7%)	6 (30.0%)	94 (28.4%)	37 (57.8%)	57 (58.8%)	64 (15.2%)	166 (24.6%)

Data are n (%) where n represents the number of participants in each group. Due to rounding, not all variables add up to 100%.

### 2.2 Anti-SARS-CoV-2 IgG antibody levels after vaccination

**Figure 1:**
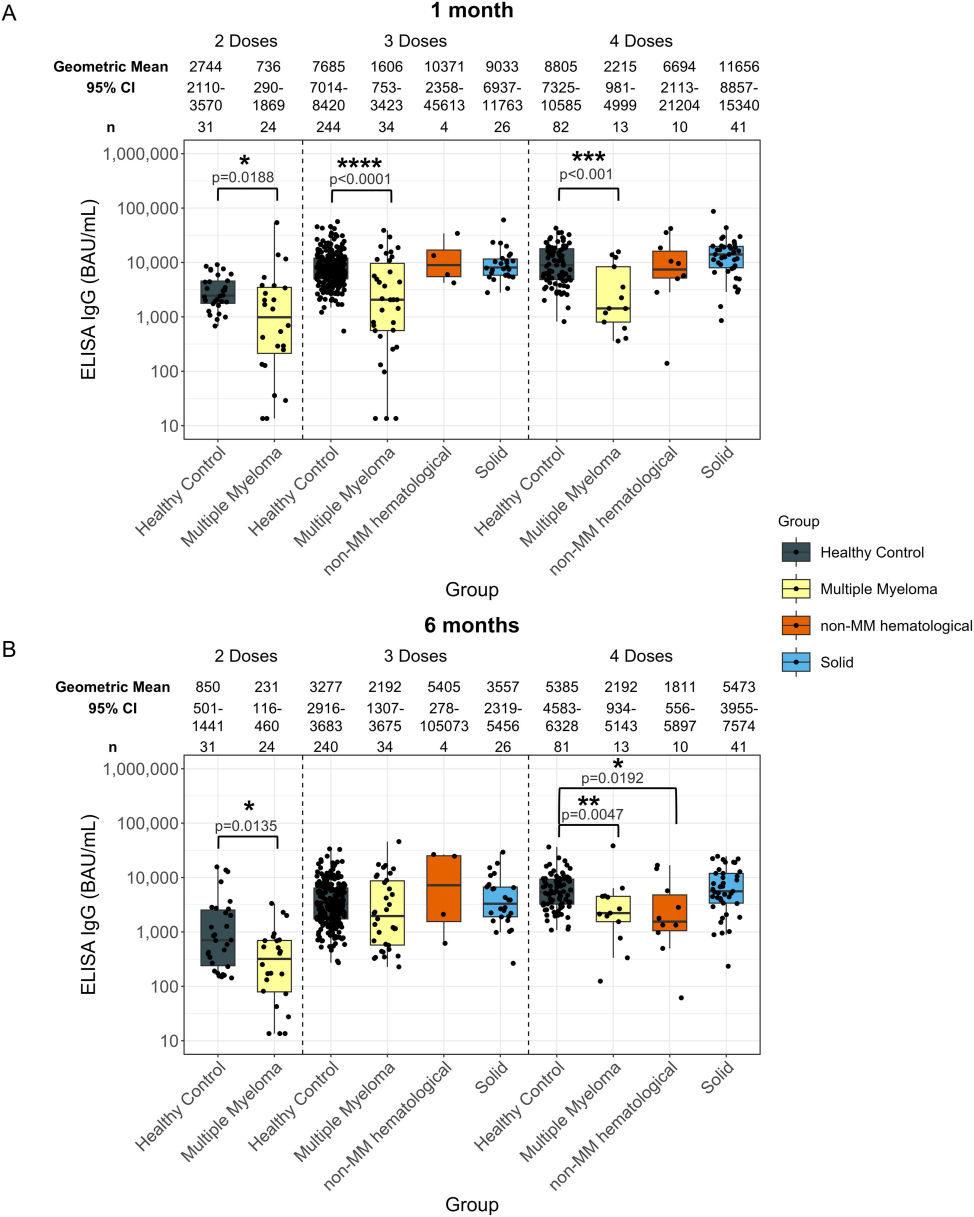
Anti-SARS-CoV-2 spike IgG levels post vaccination in serum samples from cancer and healthy cohorts. Comparison of serum anti-spike IgG levels in healthy, multiple myeloma, non-MM hematological cancer and solid cancer cohorts 1-month **(A)** and 6-months **(B)** post-vaccination. Anti-spike IgG levels were presented as Geometric Mean (GM) with 95% confidence intervals (95% CI). Box plots show median (horizontal bar), the first and third quartiles. Differences assessed by non-parametric Wilcoxon rank sum test. *p<0.05, **p<0.01, ***p<0.001, ****p<0.0001.

No samples were available in the solid cancer cohort for two doses at any timepoint. No significant differences in spike IgG levels were observed in the solid cancer cohort compared to healthy participants across any available time points (**Figure 1**). Additionally, when the solid cancer cohort was divided into the breast cancer subgroup and other solid cancer subgroups, no significant differences were noted compared to healthy participants (**Supplementary Table S3**).

Significantly lower anti-spike IgG levels were observed at 1-month post-second dose in individuals with hematological cancers (736 BAU/mL, p=0.0188) compared to healthy participants (2744 BAU/mL) (**Supplementary Figure S1A**). At 6-months post-second dose (**Supplementary Figure S1B**), individuals with hematological cancers had a significantly lower anti-spike IgG level at 231 BAU/mL (p=0.135) compared to 850 BAU/mL in healthy individuals 6-months post-second dose (**Supplementary Figure S1B**).

IgG levels at 1-month post-third and fourth dose in the hematological cancer cohort were 1954 BAU/mL (p<0.001) and 3582 BAU/mL (p=0.0102), respectively (**Supplementary Figure S1A**). These levels were both significantly lower than in the healthy cohort, which were 7685 BAU/mL 1-month post-third dose and 8805 BAU/mL 1-month post-fourth dose. No significant differences were observed in the individuals with hematological cancer 6-months post-third dose compared to healthy cohorts. Geometric mean IgG level at 6-months post-fourth dose was significantly lower with 2017 BAU/mL (p<0.001) in the hematological cancer cohort compared to 5385 BAU/mL in healthy participants (**Supplementary Figure S1B**).

The MM hematological cancer subcohort demonstrated the same trend as the hematological cohort, showing lower levels of anti-spike IgG levels at 1-month post-second (MM: 736 BAU/mL; healthy: 2744 BAU/mL; p=0.0188), third (MM: 1606 BAU/mL; healthy: 7685 BAU/mL; p<0.0001), and fourth (MM: 2215 BAU/mL; healthy: 8805 BAU/mL; p<0.001) doses, and at 6-months post-second (MM: 231 BAU/mL; healthy: 850 BAU/mL; p=0.0135), and fourth (MM: 2192 BAU/mL; healthy: 5385 BAU/mL; p=0.0047) dose (**Figure 1**). To minimize the influence of age and sex, analyses were also conducted using age- and sex- matched healthy controls. Similar trends in MM subcohort were observed, except for the post-second dose comparisons (**Supplementary Table S3**).

For the non-MM subcohort, significantly lower antibody responses were only observed at 6-months post-fourth dose of vaccine, with 1811 BAU/mL (p=0.0192) in the non-MM hematological cancer cohort compared to 5385 BAU/mL in healthy individuals (**Figure 1B**). However, the significance was not observed when compared to matched healthy controls (**Supplementary Table S3**).

No significant differences were observed in anti-spike IgG level between any cancer cohort and healthy participants who received five doses (**Supplementary Table S3**).

Anti-nucleocapsid IgG levels were also examined to determine the extent and influence of potential unreported prior COVID-19 infections (**Supplementary Table S5**). Significantly higher proportions of individuals with detectable anti-nucleocapsid IgG were observed in the MM subcohort at 1-month post-second dose (33.3%; n = 8 of 24), and at 6-months post-fourth dose (69.2%; n = 9 of 13). In contrast, 6.5% (n = 2 of 31) of healthy participants had IgG antibodies to nucleocapsid at 1-month post-second dose and 22.2% (n = 18 of 81) at 6-months post-fourth dose, suggesting a higher percentage of previous COVID-19 infections in the MM subcohort compared to healthy participants at these timepoints in this study. Despite the increased incidence of prior infections, these patients still showed diminished anti-spike IgG and avidity levels compared to healthy participants, as previously mentioned.

### 2.3 Anti-SARS-CoV-2 IgG antibody avidity levels after vaccination

**Figure 2:**
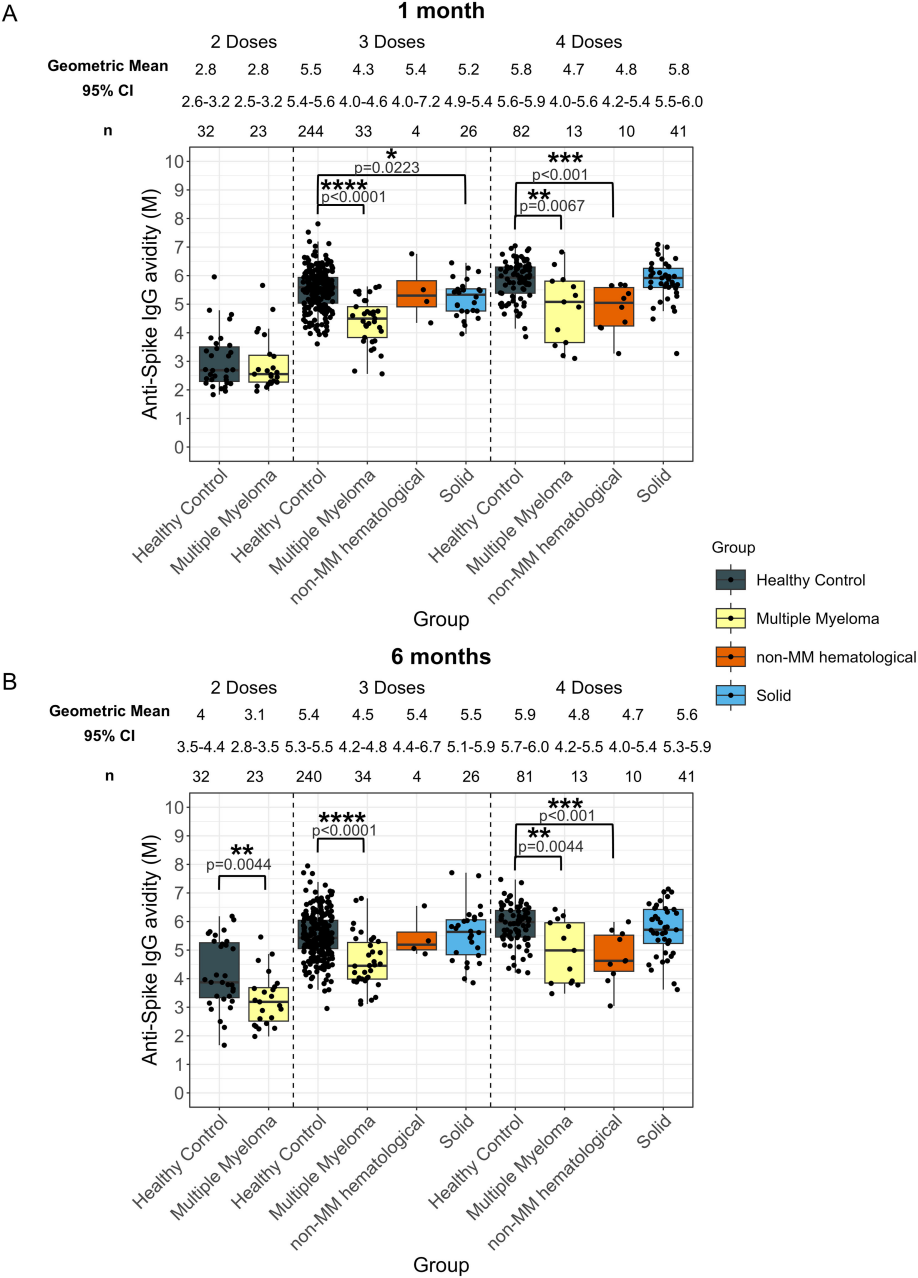
Anti-SARS-CoV-2 spike IgG avidity post vaccination in serum samples from cancer and healthy cohorts. Comparison of serum anti-spike IgG avidity in healthy, multiple myeloma, non-MM hematological cancer and solid cancer cohorts 1-month **(A)** and 6-months **(B)** post-vaccination. Anti-spike IgG avidity was presented as Geometric Mean (GM) with 95% confidence intervals (95% CI). Box plots show median (horizontal bar), the first and third quartiles. Differences assessed by non-parametric Wilcoxon rank sum test. *p<0.05, **p<0.01, ***p<0.001, ****p<0.0001.

**S**olid cancer cohort participants demonstrated lower serum IgG avidity to SARS-CoV-2 spike at 1-month post-third dose (5.2 M, p=0.0223) compared to healthy participants (5.5 M). No data was available for the solid cancer cohort after two doses, and there was no significant difference in avidity for the other doses or timepoints (**Figure 2**).

Individuals with hematological cancers did not exhibit significant differences in avidity at 1-month post-second dose (**Supplementary Figure S2A**); however, avidity levels at 6-months post-second dose were significantly lower (3.1 M, p=0.0044) compared to healthy participants (4.0 M) (**Supplementary Figure S2B**).

The hematological cancer cohort also showed significantly impaired avidity at 1-month post-third dose (4.4 M, p<0.0001) and post-fourth dose (4.7 M, p<0.0001) compared to the healthy cohort 1-month post-third dose (5.5 M) and 1-month post-fourth dose (5.8 M) (**Supplementary Figure S2A**). Patients with hematological cancers demonstrated 4.6 M (p<0.0001) 6-months post-third dose and 4.8 M (p<0.0001) 6-months post-fourth dose, which were consistently lower than healthy participants at 5.4 M 6-months post-third dose and 5.9 M post-fourth dose (**Supplementary Figure S2B**).

When the hematological cancer cohort was divided into the MM and non-MM hematological cancer subcohorts, impaired avidity was observed in the MM subcohort for all timepoints after three and four doses (**Figure 2**). Specifically, the MM cohort demonstrated significantly lower avidity at 1-month post-third dose (4.3 M, p<0.0001) and post-fourth dose (4.7 M, p=0.0067) and 4.5 M (p<0.0001) 6-months post-third dose and 4.8 M (p=0.0044) 6-months post-fourth dose compared to the healthy cohort at 5.5 M 1-month post-third dose and 5.8 M 1-month post-fourth dose and 5.4 M 6-months post-third dose and 5.9 M post-fourth dose (**Figure 2**). When compared to matched healthy controls, these trends in the MM cohort remained significant at 1-month, but were no longer significant 6-months post-fourth dose of vaccines (**Supplementary Table S4**). The non-MM hematological cancer subcohort demonstrated significantly lower avidity 1-month post-fourth dose (4.8 M, p<0.001) and 6-months post-fourth dose (4.7 M, p<0.001) compared to healthy participants with 5.8 M at 1-month and 5.9 M at 6-months post-fourth dose. When compared to matched healthy controls, the difference between non-MM cohort and healthy cohort was still significant at 6-months post-fourth dose but not at 1-month. No other significant differences between the non-MM subcohort and the healthy control cohort were observed across doses or timepoints (**Figure 2**).

No significant differences in anti-spike IgG avidity development were observed in any cancer cohort that received five doses as compared to the healthy group (**Supplementary Table S4**).

### 2.4 Anti-spike IgG level decay rate and avidity dynamics in the cancer cohorts

**Figure 3:**
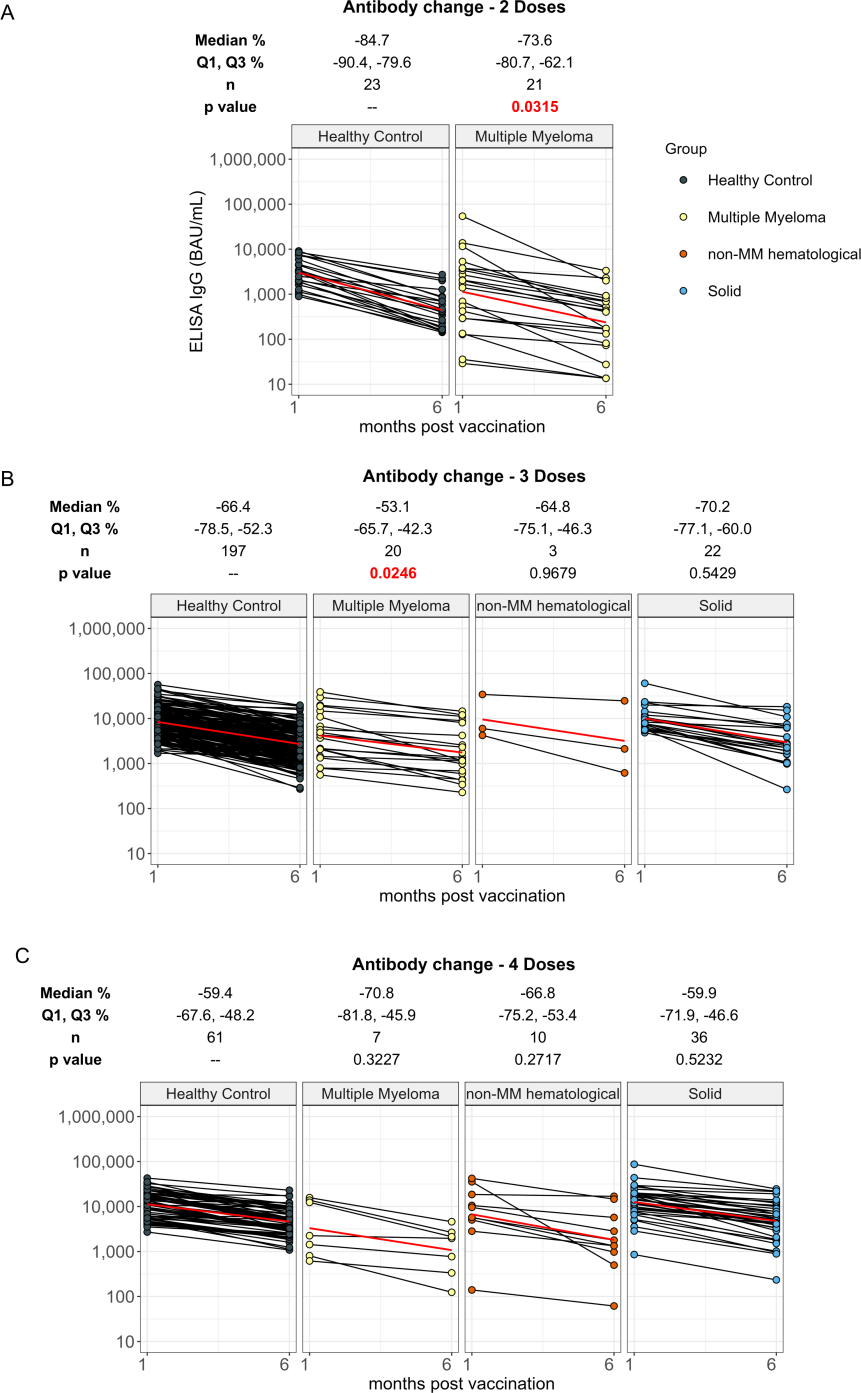
Percent change in anti-spike IgG levels from 1 month to 6 months post vaccination in cancer and healthy cohorts. Comparison of percent change in serum anti-spike IgG levels between 1 month and 6 months post-vaccination in individuals receiving 2 doses **(A)**, 3 doses **(B)** and 4 doses **(C)** of vaccine in healthy, multiple myeloma, non-MM hematological cancer and solid cancer cohorts. Percent change results were reported as Median with the first and third quartiles (Q1 and Q3). The p values indicate the significance levels of the percent change between 1 month and 6 months in the cancer cohort, compared to the percent change observed in the healthy cohort with the corresponding doses. Solid red line represents the connecting line of geometric mean titer at 1 month and 6 months in each cohort. Differences assessed by non-parametric Wilcoxon rank sum test.

No significant differences were observed for the serum IgG level decay rate from 1-month to 6-months between the solid cancer cohort and the healthy group across three or four doses (**Figure 3**).

Individuals with hematological cancer who received two doses exhibited significantly smaller decrease of anti-spike IgG levels from 1-month to 6-months, with a median percent change of 73.6% (p=0.0315) in the hematological cancer cohort compared to 84.7% in healthy participants (**Supplementary Figure S3A**). We also found a smaller decrease from 1-month to 6-months in hematological cancer cohort participants who received three doses when compared to the healthy participants with median percent changes of 55.4% and 66.4%, respectively (**Supplementary Figure S3B**). There was no significant difference in the decrease from 1-month to 6-months for the four-dose cohorts (**Supplementary Figure S3C**). The MM cohort exhibited the same trend as the greater hematological cancer cohort, with decreases of 73.6% (p=0.0315) for MM two-dose recipients compared to 84.7% for healthy participants (**Figure 3A**), 53.1% (p=0.0246) for MM three-dose recipients compared to 66.4% for healthy participants (**Figure 3B**), and no significant differences between the decreases for the four-dose cohorts (**Figure 3C**). The non-MM hematological cancer cohort did not show significant differences in IgG level decrease rates across doses.

**Figure 4:**
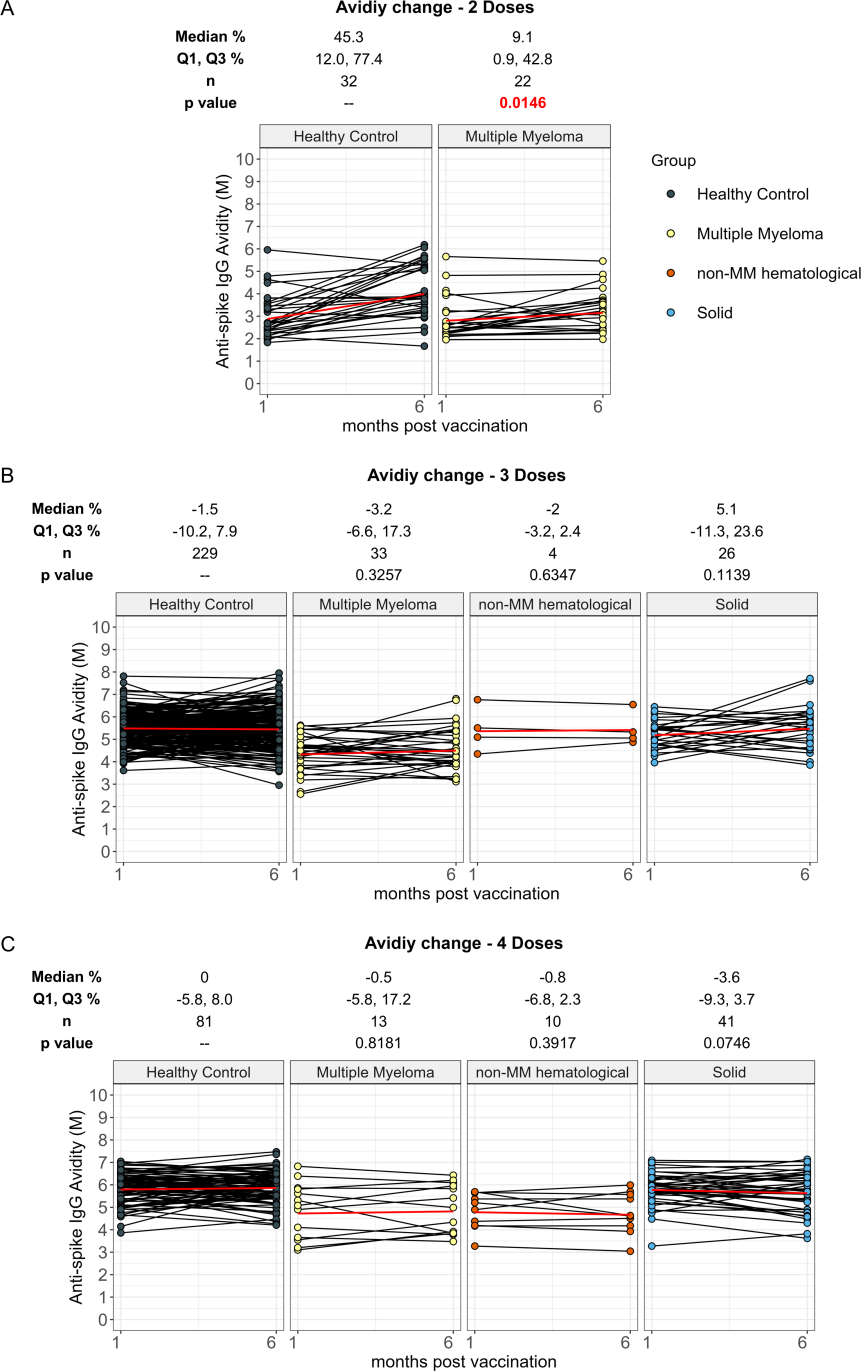
Percent change in anti-spike IgG avidity from 1 month to 6 months post vaccination in cancer and healthy cohorts. Comparison of percent change in serum anti-spike IgG avidity between 1 month and 6 months post-vaccination in individuals receiving 2 doses **(A)**, 3 doses **(B)** and 4 doses **(C)** of vaccine in healthy, multiple myeloma, non-MM hematological cancer and solid cancer cohorts. Percent change results were reported as Median with the first and third quartiles (Q1 and Q3). The p values indicate the significance levels of the percent change between 1 month and 6 months in the cancer cohort, compared to the percent change observed in the healthy cohort with the corresponding doses. Solid red line represents the connecting line of geometric mean titer at 1 month and 6 months in each cohort. Differences assessed by non-parametric Wilcoxon rank sum test.

Individuals with hematological cancers receiving two doses exhibited a significantly lower increase rate in avidity development from 1-month to 6-months with 9.1% (p=0.0146) in hematological cancer cohort participants compared to 45.3% in healthy participants (**Supplementary Figure S4A**). Avidity maturation was similar between hematological cancer cohort and healthy cohort receiving three doses and four doses of vaccine. No differences in avidity increases were observed for the MM and non-MM subcohorts except for the MM two-dose recipients, which make up the entire hematological cancer cohort for that dose number (**Table 1**, **Figure 4**).

### 2.5 Assessment of participant age, sex, and vaccine manufacturer effects on anti-spike IgG antibodies and avidity

#### 2.5.1 Influence of age

Cohorts were further divided and analyzed by age (younger defined as <65 years; older defined as ≥ 65 years old), sex (male or female), and vaccine manufacturer (mRNA-1273 from Moderna or BNT162b2 from Pfizer) of patients’ most recent dose to determine if these factors had any effect on IgG levels or avidity development.

**Figure 5:**
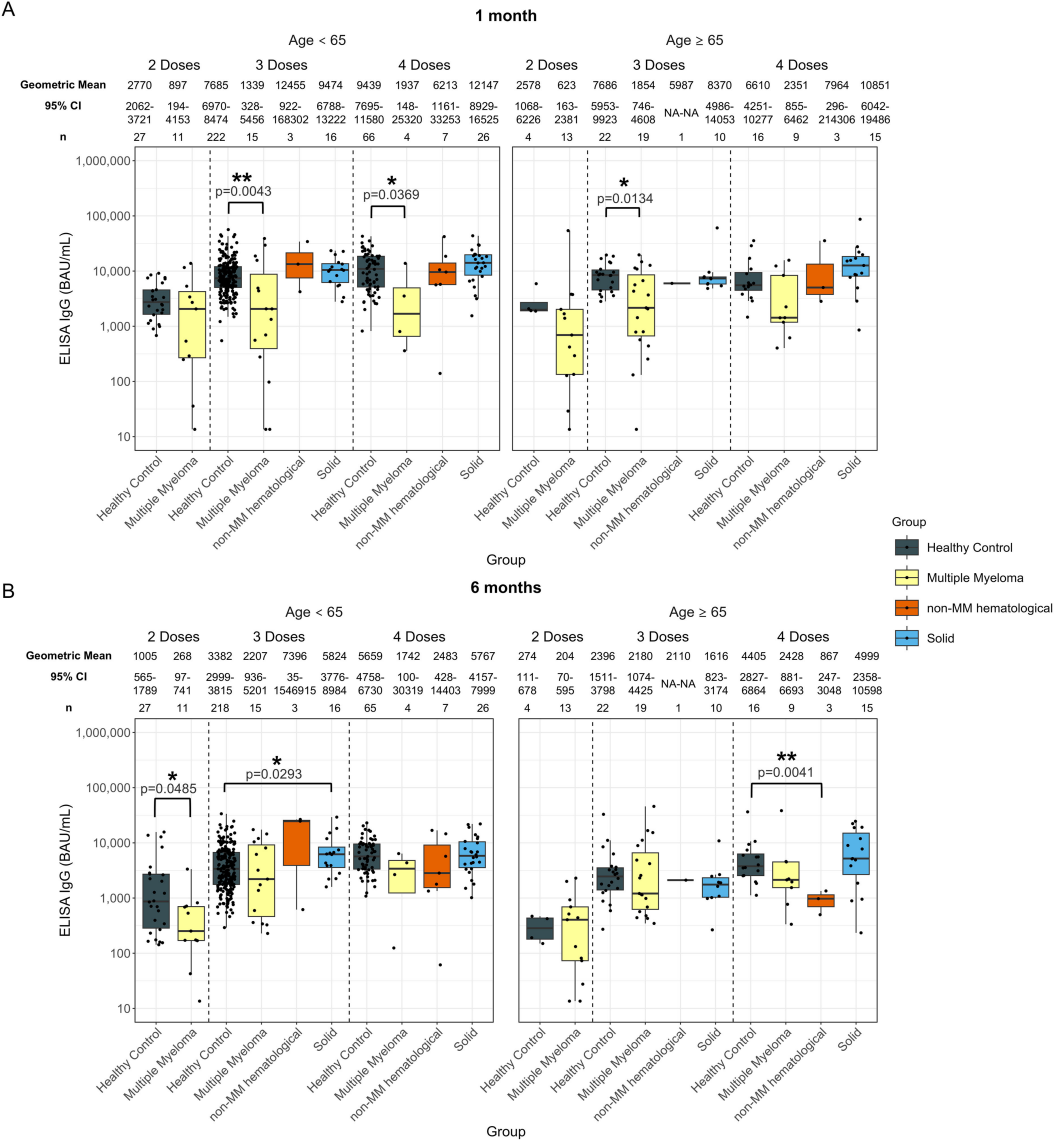
Anti-SARS-CoV-2 spike IgG levels post vaccination in serum samples from cancer and healthy cohorts within different age groups (<65 years old and ≥ 65 years old). Comparison of serum anti-spike IgG levels in healthy, multiple myeloma, non-MM hematological cancer and solid cancer cohorts within different age groups 1-month **(A)** and 6-months **(B)** post-vaccination. Anti-spike IgG levels were presented as Geometric Mean (GM) with 95% confidence intervals (95% CI). Box plots show median (horizontal bar), the first and third quartiles. Differences assessed by non-parametric Wilcoxon rank sum test. *p<0.05, **p<0.01.

Individuals with solid cancers in the younger group at 6-months post-third dose of vaccine showed higher antibody levels of 5824 BAU/mL (p=0.0293) compared to 3382 BAU/mL in healthy participants (**Figure 5B**). No other significant differences were observed for solid cancers at any age, dose, or timepoint (**Figure 5**).

In the hematological cohort, younger individuals with hematological cancers exhibited 268 BAU/mL (p=0.0485) at 6-months post-second dose, which was significantly lower than healthy individuals at 1005 BAU/mL (**Supplementary Figure S5B**). No other significant differences were observed for two-dose recipients at any age or timepoint (**Supplementary Figure S5**).

Anti-spike IgG levels at 1-month post-third dose of the vaccine were significantly lower than those of the healthy participants in both age groups, with 1941 BAU/mL (p=0.0258) in the younger hematological cohort and 7685 BAU/mL in the younger healthy cohort and 1966 BAU/mL (p=0.0130) in the older hematological cohort and 7686 BAU/mL in the older healthy cohort (**Supplementary Figure S5A**). There were no other significant differences in IgG levels between the hematological cancer and healthy cohorts for either age group at 6-months post-third dose (**Supplementary Figure S5B**).

At 6-months post fourth dose, older individuals with hematological cancers exhibited lower anti-spike IgG levels compared to healthy participants, with 1877 BAU/mL (p=0.0150) and 4405 BAU/mL, respectively (**Supplementary Figure S5B**). There were no other significant differences in IgG levels between the hematological cancer and healthy cohorts for either age group or timepoint post-fourth dose (**Supplementary Figure S5**).

Dividing individuals by both age and MM or non-MM resulted in relatively low sample sizes, limiting the power of the analyses. Younger patients with MM had levels of 1339 BAU/mL (p=0.0043) and older patients with MM had levels of 1854 BAU/mL (p=0.0134) at 1-month post-third dose compared to 7685 BAU/mL younger healthy participants and 7686 BAU/mL older healthy participants at 1-month post-third dose (**Figure 5A**). A significantly lower anti-spike IgG level was observed in older individuals with MM 1-month post-fourth dose; however, this cohort had a limited sample size of n = 4. Older individuals with non-MM hematological cancers in the older group at 6-months post-fourth dose also exhibited lower antibody levels compared to healthy participants; however, this cohort had a limited sample size of n = 3 (**Figure 5B**).

**Figure 6:**
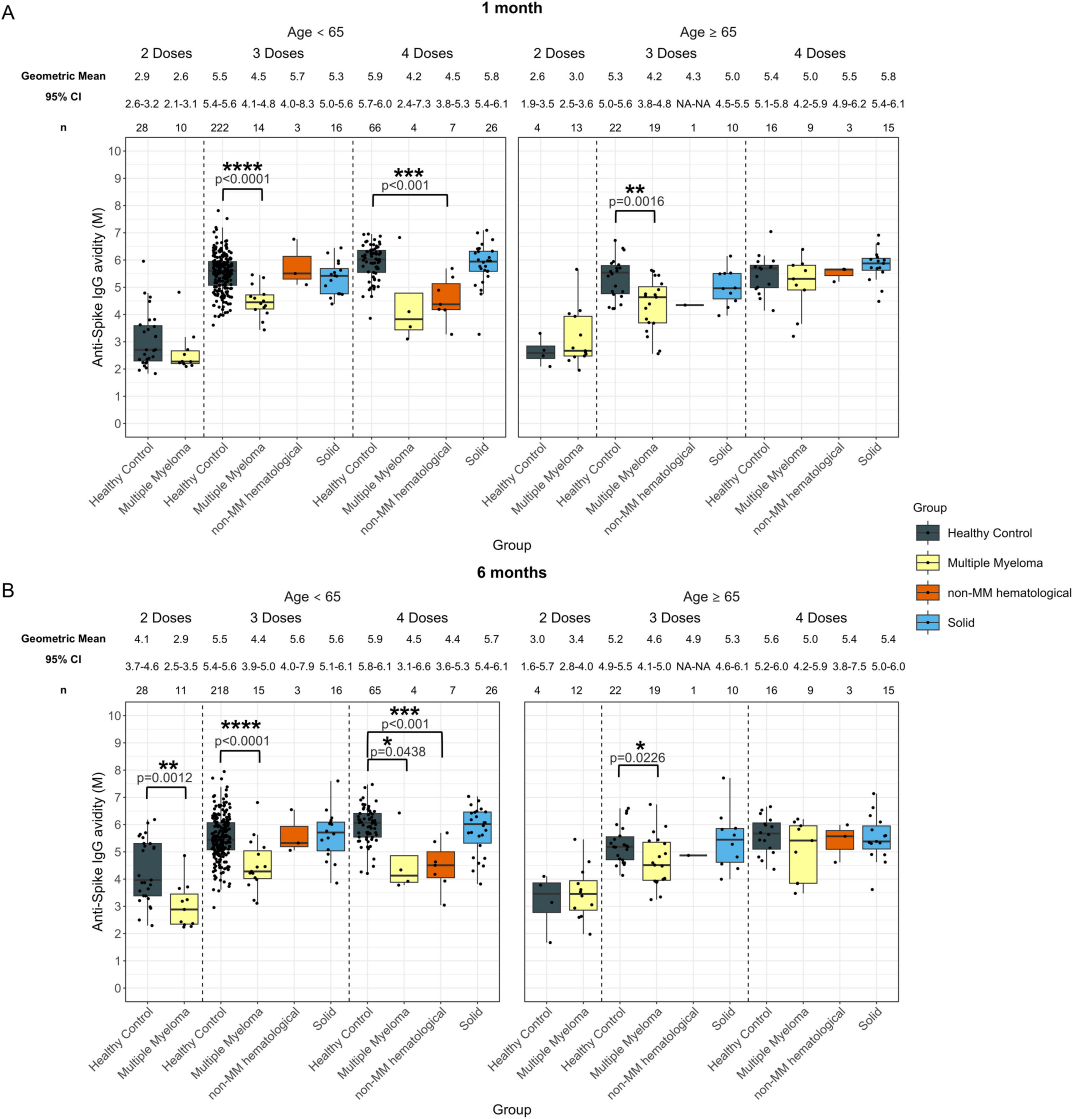
Anti-SARS-CoV-2 spike IgG avidity post vaccination in serum samples from cancer and healthy cohorts within different age groups (<65 years old and ≥ 65 years old). Comparison of serum anti-spike IgG avidity in healthy, multiple myeloma, non-MM hematological cancer and solid cancer cohorts within different age groups 1-month **(A)** and 6-months **(B)** post-vaccination. Anti-spike IgG avidity was presented as Geometric Mean (GM) with 95% confidence intervals (95% CI). Box plots show median (horizontal bar), the first and third quartiles. Differences assessed by non-parametric Wilcoxon rank sum test. *p<0.05, **p<0.01, ***p<0.001, ****p<0.0001.

No significant differences in avidity development were found in the solid cancer cohort compared to healthy participants in either age group across all vaccine doses at either timepoint (**Figure 6**).

In the younger hematological cohort, the avidity at 1-month post-third dose (4.7 M, p<0.0001), and post-fourth dose (4.4 M, p<0.001) were significantly lower than those of the healthy participants, which were 5.5 M after three doses and 5.9 M after four doses (**Supplementary Figure S6A**). In older individuals with hematological cancers, the avidity in patients who received three doses was significantly lower at 1-month (4.2 M, p=0.0012), compared to older healthy participants with 5.3 M at 1-month (**Supplementary Figure S6A**).

At 6-months, avidity development was consistently lower in younger individuals with hematological cancers for all doses (**Supplementary Figure S6B**) as compared to the healthy group. Avidity measurements in these younger donors were 2.9 M (p=0.0012) after two doses, 4.6 M (p<0.001) after three doses and 4.4 M (p<0.0001) after four doses, while younger healthy donor sera had avidity measurements of 4.1 M after two doses, 5.5 M after three doses, and 5.9 M after four doses (**Supplementary Figure S6B**). In older individuals with hematological cancers, the avidity in donors who received three doses was significantly lower at 6-months (4.6 M, p=0.0231), compared to the older healthy participants with 5.2 M at 6-months (**Supplementary Figure S6B**).

The MM subcohort exhibited similar trends to the hematological cohort except in younger patients at 1-month post-fourth dose, which were not significantly different from healthy participants, however this cohort had a limited sample size of n = 4 (**Figure 6A**). Additionally, younger individuals with non-MM hematological cancer exhibited 4.5 M (p<0.001) at 1 month and 4.4 M (p<0.001) at 6 months post-fourth dose, which were significantly lower than younger healthy individuals who had 5.9 M at 1 month and 5.9 M at 6 months (**Figures 6A, B**).

In addition, we investigated the dynamics of antibody levels and avidity within the same age group. While no significant differences in antibody levels were observed between cancer and healthy controls within each age group, younger MM patients showed a significantly lower increase in avidity following the second dose (10.2%, p=0.0368) compared to younger healthy individuals (46.2%). This difference was not observed in the older subgroup or following other vaccine doses (**Supplementary Table S6**).

#### 2.5.2 Influence of sex

**Figure 7:**
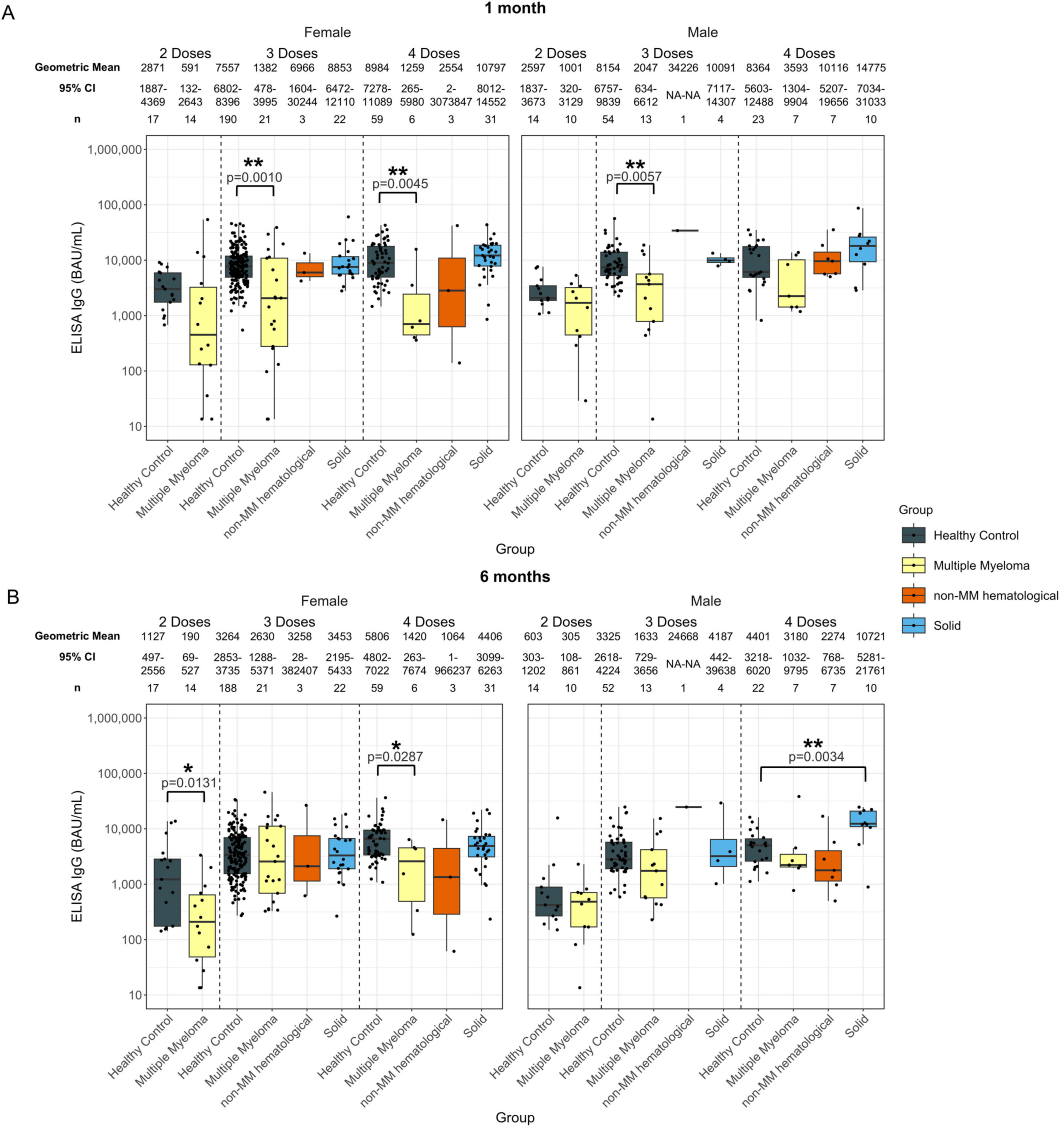
Anti-SARS-CoV-2 spike IgG levels post vaccination in serum samples from cancer and healthy cohorts within different sex groups. Comparison of serum anti-spike IgG levels in healthy, multiple myeloma, non-MM hematological cancer and solid cancer cohorts within different sex groups 1-month **(A)** and 6-months **(B)** post-vaccination. Anti-spike IgG levels were presented as Geometric Mean (GM) with 95% confidence intervals (95% CI). Box plots show median (horizontal bar), the first and third quartiles. Differences assessed by non-parametric Wilcoxon rank sum test. *p<0.05, **p<0.01.

Comparison of anti-spike IgG levels in females and males revealed notable differences by sex within each cohort (**Figure 7**). In individuals with solid cancers, significantly higher antibody levels were identified in males at 6-months post-fourth dose, with a geometric mean of 10721 BAU/mL (p=0.0034), compared to healthy males who had 4401 BAU/mL. No statistical significance was observed for females nor males at other timepoints or dose numbers (**Figure 7**).

No significant differences were observed post-second dose for any cohort except at 6-months in females with hematological cancers, who exhibited a lower response of 190 BAU/mL (p=0.0131) compared to healthy females who had 1127 BAU/mL (**Supplementary Figure S7**).

Significantly lower antibody responses were observed in females with hematological cancers 1-month post-third dose (1691 BAU/mL, p=0.0018), compared to healthy females (7557 BAU/mL) (**Supplementary Figure S7A**). Males with hematological cancers exhibited lower anti-spike IgG levels of 2504 BAU/mL (p=0.0234) at 1-month post-third dose as well, while the antibody levels in healthy males were 8154 BAU/mL (**Supplementary Figure S7A**). Significantly lower anti-spike IgG levels were also observed in females with hematological cancers at both 1-month and 6-months post-fourth dose, while the sample size was n = 9 (**Supplementary Figure S7**). However, no significance was identified in males with hematological cancers at 6-months post-fourth dose (**Supplementary Figure S7B**). When looking into specific cancer subtypes, similar trends were observed in the MM cohort, while no significance was observed for the non-MM hematological cancer cohort (**Figure 7**).

**Figure 8:**
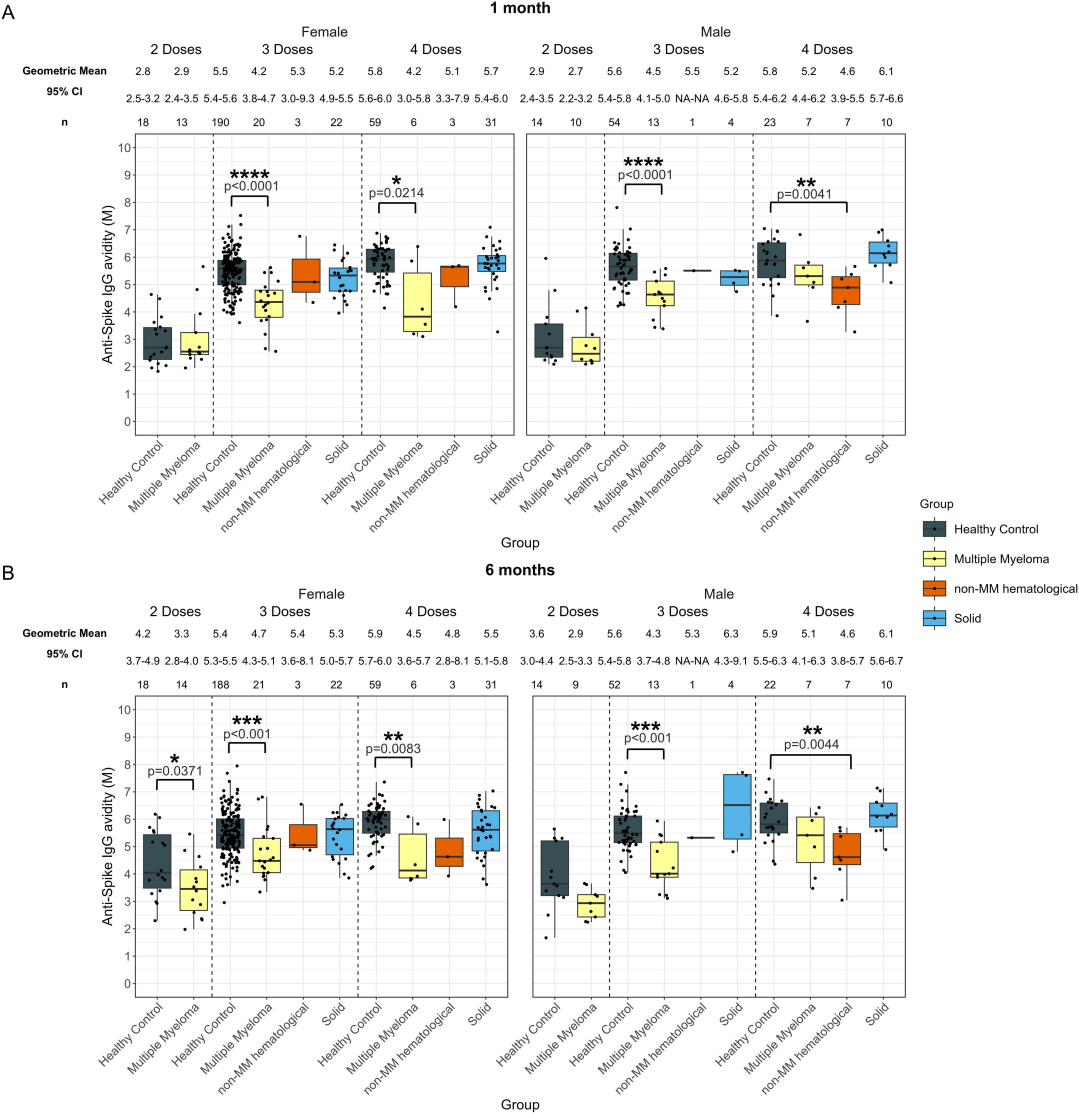
Anti-SARS-CoV-2 spike IgG avidity post vaccination in serum samples from cancer and healthy cohorts within different sex groups. Comparison of serum anti-spike IgG avidity in healthy, multiple myeloma, non-MM hematological cancer and solid cancer cohorts within different sex groups 1-month **(A)** and 6-months **(B)** post-vaccination. Anti-spike IgG avidity was presented as Geometric Mean (GM) with 95% confidence intervals (95% CI). Box plots show median (horizontal bar), the first and third quartiles. Differences assessed by non-parametric Wilcoxon rank sum test. *p<0.05, **p<0.01, ***p<0.001, ****p<0.0001.

No significant differences were found in the avidity measures of sera from the solid cancer cohort males or females when compared to healthy participants (**Figure 8**). There was no significant difference in avidity 1-month post-second dose for either females or males with hematological cancer. Significantly lower avidity was observed in females (but not males) with hematological cancer at 6-months post-second dose of vaccine (**Supplementary Figure S8B**). Significantly lower avidity was observed in the hematological cancer cohort in both sex groups at 1-month and 6-months following three and four doses of vaccine (**Supplementary Figure S8**). A similar trend was observed for the MM cohort except for males post-fourth dose, while males with non-MM hematological cancers post-fourth dose exhibited lower avidity with a limited sample size of n = 7 (**Figure 8**).

We also examined the dynamics of antibody levels and avidity by sex, within each cohort. Although antibody levels did not differ significantly between cancer cohorts and healthy controls within each sex group, female MM patients exhibited a significantly lower increase in avidity after the second dose (4.7%, p=0.0247) compared to female healthy individuals (58.8%), as shown in **Supplementary Table S7**.

#### 2.5.3 Influence of vaccine manufacturer

**Figure 9:**
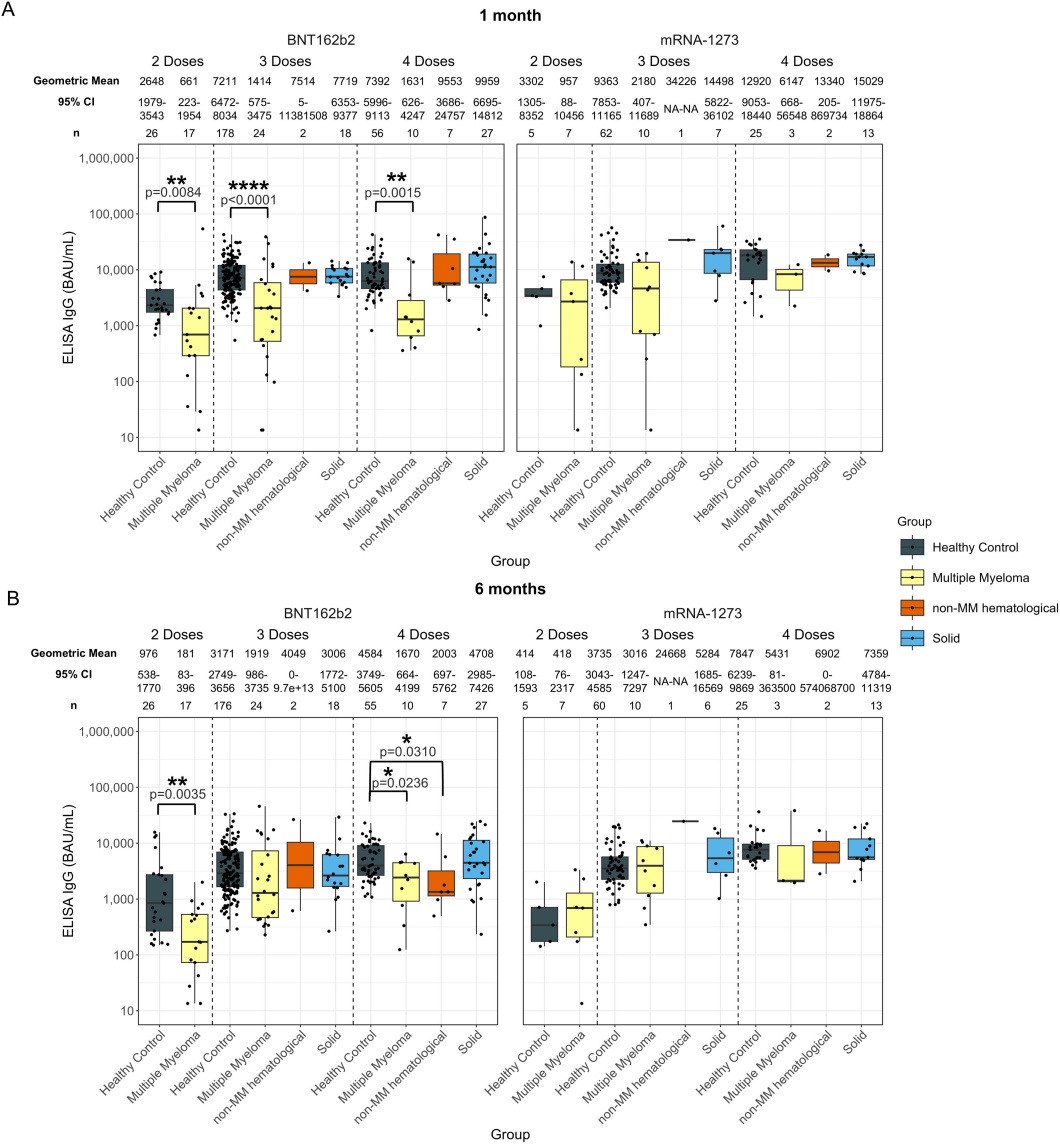
Anti-SARS-CoV-2 spike IgG levels post vaccination in serum samples from cancer and healthy cohorts within different vaccine groups. Comparison of serum anti-spike IgG levels in healthy, multiple myeloma, non-MM hematological cancer and solid cancer cohorts within different vaccine groups 1-month **(A)** and 6-months **(B)** post-vaccination. Anti-spike IgG levels were presented as Geometric Mean with 95% confidence intervals (95% CI). Box plots show median (horizontal bar), the first and third quartiles. Differences assessed by non-parametric Wilcoxon rank sum test. *p<0.05, **p<0.01, ****p<0.0001.

Anti-spike IgG levels and avidities were also studied in individuals based on their most recent dose for BNT162b2 or mRNA-1273 (**Figure 9**).

There was no statistical difference when comparing individuals with solid cancers to healthy individuals, regardless of the vaccine received (**Figure 9**).

Individuals with hematological cancers exhibited significantly lower antibody levels at 1-month in the BNT162b2 vaccine group for all doses compared to healthy participants (**Supplementary Figure S9A**). Specifically, at 1-month post-second dose, the anti-spike IgG level in the hematological cancer cohort was 661 BAU/mL (p=0.0084) compared to 2648 BAU/mL in healthy participants (**Supplementary Figure S9A**). Similarly, significantly lower antibody levels persisted in the hematological cancer cohort in the BNT162b2 group 1-month post-third dose (1608 BAU/mL, p<0.0001) and post-fourth dose (3377 BAU/mL, p=0.0411), while healthy individuals had 7211 BAU/mL and 7392 BAU/mL, respectively (**Supplementary Figure S9A**).

At 6-months, anti-spike IgG levels in people with hematological cancers in the BNT162b2 group were 181 BAU/mL (p=0.0035) after two doses and 1800 BAU/mL (p=0.0032) after four doses, both of which were significantly lower than those of healthy participants, who had 976 BAU/mL after two doses and 4584 BAU/mL after four doses. No significant differences were observed between the cancer cohorts compared to healthy participants 6-months post-third dose, and no significant differences were identified in individuals with hematological cancers who received the mRNA-1273 vaccine compared to healthy participants (**Supplementary Figure S9**).

Similar trends can be observed in the individuals with MM (**Figure 9**). Recipients of the BNT162b2 with non-MM hematological cancers did have significantly lower IgG levels at 6-months post-fourth dose of 2003 BAU/mL (p=0.0310) compared to healthy participants at 4584 BAU/mL (**Figure 9**).

**Figure 10:**
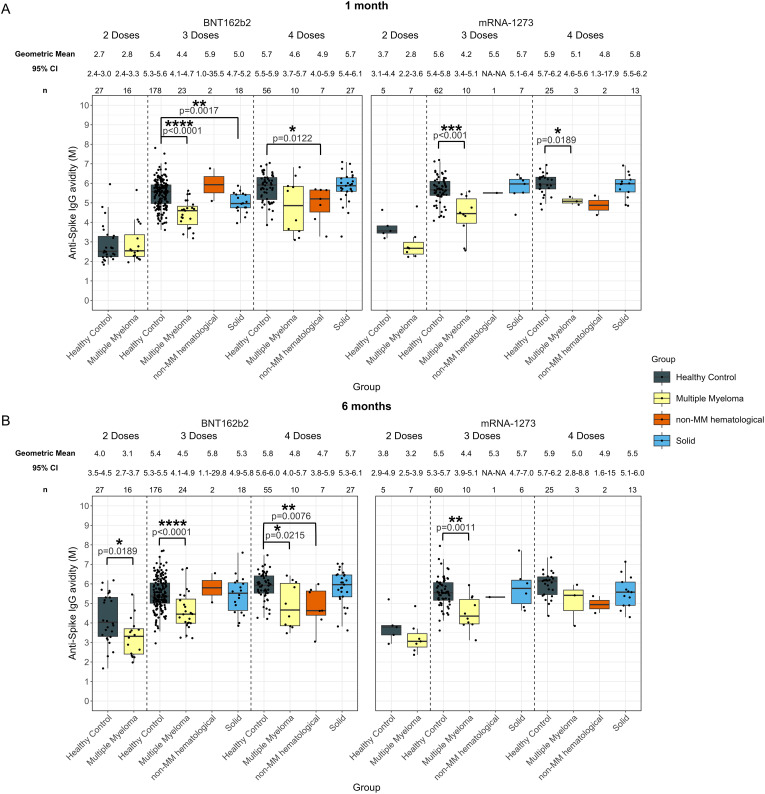
Anti-SARS-CoV-2 spike IgG avidity post vaccination in serum samples from cancer and healthy cohorts within different vaccine groups. Comparison of serum anti-spike IgG avidity in healthy, multiple myeloma, non-MM hematological cancer and solid cancer cohorts within different vaccine groups 1-month **(A)** and 6-months **(B)** post-vaccination. Anti-spike IgG levels were presented as Geometric Mean with 95% confidence intervals (95% CI). Box plots show median (horizontal bar), the first and third quartiles. Differences assessed by non-parametric Wilcoxon rank sum test. *p<0.05, **p<0.01, ***p<0.001, ****p<0.0001.

Individuals with solid cancers who received BNT162b2 vaccine showed significantly lower avidity at 1-month post-third dose (5.0 M, p=0.0017) than healthy participants (5.4 M). However, no significant differences were found in the solid cancer cohort in other doses and timepoints when compared to the healthy cohort (**Figure 10**).

Additionally, significantly lower avidity was observed in individuals with hematological cancer at 6-months post-second dose of BNT162b2 vaccine (3.1 M, p=0.0189), compared to healthy participants (4.0 M). No other significant differences were observed for either vaccine at either timepoint post-second dose (**Supplementary Figure S10**).

Significantly lower avidity was observed at both 1-month and 6-months following the third dose in the hematological cancer cohort in both vaccine groups (**Supplementary Figure S10**). In BNT162b2 group, the avidity in individuals with hematological cancer were 4.5 M (p<0.0001) at 1-month post-third dose and 4.6 M (p<0.0001) at 6-months post-third dose, while healthy participants had 5.4 M at 1-month and 5.4 M at 6-months. In the mRNA-1273 group, the avidity in individuals with hematological cancer were 4.3 M (p<0.001) at 1-month post-third dose, and 4.5 M (p=0.0013) at 6-months post-third dose, while healthy participants had 5.6 M at 1-month and 5.5 M at 6-months. Individuals with hematological cancer who received BNT162b2 vaccine also exhibited significantly lower avidity at both 1-month (4.7 M, p=0.0035) and 6-months post-fourth dose (4.7 M, p=0.0011) compared to healthy participants who had 5.7 M at 1-month and 5.8 M at 6-months (**Supplementary Figure S10**).

The MM cohort exhibited significantly lower avidity compared to healthy participants at 1- and 6-months post-third doses for both vaccines, 6-months post-second and post-fourth dose of BNT162b2 and 1-month post fourth dose of mRNA-1273 (**Figure 10**). No significant differences were observed in the non-MM hematological cancer group except in BNT162b2 recipients 1-month and 6-months post-fourth dose (**Figure 10**).

## 3 Discussion

The SARS-CoV-2 virus represented a major public health threat to the world, with a special impact for those diagnosed with immunocompromising conditions such as cancer. Studies have shown that people diagnosed with cancer are more likely to experience severe disease and even death due to COVID-19, and people with cancer appear to develop weaker immune responses to vaccination against SARS-CoV-2 compared to healthy recipients (1, 2, 39, 40). While additional doses of the mRNA vaccines do appear to help mitigate this difference, additional studies are needed to increase our understanding of the immune responses of people with cancer to multiple immunizations against COVID-19 (24, 25, 27). Several studies also showed lower seroconversion rates and inferior antibody levels in individuals with hematological cancers compared to those with solid cancers and healthy individuals after primary vaccination and after a booster dose (41–44). The advent of additional doses of the COVID-19 vaccine to mitigate waning immunity and the development of more recent and targeted vaccines to circulating viral variants present additional questions concerning the safety and efficacy of repeated mRNA vaccinations, especially since initial safety and efficacy assessments did not explore the effects in patients with cancer (8, 9, 11, 12, 14, 15, 18). Comprehensive assessments of immune responses in individuals with cancer to multiple mRNA vaccinations are key to inform vaccine recommendations for these populations. This study aims to investigate certain knowledge gaps by evaluating circulating antibody responses (levels and avidity of anti-SARS-CoV-2 antibodies) following mRNA vaccines, as well as their durability, in both cancer and healthy cohorts at multiple time points following vaccination.

Our findings show an overall trend of consistently lower anti-spike IgG levels in individuals with hematological cancers across multiple doses and different timepoints following vaccination compared to healthy individuals, indicating impaired immune responses to the COVID-19 vaccines. However, these differences were not observed in the solid cancer cohort. These results are in line with other studies, which found that patients with hematological cancers consistently exhibit lower immune response to COVID-19 vaccines compared to patients with solid cancers, and that patients with solid cancers had no significant difference in immune response compared to healthy participants following a third dose of vaccine (29, 45–48).

Lower avidity levels were observed at 1-month after vaccination in sera from participants with hematological cancers who received three and four doses of vaccine compared to healthy individuals. By 6-months, avidity in the hematological cancer patients was significantly lower across all vaccine doses (two, three, and four doses). Solid cancer patients developed avidity levels after vaccination comparable to the healthy population. A similar trend was observed in the MM cohort, which was the main subset of the hematological cancer cohort. In individuals with solid cancer, comparable avidity development was observed across different immunizations and timepoints except for lower avidity at 1-month following three doses compared to healthy controls.

Studies have shown that the effectiveness of currently available vaccines decrease with time, and this decrease correlates in part with decreasing serum IgG levels. In contrast, avidity typically increases with time, potentially due to a “honing” of the immune response where increasingly scarce antigen selects for B cells with the ability to bind tightly to the target antigen (49). A protective humoral response would depend upon not only the quantity of circulating antibody, but also the quality of those antibodies. In this report, we investigated whether people with cancer experience similar rates of decreasing serum IgG antibody content compared to the healthy vaccine recipients and found that people with hematological cancers demonstrated a significantly smaller decrease in IgG levels over 6 months after two or three doses. Similar observations were made within the MM cohort when it was separately analyzed from the hematological cancer cohort. The MM cohort appeared to be the main driver of the findings, as it was the major hematological cancer type in our study. There were no significant differences in the decay rates after two, three or four doses in the solid cancer cohort when compared to healthy controls. These results suggest that people with hematological (particularly MM), but not solid cancers, experience a lower decrease rate in immunity after vaccination compared to the healthy population. These findings may be partly related to undocumented exposures of the virus that may have led to a more long-lasting response. In addition, the lower decrease rate in MM cohort compared to the healthy cohort could reflect their lower peak antibody responses post-vaccination, as individuals with higher initial responses could exhibit a steeper relative decline (50, 51).

Although serum anti-spike IgG levels decayed over time, anti-spike IgG avidity increased from 1-month to 6-months in the two-dose group, where individuals with hematological cancer exhibited a significantly lower avidity increase rate compared to healthy controls. As a result, while avidity was similar at 1-month post-second dose, it was significantly lower at 6-months in the hematological cancer cohort when compared to healthy controls. Avidity levels remained stable and relatively flat from 1 to 6-months in both the three and four-dose groups, in the hematological cancer and the healthy cohorts, although avidity was higher in the healthy group. No significant differences in avidity change were found between the solid cancer and the healthy cohorts. The different avidity dynamics in the two-dose group and the booster-dose groups (both three- and four-dose groups) indicate enhanced avidity over time, with repeated exposure to antigen and possibly cross-reactivity following booster vaccination. Previous studies have suggested that booster doses further expand memory B cell pool, leading to enhanced cross-reactivity and cross neutralizing capacity against diverse SARS-CoV-2 variants, including Omicron (52). However, in this study we did not assess cross-neutralization or variant-specific binding.

The distinct patterns of the serological response between individuals with hematological cancers and individuals with solid cancers could be associated with the immune dysregulation resulting from the cancer type and also, the different treatment approaches. For example, one study found that individuals with chronic lymphocytic leukemia (CLL) had the lowest response to primary series vaccination among different hematological cancers. In this study, individuals with MM also showed reduced response rates while individuals with Hodgkin lymphoma had the highest response rate compared to other hematological cancer cohorts (53). These results may be explained in part by the disease-associated immunosuppression related to the inhibition of B-cell expansion and dysfunctional antigen presentation in individuals with CLL or MM (54–56). Some data indicates that, in contrast to individuals with hematological cancers, individuals with solid cancers have similar immune landscape to healthy individuals following SARS-CoV-2 infection. Specifically, less impairment of B cell and CD4+ T cells have been observed in individuals with solid cancers compared to individuals with hematological cancers (57). Solid cancer subtypes could play a role in the immune response to vaccination as well. Some studies have reported that individuals with lung cancer could have significantly lower antibody responses to vaccination than breast cancer patients, which could in part be attributed to hormonal influences on immune function (58). The solid cancer cohort in our study includes a substantial number of individuals with breast cancer, which could influence the overall findings of the serological response in the solid cancer cohort. Besides the cancer type, active treatment can also be responsible for weakened immune responses. B cell subset perturbations have been reported to be more pronounced during active therapy (59). B cell depleting therapies, such as anti-CD38 and anti-BCMA treatment commonly used in treating MM, have shown a detrimental effect on antibody levels and may result in impaired B cell differentiation, decreased plasmablasts and plasma cells and compromised T cell response (60–62). Treatment information in our study was very limited, and therefore, we cannot exclude the possibility that some patients in the hematological cohort had received such therapies, which may have contributed to the impaired antibody responses observed. Moreover, one study suggested that preserved CD8+ T cells in these individuals with B-cell deficiency could possibly help reducing mortality (57).

While the overall trends were similar when comparing cancer cohorts to the general healthy group, some differences were no longer significant when comparisons were restricted to age- and sex- matched healthy controls. Given that advanced age and male sex are known risk factors for higher mortality in COVID-19 infection within the general population, results were further stratified by age and sex (63, 64). According to CDC, more than half of the COVID hospitalizations occurring between October 2023 and December 2023 occurred in older adults, and additional boosters are recommended for those aged 65 years or older (28, 65). An age-dependent decrease of vaccine-induced neutralizing antibodies has been reported in older adults and the same trend has been found for spike-specific IgG memory B cells and CD4+ T cells and CD8+ T cells (66). Dietz et al. also reported a similar decrease in anti-spike IgG level and CD4+ T cells in older individuals (67). However, the influence of vaccine doses and age has not been well characterized in cancer patients. Our results showed that both younger (< 65 years old) and older (≥ 65 years old) individuals with hematological cancers exhibited lower anti-spike IgG antibody 1-month after receiving three doses, but by 6-months, their levels were not significantly different from those of healthy controls, which indicated that three doses of vaccination might help maintain anti-spike IgG levels in the individuals with hematological cancers. Additionally, older individuals with hematological cancers had significantly lower antibody responses than those of the healthy cohort 6-months after receiving four doses, suggesting that additional boosters might not be as effective in maintaining antibody levels in the older hematological cancer cohort. As for avidity, younger individuals in the hematological cohort who received three doses and four doses showed lower avidity at both 1-month and 6-months. This same trend was seen in these patients at 6-months after receiving two doses. In contrast, older patients demonstrated lower avidity at both time points only when receiving three doses. Similar trends were observed in the MM cohort. In contrast to the hematological cancer cohort, individuals with solid cancers, showed comparable antibody levels and avidity in both age groups. When receiving three doses, they exhibited even higher levels of anti-spike IgG than healthy individuals in the younger age group at 6-months.

When data was stratified by sex, lower anti-spike IgG levels were observed in both females and males with hematological cancers 1-month after three doses of vaccine. By 6-months, lower antibody levels were observed in females who received two doses or four doses, but not in males. Both female and male hematological cancer cohorts showed lower avidity than the healthy cohort at three and four doses. A similar trend was observed in MM cohort. Individuals with solid cancers demonstrated antibody levels and avidity similar to or sometimes even higher than those of healthy individuals. Male sex is identified as a risk factor associated with higher COVID-19 mortality (68). Females generally exhibit higher antibody responses to various vaccinations, including COVID-19 (69–71). There is a lack of studies comparing immune responses to vaccination in individuals with cancer and healthy controls, stratified by sex. Our results indicate that there is a diminished antibody response to SARS-CoV-2 vaccine in females with hematological cancers post-fourth dose when compared to healthy females; a relationship that was not observed in males. These results may indicate that vaccination strategies may have to be optimized for cancer and immunocompromised patients, taking into account age and sex to optimize efficacy. In addition, we observed that younger and female healthy individuals showed significantly higher avidity increases after the second dose, compared to individuals with MM, but this difference was not observed in the older or male subgroups. This is consistent with evidence that age and sex are determinants of the immune response to vaccines and suggests that these factors may influence antibody function.

We also investigated the impact of the two mRNA vaccines approved in the US on the serological response in individuals with cancer based on their most recent vaccine dose. mRNA-1273 has been shown to induce higher anti-spike antibody levels in healthy individuals and cancer patients when compared to the BNT162b2 vaccine (21, 72, 73). In our study, consistently lower responses were observed in individuals with hematological cancers, particularly those with MM, in the BNT162b2 group. Within the mRNA-1273 group, no significant differences were observed in individuals with hematological cancers compared to healthy participants at both 1-month and 6-months, potentially due to the small sample size in the mRNA-1273 group. Additionally, no significant differences were identified between individuals with solid cancers and healthy individuals in either vaccine groups. In terms of avidity, sera from the hematological cancer cohort, receiving either vaccine, showed significantly lower avidity compared to the healthy individuals, but there were no differences in the patterns observed between the two vaccines in the hematological cancer cohort.

Despite these important findings, this study has some limitations. In various groups, sample sizes were modest, particularly for the non-MM hematological cancer cohort and mRNA-1273 cohort. The sample number became even smaller when we further stratified each cohort by age and sex. Thus, future studies with larger numbers are warranted. Furthermore, when the influence of vaccine manufacturer was investigated, only information on most recent vaccine dose was available for most participants, which limited the power of the analyses and the ability to determine the extent to which variant-adapted vaccines contributed to the observed immune responses, particularly in later time points among cancer patients. In addition, cancer treatment information was not consistently available to allow us to evaluate the influence of cancer treatment on immune responses to vaccination. A more comprehensive and standardized collection of treatment data will be essential in future studies to evaluate how different cancer therapies influence the quality and durability of vaccine responses. Moreover, our study was limited to binding assays based on the ancestral spike protein, which have cross-reactivity across different variants, but do not differentiate between variant-specific responses. Future studies including evaluation of neutralizing antibody responses and cellular immune response analyses targeting the relevant variants will be important to understand how functional and cellular variant-specific responses may be affected in cancer populations.

This study contributes to a better understanding of how cancer type and key determinants of immune response such as age and sex influence the antibody response (both level and quality) to two, three, and four doses of vaccine at two different time points post-administration. Our comparisons between cancer cohorts and healthy individuals demonstrated that individuals with hematological cancers, especially with MM, exhibited diminished anti-spike IgG level and avidity across two, three and four doses compared to healthy individuals. Meanwhile, comparable antibody levels and avidity were observed in individuals with solid cancers following different doses of boosters. Our study also demonstrated that the patterns of immune response when comparing the hematological cohort to healthy controls may vary based on factors such as age, sex, and vaccine, highlighting the complexity of immunogenicity and vaccine efficacy in cancer patients. Our findings align with various studies showing that individuals with hematological cancers have a reduced response to COVID-19 vaccines (74–76). The marked difference in serological response to vaccination between individuals with hematological and solid cancers highlights the higher risk of infection and disease severity faced by individuals with hematological cancers. Additional studies are needed to investigate if these patients may benefit from alternative vaccine regimens or personalized vaccination strategies to achieve adequate protection.

## 4 Materials and methods

### 4.1 Samples

Serum samples from cancer cohort (n = 221) were obtained through three SeroNet capacity building centers (CBC) including University of Minnesota (Medical Protocol HRP-590), Icahn School of Medicine at Mount Sinai (Protocol Number STUDY-16-01215), and the Feinstein Institutes for Medical Research (Institutional Review Board #20-1007) and the samples were collected under approved protocols. Serum samples from healthy donors (n = 352) were drawn from these same three sites as well as from Occupational Health Services, Frederick National Laboratory for Cancer Research, Frederick MD, under the Research Donor Protocol OH99CN046. Participants were enrolled between January 2021 and January 2023, and sample collection occurred between January 2021 and August 2024. Most sample collections occurred approximately 1 month after completion of the primary series or booster doses, followed by additional collections around month 6 post-vaccination. Specifically, sera from participants were collected around 1-month post-vaccination (Range: 9-52 days; Mean ± SD: 33.8 ± 9.1 days) and around 6-months post-vaccination (Range: 158-202 days; Mean ± SD: 182.0 ± 7.9 days). No samples were available in solid-tumor cancer cohort for two doses at any timepoint.

### 4.2 Sample preparation

Peripheral blood samples were collected by venipuncture using serum separator tubes. Blood was allowed to clot upright at room temperature for 30-60 minutes, then centrifuged at 1,300 × rcf for 20 minutes at room temperature. The serum was carefully removed, aliquoted into sterile cryovials. Samples were immediately stored in a -80°C freezer following processing. Blood collected in serum tubes was processed and frozen the same day as collection. The serum samples were kept at -80°C until testing. Prior to testing, the samples were aliquoted and heat-inactivated at 56°C for 30-60 minutes to minimize freeze-thaw events of the sample.

### 4.3 Enzyme-linked immunosorbent assays

Anti-SARS-CoV-2 spike IgG levels in sera were measured using standardized enzyme-linked immunosorbent assays (ELISA) in all cancer groups and were compared to levels measured in the healthy control cohort. ELISA assay used in this study was based on the full-length spike protein of the original Wuhan SARS-CoV-2 strain. Anti-spike IgG levels were presented as Geometric Mean (GM) with 95% confidence intervals (95% CI).

ELISA assays used to quantify human serum IgG antibodies to the SARS-CoV-2 antigens were performed at room temperature as follows: Maxisorp 96-well plates (Thermo-Scientific Cat# 439454) were coated with 100 μL recombinant SARS-CoV-2 Spike protein sourced from the Protein Expression Laboratory at Frederick National Laboratory for Cancer Research (FNLCR) at a concentration of 0.3 µg/mL in phosphate-buffered saline (PBS). After coating for a minimum of 24 h at 4°C, assay plates were washed with 300 μL PBS-Tween buffer three times and blocked with 300 μL PBS-Tween 0.2% and 4% skim milk (BD, Cat# 232100) for 90 minutes (min) to minimize nonspecific binding. After the blocking buffer was removed, heat-inactivated samples were tested with appropriate in-well dilution series. Plates were incubated for 60 min with the samples at room temperature. Subsequently, the plate was washed and then incubated for 60 min with 100 μL goat anti-human IgG HRP-conjugate (Seracare, Cat# 5220-0390, Milford, MA, USA) at room temperature. Following the incubation, the plate was washed, and then developed with 100 μL 3,3,’5,5’-tetramethylbenzidine (TMB) 2-component substrate (Seracare, Cat# 5120-0049, 5120-0038) for 25 min. The reaction was stopped with 100 μL 0.36 N sulfuric acid and the absorbance was read at 450 nm and 620 nm using a SpectraMax plate reader (Molecular Devices, San Jose, CA, USA). Data analyses were performed using SoftMax Pro GxP 7.0.3. Reportable values for IgG quantitative ELISA are binding antibody units per milliliter (BAU/mL), based on a standard calibrated to the World Health Organization (WHO) International Standard (77).

### 4.4 Avidity enzyme-linked immunosorbent assays

The avidity of serum SARS-CoV-2 spike IgG antibodies was assessed with a chaotrope ELISA. Avidity assay used in this study was based on the full-length spike protein of the original Wuhan SARS-CoV-2 strain. Anti-spike IgG avidity results were presented as Geometric Mean (GM) with 95% confidence intervals (95% CI).

Avidity ELISA assays (chaotrope ELISA) are based on standard ELISA tests for anti-SARS-CoV-2 Spike IgG but include an additional step where bound analyte (antibody) is exposed to a chaotropic agent that effectively breaks and elutes off weakly bound antibody species: a “bind and break” ELISA. Urea was used as the chaotropic agent in this study due to its experimental range and minimal impact on the integrity of the assay plate coating. ELISA assays to assess serum avidity were conducted with samples that upon dilution in assay buffer produced optical densities (OD) in a standard IgG ELISA of between 0.5 and 1.3 OD units at 450 nm; with a target OD of 1.0. Each assay plate tested five serum samples in duplicate. After each sample was incubated on the assay plate for 60 min at room temperature, the plates were washed and incubated with dilutions of urea ranging from 0 to 10 molar (M) for 15 min at room temperature, followed by four washes in PBS-Tween. The plate was further developed as described for the quantitative IgG assay, continuing with the conjugate antibody. Serum avidity assessments are reported as Avidity Indices (AI80), the molar concentration (M) of chaotrope required to reduce the optical density of the sample to 80% that of untreated wells. Additionally, each assay plate contained two system suitability participants that were developed from well characterized serum samples: one control with a known low avidity index, the other a known high avidity index.

### 4.5 Data analysis

Cancer cohorts were compared to healthy participants using the non-parametric Wilcoxon rank sum test due to a lack of normality in the data. Analyses were performed separately for each dose with all participants. The effects of participant age, sex, and vaccine manufacturer on antibody level and avidity were investigated for each dose separately. Since the effects of age, sex, and vaccine manufacturer were not specific outcomes of interest, only within group comparisons of the cancer cohorts and participants were modeled and across group comparisons were not considered. Given that individuals aged 65 or older represent the majority of current COVID-19 hospitalizations and typically developed lower antibody levels after vaccination, we divided the healthy and cancer cohort into different age groups: the younger group (< 65 years old) and the older group (≥ 65 years old) (**Figure 5**) (65, 78). Change in response from 1-month to 6-months was modeled using percent change as a single data point for each participant at a given dose. Anti-spike IgG levels and avidity results were presented as Geometric Mean with 95% confidence intervals (95% CI). Percent change results were reported as Median with the first and third quartiles (Q1 and Q3).

## Data Availability

The original contributions presented in the study are included in the article/**Supplementary Material**, Further inquiries can be directed to the corresponding author.
