# Supplementary material for: Circulating SARS-CoV-2 spike IgG antibody responses in cancer patients following multiple COVID-19 vaccination boosters
Source: Front Immunol. 2025 Aug 12;16:1629473. doi: 10.3389/fimmu.2025.1629473 (PMC12378708; doi:10.3389/fimmu.2025.1629473)
Supplement: Supplementary file 1 [file DataSheet1.docx]

Supplementary Material

# Supplementary Tables

## Supplementary Table 1. Cancer Types

|  | **Breast Cancer (N=56)** | **Non-MM Hematological (N=15)** | **Multiple Myeloma (N=64)** | **Other Solid Cancer (N=86)** | **Total (N=221)** | **p value** |
| --- | --- | --- | --- | --- | --- | --- |
| **Harmonized Cancer Name** |  |  |  |  |  | < 0.001 |
|  |  |  |  |  |  |  |
| Acute Promyelocytic Leukemia | 0 (0.0%) | 1 (6.7%) | 0 (0.0%) | 0 (0.0%) | 1 (0.5%) |  |
| Appendiceal Cancer | 0 (0.0%) | 0 (0.0%) | 0 (0.0%) | 2 (2.3%) | 2 (0.9%) |  |
| Basal Cell Carcinoma | 0 (0.0%) | 0 (0.0%) | 0 (0.0%) | 4 (4.7%) | 4 (1.8%) |  |
| Basal Cell Carcinoma \| Melanoma \| Prostate Cancer | 0 (0.0%) | 0 (0.0%) | 0 (0.0%) | 1 (1.2%) | 1 (0.5%) |  |
| Bladder Cancer | 0 (0.0%) | 0 (0.0%) | 0 (0.0%) | 6 (7.0%) | 6 (2.7%) |  |
| Breast Cancer | 56 (100.0%) | 0 (0.0%) | 0 (0.0%) | 0 (0.0%) | 56 (25.3%) |  |
| Breast Cancer \| Basal Cell Carcinoma | 0 (0.0%) | 0 (0.0%) | 0 (0.0%) | 2 (2.3%) | 2 (0.9%) |  |
| Breast Cancer \| Bone Cancer | 0 (0.0%) | 0 (0.0%) | 0 (0.0%) | 1 (1.2%) | 1 (0.5%) |  |
| Breast Cancer \| Melanoma | 0 (0.0%) | 0 (0.0%) | 0 (0.0%) | 1 (1.2%) | 1 (0.5%) |  |
| Breast Cancer \| Melanoma \| GIST | 0 (0.0%) | 0 (0.0%) | 0 (0.0%) | 1 (1.2%) | 1 (0.5%) |  |
| Breast Cancer \| Skin Cancer | 0 (0.0%) | 0 (0.0%) | 0 (0.0%) | 3 (3.5%) | 3 (1.4%) |  |
| Breast Cancer \| Squamous Cell Carcinoma | 0 (0.0%) | 0 (0.0%) | 0 (0.0%) | 1 (1.2%) | 1 (0.5%) |  |
| Breast Cancer \| Thyroid Cancer | 0 (0.0%) | 0 (0.0%) | 0 (0.0%) | 1 (1.2%) | 1 (0.5%) |  |
| Breast Cancer \| Uterine Cancer | 0 (0.0%) | 0 (0.0%) | 0 (0.0%) | 2 (2.3%) | 2 (0.9%) |  |
| Carcinoid - To General | 0 (0.0%) | 0 (0.0%) | 0 (0.0%) | 1 (1.2%) | 1 (0.5%) |  |
| Chronic Lymphocytic Leukemia | 0 (0.0%) | 1 (6.7%) | 0 (0.0%) | 0 (0.0%) | 1 (0.5%) |  |
| Chronic Myeloid Leukemia | 0 (0.0%) | 1 (6.7%) | 0 (0.0%) | 0 (0.0%) | 1 (0.5%) |  |
| Colon Cancer | 0 (0.0%) | 0 (0.0%) | 0 (0.0%) | 1 (1.2%) | 1 (0.5%) |  |
| Colon Cancer \| Skin Cancer | 0 (0.0%) | 0 (0.0%) | 0 (0.0%) | 1 (1.2%) | 1 (0.5%) |  |
| Colorectal Cancer \| Endometrial Cancer | 0 (0.0%) | 0 (0.0%) | 0 (0.0%) | 1 (1.2%) | 1 (0.5%) |  |
| Diffuse Large B-Cell Lymphoma | 0 (0.0%) | 1 (6.7%) | 0 (0.0%) | 0 (0.0%) | 1 (0.5%) |  |
| Endometrial Cancer | 0 (0.0%) | 0 (0.0%) | 0 (0.0%) | 3 (3.5%) | 3 (1.4%) |  |
| Fallopian Tube Cancer | 0 (0.0%) | 0 (0.0%) | 0 (0.0%) | 1 (1.2%) | 1 (0.5%) |  |
| Follicular Lymphoma | 0 (0.0%) | 2 (13.3%) | 0 (0.0%) | 0 (0.0%) | 2 (0.9%) |  |
| Hodgkin Lymphoma | 0 (0.0%) | 1 (6.7%) | 0 (0.0%) | 0 (0.0%) | 1 (0.5%) |  |
| Kidney Cancer | 0 (0.0%) | 0 (0.0%) | 0 (0.0%) | 1 (1.2%) | 1 (0.5%) |  |
| Large Granular Lymphocytic Leukemia | 0 (0.0%) | 1 (6.7%) | 0 (0.0%) | 0 (0.0%) | 1 (0.5%) |  |
| Leukemia | 0 (0.0%) | 1 (6.7%) | 0 (0.0%) | 0 (0.0%) | 1 (0.5%) |  |
| Liver Cancer \| Skin Cancer | 0 (0.0%) | 0 (0.0%) | 0 (0.0%) | 1 (1.2%) | 1 (0.5%) |  |
| Lung Cancer | 0 (0.0%) | 0 (0.0%) | 0 (0.0%) | 3 (3.5%) | 3 (1.4%) |  |
| Lymphoma | 0 (0.0%) | 2 (13.3%) | 0 (0.0%) | 0 (0.0%) | 2 (0.9%) |  |
| Lymphoplasmacytic Lymphoma | 0 (0.0%) | 1 (6.7%) | 0 (0.0%) | 0 (0.0%) | 1 (0.5%) |  |
| Medullary Thyroid Cancer | 0 (0.0%) | 0 (0.0%) | 0 (0.0%) | 2 (2.3%) | 2 (0.9%) |  |
| Melanoma | 0 (0.0%) | 0 (0.0%) | 0 (0.0%) | 3 (3.5%) | 3 (1.4%) |  |
| Melanoma \| Basal Cell Carcinoma \| Endometrial Cancer | 0 (0.0%) | 0 (0.0%) | 0 (0.0%) | 1 (1.2%) | 1 (0.5%) |  |
| Melanoma \| Thyroid Cancer | 0 (0.0%) | 0 (0.0%) | 0 (0.0%) | 1 (1.2%) | 1 (0.5%) |  |
| Multiple Myeloma | 0 (0.0%) | 0 (0.0%) | 64 (100.0%) | 0 (0.0%) | 64 (29.0%) |  |
| Myeloma | 0 (0.0%) | 1 (6.7%) | 0 (0.0%) | 0 (0.0%) | 1 (0.5%) |  |
| Non-Hodgkin Lymphoma | 0 (0.0%) | 1 (6.7%) | 0 (0.0%) | 0 (0.0%) | 1 (0.5%) |  |
| Oral Cancer | 0 (0.0%) | 0 (0.0%) | 0 (0.0%) | 1 (1.2%) | 1 (0.5%) |  |
| Oral Cancer \| Breast Cancer | 0 (0.0%) | 0 (0.0%) | 0 (0.0%) | 1 (1.2%) | 1 (0.5%) |  |
| Ovarian Cancer | 0 (0.0%) | 0 (0.0%) | 0 (0.0%) | 4 (4.7%) | 4 (1.8%) |  |
| Ovarian Cancer \| Breast Cancer | 0 (0.0%) | 0 (0.0%) | 0 (0.0%) | 1 (1.2%) | 1 (0.5%) |  |
| Ovarian Cancer \| Small Intestine Cancer \| Liver Cancer | 0 (0.0%) | 0 (0.0%) | 0 (0.0%) | 1 (1.2%) | 1 (0.5%) |  |
| Pancreatic Cancer | 0 (0.0%) | 0 (0.0%) | 0 (0.0%) | 1 (1.2%) | 1 (0.5%) |  |
| Parotid Cancer | 0 (0.0%) | 0 (0.0%) | 0 (0.0%) | 1 (1.2%) | 1 (0.5%) |  |
| Prostate Cancer | 0 (0.0%) | 0 (0.0%) | 0 (0.0%) | 10 (11.6%) | 10 (4.5%) |  |
| Prostate Cancer \| Melanoma | 0 (0.0%) | 0 (0.0%) | 0 (0.0%) | 1 (1.2%) | 1 (0.5%) |  |
| Rectal Cancer | 0 (0.0%) | 0 (0.0%) | 0 (0.0%) | 2 (2.3%) | 2 (0.9%) |  |
| Skin Cancer | 0 (0.0%) | 0 (0.0%) | 0 (0.0%) | 5 (5.8%) | 5 (2.3%) |  |
| Skin Cancer \| Ovarian Cancer | 0 (0.0%) | 0 (0.0%) | 0 (0.0%) | 1 (1.2%) | 1 (0.5%) |  |
| Smoldering Multiple Myeloma | 0 (0.0%) | 1 (6.7%) | 0 (0.0%) | 0 (0.0%) | 1 (0.5%) |  |
| Soft Tissue Sarcoma | 0 (0.0%) | 0 (0.0%) | 0 (0.0%) | 1 (1.2%) | 1 (0.5%) |  |
| Squamous Cell Carcinoma | 0 (0.0%) | 0 (0.0%) | 0 (0.0%) | 2 (2.3%) | 2 (0.9%) |  |
| Testicular Cancer | 0 (0.0%) | 0 (0.0%) | 0 (0.0%) | 4 (4.7%) | 4 (1.8%) |  |
| Testicular Cancer \| Prostate Cancer | 0 (0.0%) | 0 (0.0%) | 0 (0.0%) | 1 (1.2%) | 1 (0.5%) |  |
| Thyroid Cancer | 0 (0.0%) | 0 (0.0%) | 0 (0.0%) | 2 (2.3%) | 2 (0.9%) |  |
| Uterine Cancer | 0 (0.0%) | 0 (0.0%) | 0 (0.0%) | 1 (1.2%) | 1 (0.5%) |  |
| Vaginal Cancer | 0 (0.0%) | 0 (0.0%) | 0 (0.0%) | 1 (1.2%) | 1 (0.5%) |  |

## Supplementary Table 2. Cancer Treatments

| **Cancer Group** |  | **2 doses (N=76)** | **3 doses (N=220)** | **4 doses (N=157)** | **5 doses (N=242)** |
| --- | --- | --- | --- | --- | --- |
| Breast Cancer | **Cured** |  |  |  |  |
|  | No | 0 | 3 (6.8%) | 0 (0.0%) | 5 (6.2%) |
|  | Not Reported | 0 | 5 (11.4%) | 0 (0.0%) | 0 (0.0%) |
|  | Unknown | 0 | 9 (20.5%) | 25 (86.2%) | 71 (87.7%) |
|  | Yes | 0 | 27 (61.4%) | 4 (13.8%) | 5 (6.2%) |
|  | **In_Remission** |  |  |  |  |
|  | No | 0 | 3 (6.8%) | 0 (0.0%) | 5 (6.2%) |
|  | Not Reported | 0 | 5 (11.4%) | 0 (0.0%) | 0 (0.0%) |
|  | Unknown | 0 | 9 (20.5%) | 25 (86.2%) | 71 (87.7%) |
|  | Yes | 0 | 27 (61.4%) | 4 (13.8%) | 5 (6.2%) |
|  | **In_Unspecified_Therapy** |  |  |  |  |
|  | No | 0 | 23 (52.3%) | 2 (6.9%) | 7 (8.6%) |
|  | Not Reported | 0 | 14 (31.8%) | 25 (86.2%) | 71 (87.7%) |
|  | Yes | 0 | 7 (15.9%) | 2 (6.9%) | 3 (3.7%) |
|  | **Chemotherapy** |  |  |  |  |
|  | No | 0 | 35 (79.5%) | 25 (86.2%) | 59 (72.8%) |
|  | Not Reported | 0 | 5 (11.4%) | 0 (0.0%) | 0 (0.0%) |
|  | Yes | 0 | 4 (9.1%) | 4 (13.8%) | 22 (27.2%) |
|  | **Radiation Therapy** |  |  |  |  |
|  | No | 0 | 27 (61.4%) | 21 (72.4%) | 52 (64.2%) |
|  | Not Reported | 0 | 5 (11.4%) | 0 (0.0%) | 0 (0.0%) |
|  | Yes | 0 | 12 (27.3%) | 8 (27.6%) | 29 (35.8%) |
|  | **Surgery** |  |  |  |  |
|  | No | 0 | 0 (0.0%) | 0 (0.0%) | 0 (0.0%) |
|  | Not Reported | 0 | 37 (84.1%) | 27 (93.1%) | 74 (91.4%) |
|  | Yes | 0 | 7 (15.9%) | 2 (6.9%) | 7 (8.6%) |
| Non-MM Hematological | **Cured** |  |  |  |  |
|  | No | 0 | 2 (16.7%) | 4 (18.2%) | 0 (0.0%) |
|  | Not Reported | 0 | 0 (0.0%) | 0 (0.0%) | 0 (0.0%) |
|  | Unknown | 0 | 0 (0.0%) | 8 (36.4%) | 8 (66.7%) |
|  | Yes | 0 | 10 (83.3%) | 10 (45.5%) | 4 (33.3%) |
|  | **In_Remission** |  |  |  |  |
|  | No | 0 | 2 (16.7%) | 4 (18.2%) | 0 (0.0%) |
|  | Not Reported | 0 | 0 (0.0%) | 0 (0.0%) | 0 (0.0%) |
|  | Unknown | 0 | 0 (0.0%) | 8 (36.4%) | 8 (66.7%) |
|  | Yes | 0 | 10 (83.3%) | 10 (45.5%) | 4 (33.3%) |
|  | **In_Unspecified_Therapy** |  |  |  |  |
|  | No | 0 | 8 (66.7%) | 9 (40.9%) | 4 (33.3%) |
|  | Not Reported | 0 | 0 (0.0%) | 10 (45.5%) | 8 (66.7%) |
|  | Yes | 0 | 4 (33.3%) | 3 (13.6%) | 0 (0.0%) |
|  | **Chemotherapy** |  |  |  |  |
|  | No | 0 | 10 (83.3%) | 10 (45.5%) | 8 (66.7%) |
|  | Not Reported | 0 | 0 (0.0%) | 0 (0.0%) | 0 (0.0%) |
|  | Yes | 0 | 2 (16.7%) | 12 (54.5%) | 4 (33.3%) |
|  | **Radiation Therapy** |  |  |  |  |
|  | No | 0 | 10 (83.3%) | 20 (90.9%) | 8 (66.7%) |
|  | Not Reported | 0 | 0 (0.0%) | 2 (9.1%) | 0 (0.0%) |
|  | Yes | 0 | 2 (16.7%) | 0 (0.0%) | 4 (33.3%) |
|  | **Surgery** |  |  |  |  |
|  | No | 0 | 2 (16.7%) | 8 (36.4%) | 2 (16.7%) |
|  | Not Reported | 0 | 8 (66.7%) | 14 (63.6%) | 8 (66.7%) |
|  | Yes | 0 | 2 (16.7%) | 0 (0.0%) | 2 (16.7%) |
| Multiple Myeloma | **Cured** |  |  |  |  |
|  | No | 76 (100.0%) | 120 (100.0%) | 43 (100.0%) | 6 (100.0%) |
|  | Not Reported | 0 (0.0%) | 0 (0.0%) | 0 (0.0%) | 0 (0.0%) |
|  | Unknown | 0 (0.0%) | 0 (0.0%) | 0 (0.0%) | 0 (0.0%) |
|  | Yes | 0 (0.0%) | 0 (0.0%) | 0 (0.0%) | 0 (0.0%) |
|  | **In_Remission** |  |  |  |  |
|  | No | 41 (53.9%) | 58 (48.3%) | 22 (51.2%) | 2 (33.3%) |
|  | Not Reported | 0 (0.0%) | 0 (0.0%) | 0 (0.0%) | 0 (0.0%) |
|  | Unknown | 0 (0.0%) | 0 (0.0%) | 0 (0.0%) | 0 (0.0%) |
|  | Yes | 35 (46.1%) | 62 (51.7%) | 21 (48.8%) | 4 (66.7%) |
|  | **In_Unspecified_Therapy** |  |  |  |  |
|  | No | 0 (0.0%) | 0 (0.0%) | 0 (0.0%) | 0 (0.0%) |
|  | Not Reported | 76 (100.0%) | 120 (100.0%) | 43 (100.0%) | 6 (100.0%) |
|  | Yes | 0 (0.0%) | 0 (0.0%) | 0 (0.0%) | 0 (0.0%) |
|  | **Chemotherapy** |  |  |  |  |
|  | No | 20 (26.3%) | 16 (13.3%) | 2 (4.7%) | 0 (0.0%) |
|  | Not Reported | 0 (0.0%) | 0 (0.0%) | 0 (0.0%) | 0 (0.0%) |
|  | Yes | 56 (73.7%) | 104 (86.7%) | 41 (95.3%) | 6 (100.0%) |
|  | **Radiation Therapy** |  |  |  |  |
|  | No | 0 (0.0%) | 0 (0.0%) | 0 (0.0%) | 0 (0.0%) |
|  | Not Reported | 76 (100.0%) | 120 (100.0%) | 43 (100.0%) | 6 (100.0%) |
|  | Yes | 0 (0.0%) | 0 (0.0%) | 0 (0.0%) | 0 (0.0%) |
|  | **Surgery** |  |  |  |  |
|  | No | 76 (100.0%) | 120 (100.0%) | 43 (100.0%) | 6 (100.0%) |
|  | Not Reported | 0 (0.0%) | 0 (0.0%) | 0 (0.0%) | 0 (0.0%) |
|  | Yes | 0 (0.0%) | 0 (0.0%) | 0 (0.0%) | 0 (0.0%) |
| Other Solid Cancer | **Cured** |  |  |  |  |
|  | No | 0 | 4 (9.1%) | 0 (0.0%) | 0 (0.0%) |
|  | Not Reported | 0 | 21 (47.7%) | 0 (0.0%) | 3 (2.3%) |
|  | Unknown | 0 | 3 (6.8%) | 51 (83.6%) | 114 (86.4%) |
|  | Yes | 0 | 16 (36.4%) | 10 (16.4%) | 15 (11.4%) |
|  | **In_Remission** |  |  |  |  |
|  | No | 0 | 4 (9.1%) | 0 (0.0%) | 0 (0.0%) |
|  | Not Reported | 0 | 21 (47.7%) | 0 (0.0%) | 3 (2.3%) |
|  | Unknown | 0 | 3 (6.8%) | 51 (83.6%) | 114 (86.4%) |
|  | Yes | 0 | 16 (36.4%) | 10 (16.4%) | 15 (11.4%) |
|  | **In_Unspecified_Therapy** |  |  |  |  |
|  | No | 0 | 20 (45.5%) | 10 (16.4%) | 15 (11.4%) |
|  | Not Reported | 0 | 24 (54.5%) | 51 (83.6%) | 117 (88.6%) |
|  | Yes | 0 | 0 (0.0%) | 0 (0.0%) | 0 (0.0%) |
|  | **Chemotherapy** |  |  |  |  |
|  | No | 0 | 18 (40.9%) | 38 (62.3%) | 85 (64.4%) |
|  | Not Reported | 0 | 21 (47.7%) | 0 (0.0%) | 3 (2.3%) |
|  | Yes | 0 | 5 (11.4%) | 23 (37.7%) | 44 (33.3%) |
|  | **Radiation Therapy** |  |  |  |  |
|  | No | 0 | 23 (52.3%) | 39 (63.9%) | 102 (77.3%) |
|  | Not Reported | 0 | 21 (47.7%) | 0 (0.0%) | 3 (2.3%) |
|  | Yes | 0 | 0 (0.0%) | 22 (36.1%) | 27 (20.5%) |
|  | **Surgery** |  |  |  |  |
|  | No | 0 | 0 (0.0%) | 0 (0.0%) | 0 (0.0%) |
|  | Not Reported | 0 | 40 (90.9%) | 54 (88.5%) | 120 (90.9%) |
|  | Yes | 0 | 4 (9.1%) | 7 (11.5%) | 12 (9.1%) |

## Supplementary Table 3. Anti-Spike IgG level in each cohort

|  |  | Group | | N | Geometric mean concentration (BAU/mL) | 95% CI | p-value when compared to Healthy * | Matched Healthy Control - N | Matched Healthy Control - Geometric mean concentration (BAU/mL) | Matched Healthy Control - 95%CI | p-value when compared to Matched Healthy Control* |
| --- | --- | --- | --- | --- | --- | --- | --- | --- | --- | --- | --- |
| 1 month | 2 doses | Healthy |  | 31 | 2744 | 2110-3570 | -- | -- | -- | -- | -- |
|  |  | Hematological Cancer | Multiple myeloma | 24 | 736 | 290-1869 | 0.01875 | 19 | 2744.7 | 1958.2-3847.1 | 0.1041 |
|  |  |  | Non-MM hematological cancer | 0 | -- | -- | -- | -- | -- | -- | -- |
|  |  |  | Total | 24 | 736 | 290-1869 | 0.01875 | 19 | 2744.7 | 1958.2-3847.1 | 0.1041 |
|  |  | Solid Cancer | Breast cancer | 0 | -- | -- | -- | -- | -- | -- | -- |
|  |  |  | Other solid cancer | 0 | -- | -- | -- | -- | -- | -- | -- |
|  |  |  | Total | 0 | -- | -- | -- | -- | -- | -- | -- |
|  | 3 doses | Healthy |  | 244 | 7685 | 7014-8420 | -- | -- | -- | -- | -- |
|  |  | Hematological Cancer | Multiple myeloma | 34 | 1606 | 753-3423 | 0.00002032 | 24 | 7466 | 5776.8-9649.1 | 0.01127 |
|  |  |  | Non-MM hematological cancer | 4 | 10371 | 2358-45613 | 0.5812 | 3 | 7156.8 | 3526.5-14524.1 | 1 |
|  |  |  | Total | 38 | 1954 | 965-3956 | 0.0001264 | 27 | 7431 | 5914.7-9336.1 | 0.01821 |
|  |  | Solid Cancer | Breast cancer | 13 | 11309 | 7000-18271 | 0.08946 | 12 | 10496.2 | 7630.7-14437.8 | 0.7553 |
|  |  |  | Other solid cancer | 13 | 7215 | 5705-9125 | 0.7696 | 10 | 7994.4 | 4694.6-13613.8 | 0.8094 |
|  |  |  | Total | 26 | 9033 | 6937-11763 | 0.3329 | 22 | 9247.4 | 9035.1-12226.5 | 0.9373 |
|  | 4 doses | Healthy |  | 82 | 8805 | 7325-10585 | -- | -- | -- | -- | -- |
|  |  | Hematological Cancer | Multiple myeloma | 13 | 2215 | 981-4999 | 0.0006097 | 8 | 8639.3 | 4594-16246.8 | 0.04298 |
|  |  |  | Non-MM hematological cancer | 10 | 6694 | 2113-21204 | 0.8557 | 7 | 9456.8 | 4175.5-21418.0 | 0.6943 |
|  |  |  | Total | 23 | 3582 | 1842-6968 | 0.01022 | 14 | 8294.2 | 5386.2-12772.4 | 0.6084 |
|  |  | Solid Cancer | Breast cancer | 14 | 12207 | 7858-18961 | 0.1419 | 12 | 10680.9 | 6803.9-16767.1 | 0.5512 |
|  |  |  | Other solid cancer | 27 | 11380 | 7865-16466 | 0.1572 | 17 | 10646.4 | 7407.6-15301.4 | 0.4746 |
|  |  |  | Total | 41 | 11656 | 8857-15340 | 0.06534 | 29 | 10660.7 | 8185.8-13883.9 | 0.3131 |
|  | 5 doses | Healthy |  | 66 | 10352 | 8517-12583 | -- | -- | -- | -- | -- |
|  |  | Hematological Cancer | Multiple myeloma | 2 | 12662 | 85-1888551 | 0.7031 | 1 | 16939 | NA | 1 |
|  |  |  | Non-MM hematological cancer | 6 | 5383 | 517-56089 | 0.9756 | 4 | 11309.1 | 5458.2-23431.9 | 0.6857 |
|  |  |  | Total | 8 | 6666 | 1317-33750 | 0.8824 | 5 | 12260.9 | 7137.8-21060.9 | 0.5476 |
|  |  | Solid Cancer | Breast cancer | 37 | 11953 | 8685-16451 | 0.3998 | 25 | 11826.1 | 8656.5-16156.3 | 0.9132 |
|  |  |  | Other solid cancer | 57 | 10379 | 7875-13678 | 0.6645 | 29 | 9864.1 | 7242.9-13434.0 | 0.5696 |
|  |  |  | Total | 94 | 10972 | 8929-13482 | 0.4699 | 53 | 10804.5 | 8689.8-13433.9 | 0.6594 |
| 6 month | 2 doses | Healthy |  | 31 | 850 | 501-1441 | -- | -- | -- | -- | -- |
|  |  | Hematological Cancer | Multiple myeloma | 24 | 231 | 116-460 | 0.01353 | 19 | 484.6 | 267.2-878.8 | 0.4646 |
|  |  |  | Non-MM hematological cancer | 0 | -- | -- | -- | -- | -- | -- | -- |
|  |  |  | Total | 24 | 231 | 116-460 | 0.01353 | 19 | 484.6 | 267.2-878.8 | 0.4646 |
|  |  | Solid Cancer | Breast cancer | 0 | -- | -- | -- | -- | -- | -- | -- |
|  |  |  | Other solid cancer | 0 | -- | -- | -- | -- | -- | -- | -- |
|  |  |  | Total | 0 | -- | -- | -- | -- | -- | -- | -- |
|  | 3 doses | Healthy |  | 240 | 3277 | 2916-3683 | -- | -- | -- | -- | -- |
|  |  | Hematological Cancer | Multiple myeloma | 34 | 2192 | 1307-3675 | 0.08941 | 24 | 3060 | 1956.9-4784.8 | 0.6371 |
|  |  |  | Non-MM hematological cancer | 4 | 5405 | 278-105073 | 0.5557 | 3 | 4901.7 | 540.2-44475.9 | 0.7 |
|  |  |  | Total | 38 | 2410 | 1461-3975 | 0.1571 | 27 | 3224.4 | 2139.6-4859.3 | 0.5254 |
|  |  | Solid Cancer | Breast cancer | 13 | 5649 | 3510-9092 | 0.03648 | 12 | 2969.8 | 1852.0-4762.1 | 0.06836 |
|  |  |  | Other solid cancer | 13 | 2240 | 1139-4406 | 0.1387 | 10 | 1980.5 | 899.4-4360.9 | 0.7045 |
|  |  |  | Total | 26 | 3557 | 2319-5456 | 0.6745 | 22 | 2470.3 | 1639.9-3721.0 | 0.1785 |
|  | 4 doses | Healthy |  | 81 | 5385 | 4583-6328 | -- | -- | -- | -- | -- |
|  |  | Hematological Cancer | Multiple myeloma | 13 | 2192 | 934-5143 | 0.004717 | 8 | 4695.8 | 2534.1-8701.5 | 0.6454 |
|  |  |  | Non-MM hematological cancer | 10 | 1811 | 556-5897 | 0.01922 | 7 | 5693.1 | 3689.3-8785.2 | 0.3969 |
|  |  |  | Total | 23 | 2017 | 1061-3837 | 0.000521 | 14 | 5108.8 | 3538.1-7376.7 | 0.1934 |
|  |  | Solid Cancer | Breast cancer | 14 | 5860 | 4049-8480 | 0.8051 | 12 | 5582.3 | 3729.8-8354.8 | 0.9774 |
|  |  |  | Other solid cancer | 27 | 5282 | 3296-8465 | 0.5751 | 17 | 4896.3 | 3025.6-7923.6 | 0.3615 |
|  |  |  | Total | 41 | 5473 | 3955-7574 | 0.5767 | 29 | 5169.3 | 3792.1-7046.7 | 0.269 |
|  | 5 doses | Healthy |  | 65 | 5311 | 4159-6781 | -- | -- | -- | -- | -- |
|  |  | Hematological Cancer | Multiple myeloma | 2 | 4289 | 579-31746 | 0.5932 | 1 | 17744.7 | NA | 1 |
|  |  |  | Non-MM hematological cancer | 6 | 2205 | 232-20982 | 0.5838 | 4 | 3935.6 | 1817.1-8524.1 | 0.8857 |
|  |  |  | Total | 8 | 2604 | 558-12148 | 0.4636 | 5 | 5318.9 | 1984.4-14256.8 | 0.6905 |
|  |  | Solid Cancer | Breast cancer | 37 | 6126 | 4496-8348 | 0.3264 | 25 | 5573.6 | 3936.6-7891.2 | 0.706 |
|  |  |  | Other solid cancer | 57 | 5379 | 3999-7235 | 0.8979 | 29 | 5227.3 | 3396.1-8045.9 | 0.8426 |
|  |  |  | Total | 94 | 5661 | 4572-7010 | 0.5597 | 53 | 5447.1 | 4127.1-7189.1 | 0.9367 |
| *Differences assessed by non-parametric Wilcoxon rank sum test. | | | | | | | | | | | |

## Supplementary Table 4. Anti-Spike IgG Avidity in each cohort

|  |  | Group | | N | Geometric mean concentration (M) | 95% CI | p-value when compared to Healthy * | p-value when compared to Matched Healthy Control* |
| --- | --- | --- | --- | --- | --- | --- | --- | --- |
| 1 month | 2 doses | Healthy |  | 32 | 2.8 | 2.6-3.2 | -- | -- |
|  |  | Hematological Cancer | Multiple myeloma | 23 | 2.8 | 2.5-3.2 | 0.8914 | 0.6089 |
|  |  |  | Non-MM hematological cancer | 0 | -- | -- | -- | -- |
|  |  |  | Total | 23 | 2.8 | 2.5-3.2 | 0.8914 | 0.6089 |
|  |  | Solid Cancer | Breast cancer | 0 | -- | -- | -- | -- |
|  |  |  | Other solid cancer | 0 | -- | -- | -- | -- |
|  |  |  | Total | 0 | -- | -- | -- | -- |
|  | 3 doses | Healthy |  | 244 | 5.5 | 5.4-5.6 | -- | -- |
|  |  | Hematological Cancer | Multiple myeloma | 33 | 4.3 | 4.0-4.6 | 1.932E-11 | 0.00006315 |
|  |  |  | Non-MM hematological cancer | 4 | 5.4 | 4.0-7.2 | 0.6605 | 0.4 |
|  |  |  | Total | 37 | 4.4 | 4.1-4.7 | 1.28E-10 | 0.0003515 |
|  |  | Solid Cancer | Breast cancer | 13 | 5.3 | 4.9-5.8 | 0.5188 | 0.2657 |
|  |  |  | Other solid cancer | 13 | 5.0 | 4.7-5.4 | 0.007695 | 0.1971 |
|  |  |  | Total | 26 | 5.2 | 4.9-5.4 | 0.0223 | 0.05947 |
|  | 4 doses | Healthy |  | 82 | 5.8 | 5.6-5.9 | -- | -- |
|  |  | Hematological Cancer | Multiple myeloma | 13 | 4.7 | 4.0-5.6 | 0.006676 | 0.03792 |
|  |  |  | Non-MM hematological cancer | 10 | 4.8 | 4.2-5.4 | 0.0003761 | 0.09386 |
|  |  |  | Total | 23 | 4.7 | 4.3-5.2 | 0.00003456 | 0.004486 |
|  |  | Solid Cancer | Breast cancer | 14 | 5.8 | 5.4-6.2 | 0.7009 | 0.6297 |
|  |  |  | Other solid cancer | 27 | 5.8 | 5.4-6.1 | 0.8884 | 0.5657 |
|  |  |  | Total | 41 | 5.8 | 5.5-6.0 | 0.9295 | 0.408 |
|  | 5 doses | Healthy |  | 66 | 5.7 | 5.5-5.9 | -- | -- |
|  |  | Hematological Cancer | Multiple myeloma | 2 | 5.0 | 0.4-58.6 | 0.5492 | 1 |
|  |  |  | Non-MM hematological cancer | 6 | 5.1 | 3.9-6.7 | 0.6614 | 0.8857 |
|  |  |  | Total | 8 | 5.1 | 4.2-6.3 | 0.5027 | 0.8413 |
|  |  | Solid Cancer | Breast cancer | 37 | 5.7 | 5.5-5.9 | 0.8798 | 0.6326 |
|  |  |  | Other solid cancer | 57 | 5.7 | 5.5-5.9 | 0.7706 | 0.8903 |
|  |  |  | Total | 94 | 5.7 | 5.6-5.9 | 0.9034 | 0.7822 |
| 6 month | 2 doses | Healthy |  | 32 | 4.0 | 3.5-4.4 | -- | -- |
|  |  | Hematological Cancer | Multiple myeloma | 23 | 3.1 | 2.8-3.5 | 0.005181 | 0.03048 |
|  |  |  | Non-MM hematological cancer | 0 | -- | -- | -- | -- |
|  |  |  | Total | 23 | 3.1 | 2.8-3.5 | 0.005181 | 0.03048 |
|  |  | Solid Cancer | Breast cancer | 0 | -- | -- | -- | -- |
|  |  |  | Other solid cancer | 0 | -- | -- | -- | -- |
|  |  |  | Total | 0 | -- | -- | -- | -- |
|  | 3 doses | Healthy |  | 240 | 5.4 | 5.3-5.5 | -- | -- |
|  |  | Hematological Cancer | Multiple myeloma | 34 | 4.5 | 4.2-4.8 | 1.259E-07 | 0.002911 |
|  |  |  | Non-MM hematological cancer | 4 | 5.4 | 4.4-6.7 | 0.4036 | 1 |
|  |  |  | Total | 38 | 4.6 | 4.3-4.9 | 2.231E-07 | 0.002391 |
|  |  | Solid Cancer | Breast cancer | 13 | 5.5 | 5.1-6.0 | 0.3908 | 0.2913 |
|  |  |  | Other solid cancer | 13 | 5.4 | 4.8-6.1 | 0.6189 | 0.9725 |
|  |  |  | Total | 26 | 5.5 | 5.1-5.9 | 0.8038 | 0.4239 |
|  | 4 doses | Healthy |  | 81 | 5.9 | 5.7-6.0 | -- | -- |
|  |  | Hematological Cancer | Multiple myeloma | 13 | 4.8 | 4.2-5.5 | 0.005195 | 0.1949 |
|  |  |  | Non-MM hematological cancer | 10 | 4.7 | 4.0-5.4 | 0.000365 | 0.01399 |
|  |  |  | Total | 23 | 4.8 | 4.3-5.2 | 0.00002313 | 0.003321 |
|  |  | Solid Cancer | Breast cancer | 14 | 5.6 | 5.2-6.1 | 0.2337 | 0.8874 |
|  |  |  | Other solid cancer | 27 | 5.6 | 5.3-6.0 | 0.5517 | 0.8119 |
|  |  |  | Total | 41 | 5.6 | 5.3-5.9 | 0.2785 | 0.7774 |
|  | 5 doses | Healthy |  | 65 | 5.8 | 5.6-6.0 | -- | -- |
|  |  | Hematological Cancer | Multiple myeloma | 2 | 4.8 | 1.3-18.2 | 0.0872 | 1 |
|  |  |  | Non-MM hematological cancer | 6 | 5.1 | 3.7-6.8 | 0.4193 | 1 |
|  |  |  | Total | 8 | 5.0 | 4.1-6.2 | 0.1275 | 0.6905 |
|  |  | Solid Cancer | Breast cancer | 37 | 5.7 | 5.5-6.0 | 0.7991 | 0.706 |
|  |  |  | Other solid cancer | 57 | 5.7 | 5.5-6.0 | 0.984 | 0.7373 |
|  |  |  | Total | 94 | 5.7 | 5.5-5.9 | 0.8838 | 0.9978 |
| *Differences assessed by non-parametric Wilcoxon rank sum test. | | | | | | | | |

## Supplementary Table 5. Anti-NC IgG Percent of Positive in each cohort

|  |  | **Group** | | **N** | **Positive** | **Negative** | **p-value*** |
| --- | --- | --- | --- | --- | --- | --- | --- |
| 1 month | 2 doses | Healthy |  | 31 | 2 (6.5%) | 29 (93.5%) | -- |
|  |  | Hematological Cancer | Multiple myeloma | 24 | 8 (33.3%) | 16 (66.7%) | 0.02704 |
|  |  |  | Non-MM hematological cancer | 0 | -- | -- | -- |
|  |  |  | Total | 24 | 8 (33.3%) | 16 (66.7%) | 0.02704 |
|  |  | Solid Cancer | Breast cancer | 0 | -- | -- | -- |
|  |  |  | Other solid cancer | 0 | -- | -- | -- |
|  |  |  | Total | 0 | -- | -- | -- |
|  | 3 doses | Healthy |  | 244 | 46 (18.9%) | 198 (81.1%) | -- |
|  |  | Hematological Cancer | Multiple myeloma | 34 | 5 (14.7%) | 29 (85.3%) | 0.7273 |
|  |  |  | Non-MM hematological cancer | 4 | 0 (0.0%) | 4 (100.0%) | 0.7537 |
|  |  |  | Total | 38 | 5 (13.2%) | 33 (86.8%) | 0.5341 |
|  |  | Solid Cancer | Breast cancer | 13 | 2 (15.4%) | 11 (84.6%) | 1 |
|  |  |  | Other solid cancer | 13 | 2 (15.4%) | 11 (84.6%) | 1 |
|  |  |  | Total | 26 | 4 (15.4%) | 22 (84.6%) | 0.8672 |
|  | 4 doses | Healthy |  | 82 | 17 (20.7%) | 65 (79.3%) | -- |
|  |  | Hematological Cancer | Multiple myeloma | 13 | 3 (23.1%) | 10 (76.9%) | 1 |
|  |  |  | Non-MM hematological cancer | 10 | 1 (10.0%) | 9 (90.0%) | 0.6999 |
|  |  |  | Total | 23 | 4 (17.4%) | 19 (82.6%) | 0.953 |
|  |  | Solid Cancer | Breast cancer | 14 | 2 (14.3%) | 12 (85.7%) | 0.8442 |
|  |  |  | Other solid cancer | 27 | 5 (18.5%) | 22 (81.5%) | 1 |
|  |  |  | Total | 41 | 7 (17.1%) | 34 (82.9%) | 0.8093 |
|  | 5 doses | Healthy |  | 66 | 17 (25.8%) | 49 (74.2%) | -- |
|  |  | Hematological Cancer | Multiple myeloma | 2 | 1 (50.0%) | 1 (50.0%) | 1 |
|  |  |  | Non-MM hematological cancer | 6 | 2 (33.3%) | 4 (66.7%) | 1 |
|  |  |  | Total | 8 | 3 (37.5%) | 5 (62.5%) | 0.7758 |
|  |  | Solid Cancer | Breast cancer | 37 | 7 (18.9%) | 30 (81.1%) | 0.5859 |
|  |  |  | Other solid cancer | 57 | 6 (10.5%) | 51 (89.5%) | 0.05379 |
|  |  |  | Total | 94 | 13 (13.8%) | 81 (86.2%) | 0.08966 |
| 6 months | 2 doses | Healthy |  | 31 | 6 (19.4%) | 25 (80.6%) | -- |
|  |  | Hematological Cancer | Multiple myeloma | 24 | 5 (20.8%) | 19 (79.2%) | 1 |
|  |  |  | Non-MM hematological cancer | 0 | -- | -- | -- |
|  |  |  | Total | 24 | 5 (20.8%) | 19 (79.2%) | 1 |
|  |  | Solid Cancer | Breast cancer | 0 | -- | -- | -- |
|  |  |  | Other solid cancer | 0 | -- | -- | -- |
|  |  |  | Total | 0 | -- | -- | -- |
|  | 3 doses | Healthy |  | 240 | 64 (26.7%) | 176 (73.3%) | -- |
|  |  | Hematological Cancer | Multiple myeloma | 34 | 7 (20.6%) | 27 (79.4%) | 0.5837 |
|  |  |  | Non-MM hematological cancer | 4 | 1 (25.0%) | 3 (75.0%) | 1 |
|  |  |  | Total | 38 | 8 (21.1%) | 30 (78.9%) | 0.5928 |
|  |  | Solid Cancer | Breast cancer | 13 | 3 (23.1%) | 10 (76.9%) | 1 |
|  |  |  | Other solid cancer | 13 | 5 (38.5%) | 8 (61.5%) | 0.5416 |
|  |  |  | Total | 26 | 8 (30.8%) | 18 (69.2%) | 0.8299 |
|  | 4 doses | Healthy |  | 81 | 18 (22.2%) | 63 (77.8%) | -- |
|  |  | Hematological Cancer | Multiple myeloma | 13 | 4 (30.8%) | 9 (69.2%) | 0.001649 |
|  |  |  | Non-MM hematological cancer | 10 | 0 (0.0%) | 10 (100.0%) | 0.2136 |
|  |  |  | Total | 23 | 9 (39.1%) | 14 (60.9%) | 0.1729 |
|  |  | Solid Cancer | Breast cancer | 14 | 1 (7.1%) | 13 (92.9%) | 0.3469 |
|  |  |  | Other solid cancer | 27 | 6 (22.2%) | 21 (77.8%) | 1 |
|  |  |  | Total | 41 | 7 (17.1%) | 34 (82.9%) | 0.6686 |
|  | 5 doses | Healthy |  | 65 | 15 (23.1%) | 50 (76.9%) | -- |
|  |  | Hematological Cancer | Multiple myeloma | 2 | 1 (50.0%) | 1 (50.0%) | 0.9699 |
|  |  |  | Non-MM hematological cancer | 6 | 1 (16.7%) | 5 (83.3%) | 1 |
|  |  |  | Total | 8 | 2 (25.0%) | 6 (75.0%) | 1 |
|  |  | Solid Cancer | Breast cancer | 37 | 8 (21.6%) | 29 (78.4%) | 1 |
|  |  |  | Other solid cancer | 57 | 11 (19.3%) | 46 (80.7%) | 0.7742 |
|  |  |  | Total | 94 | 19 (20.2%) | 75 (79.8%) | 0.8132 |
| *Differences assessed by Pearson's Chi-squared test. | | | | | | | |

## Supplementary Table 6. Percent Change of Anti-Spike IgG Avidity from 1 month to 6 months post vaccination within different age groups

|  | | | Younger (<65 years old) | | | | Older (≥ 65 years old | | | |
| --- | --- | --- | --- | --- | --- | --- | --- | --- | --- | --- |
|  | Group | | N | Median Percent Change (%) | Q1, Q3 | p-value when compared to Healthy | N | Median Percent Change (%) | Q1, Q3 | p-value when compared to Healthy |
| 2 doses | Healthy |  | 28 | 46.2 | 12.0, 82.9 | -- | 4.0 | 15.6 | 5.6, 28.9 | -- |
|  | Hematological Cancer | Multiple myeloma | 10 | 10.2 | 1.5, 35.9 | 0.03679 | 12.0 | 9.1 | -0.1, 44.7 | 0.8615 |
|  |  | Non-MM hematological cancer | -- | -- | -- | -- | -- | -- | -- | -- |
|  |  | Total | 10.0 | 10.2 | 1.5, 35.9 | 0.03679 | 12.0 | 9.1 | -0.1, 44.7 | 0.8615 |
|  | Solid Cancer | Breast cancer | -- | -- | -- | -- | -- | -- | -- | -- |
|  |  | Other solid cancer | -- | -- | -- | -- | -- | -- | -- | -- |
|  |  | Total | -- | -- | -- | -- | -- | -- | -- | -- |
| 3 doses | Healthy |  | 207.0 | -1.5 | -10.2, 7.5 | -- | 22.0 | -0.5 | -7.9, 8.7 | -- |
|  | Hematological Cancer | Multiple myeloma | 14.0 | -5.7 | -8.9, 9.3 | 0.7087 | 19.0 | 5.0 | -5.8, 21.6 | 0.2931 |
|  |  | Non-MM hematological cancer | 3.0 | -3.2 | -3.3, -2.0 | 0.901 | 1.0 | 12.0 | 12, 12 | 0.4348 |
|  |  | Total | 17.0 | -3.4 | -6.6, -0.8 | 0.6971 | 20.0 | 5.2 | -5.8, 20.1 | 0.2343 |
|  | Solid Cancer | Breast cancer | 9.0 | 4.6 | -6.5, 9.7 | 0.4587 | 4.0 | 10.7 | -7.5, 27.5 | 0.4706 |
|  |  | Other solid cancer | 7.0 | 10.4 | -2.8, 15.4 | 0.1607 | 6.0 | 8.7 | -12.6, 32.3 | 0.6039 |
|  |  | Total | 16.0 | 5.1 | -7.2, 13.9 | 0.1449 | 10.0 | 10.7 | -12.6, 31.2 | 0.411 |
| 4 doses | Healthy |  | 65.0 | 0.0 | -4.4, 7.4 | -- | 16.0 | 2.9 | -8.1, 13 | -- |
|  | Hematological Cancer | Multiple myeloma | 4.0 | 8.2 | -6.4, 1.6 | 0.6532 | 9.0 | -0.5 | -5, 10.5 | 0.846 |
|  |  | Non-MM hematological cancer | 7.0 | 0.0 | -6.6, 1.6 | 0.4819 | 3.0 | -1.6 | -6.5, 2.2 | 0.5593 |
|  |  | Total | 11.0 | 0.0 | -6.6, 9.7 | 0.7791 | 12.0 | -1.1 | -6.5, 7.7 | 0.6642 |
|  | Solid Cancer | Breast cancer | 11.0 | -6.7 | -11.6, 6.3 | 0.2318 | 3.0 | -3.2 | -4.8, -1.1 | 0.7926 |
|  |  | Other solid cancer | 15.0 | 0.6 | -5.7, 10.0 | 0.8533 | 12.0 | -7.2 | -10.1, -3.3 | 0.1457 |
|  |  | Total | 26.0 | -0.2 | -9.3, 10.3 | 0.565 | 15.0 | -6.5 | -8.8, -2.8 | 0.1751 |
| 5 doses | Healthy |  | 34.0 | -37.0 | -6.9, 9.2 | -- | 31.0 | -0.1 | -3, 4.7 | -- |
|  | Hematological Cancer | Multiple myeloma | 1.0 | 4.6 | 4.6, 4.6 | 0.8 | 1.0 | -12.4 | -12.4, -12.4 | 0.1875 |
|  |  | Non-MM hematological cancer | 3.0 | 1.6 | -1.1, 1.6 | 1 | 3.0 | 0.3 | -4.2, 0.6 | 0.5241 |
|  |  | Total | 4.0 | 1.6 | 0.3, 2.4 | 0.8725 | 4.0 | -4.2 | -9.6, 0.5 | 0.2129 |
|  | Solid Cancer | Breast cancer | 15.0 | -1.7 | -6.1, 2 | 0.5549 | 22.0 | 1.5 | -3.5, 7 | 0.9074 |
|  |  | Other solid cancer | 17.0 | -0.5 | -2, 9.6 | 0.4697 | 40.0 | -1.7 | -6.1, 5.3 | 0.3015 |
|  |  | Total | 32.0 | -1.4 | -4.2, 5.3 | 0.9137 | 62.0 | -0.5 | -6, 5.8 | 0.4316 |

## Supplementary Table 7. Percent Change of Anti-Spike IgG Avidity from 1 month to 6 months post vaccination within different sex groups

|  | | | Female | | | | Male | | | |
| --- | --- | --- | --- | --- | --- | --- | --- | --- | --- | --- |
|  | Group | | N | Median Percent Change (%) | Q1, Q3 | p-value when compared to Healthy * | N | Median Percent Change (%) | Q1, Q3 | p-value when compared to Healthy * |
| 2 doses | Healthy |  | 18 | 58.8 | 13.0, 100.9 | -- | 14 | 35.6 | 10.9, 48.3 | -- |
|  | Hematological Cancer | Multiple myeloma | 13 | 4.7 | -3.5, 46.2 | 0.0247 | 9 | 15.1 | 5.2, 30.4 | 0.1794 |
|  |  | Non-MM hematological cancer | -- | -- | -- | -- | -- | -- | -- | -- |
|  |  | Total | 13 | 4.7 | -3.5, 46.2 | 0.0247 | 9 | 15.1 | 5.2, 30.4 | 0.1794 |
|  | Solid Cancer | Breast cancer | -- | -- | -- | -- | -- | -- | -- | -- |
|  |  | Other solid cancer | -- | -- | -- | -- | -- | -- | -- | -- |
|  |  | Total | -- | -- | -- | -- | -- | -- | -- | -- |
| 3 doses | Healthy |  | 178 | -1.7 | -10.4, 7.7 | -- | 51 | 0.4 | -8.2, 8.2 | -- |
|  | Hematological Cancer | Multiple myeloma | 20 | 3.7 | -4.2, 22.5 | 0.04751 | 13 | -6.1 | -12.4, 6.3 | 0.3675 |
|  |  | Non-MM hematological cancer | 3 | -0.8 | -2, 5.6 | 0.4874 | 1 | -3.3 | -3.3, -3.3 | 0.739 |
|  |  | Total | 23 | 2.5 | -3.5, 17.7 | 0.03807 | 14 | -6 | -11.6, 3.9 | 0.3424 |
|  | Solid Cancer | Breast cancer | 13 | 4.6 | -6.5, 13.7 | 0.3273 | -- | -- | -- | -- |
|  |  | Other solid cancer | 9 | 4 | -11.9, 16.2 | 0.6427 | 4 | 27.4 | 7.7, 42.9 | 0.08293 |
|  |  | Total | 22 | 4.3 | -11.3, 15.6 | 0.3054 | 4 | 27.4 | 7.7, 42.9 | 0.08293 |
| 4 doses | Healthy |  | 59 | 0 | -4.5, 7.7 | -- | 22 | -0.1 | -7.2, 12.3 | -- |
|  | Hematological Cancer | Multiple myeloma | 6 | 9.6 | -3.6, 21.6 | 0.3025 | 7 | -5 | -8.5, 8.6 | 0.5001 |
|  |  | Non-MM hematological cancer | 3 | -6.3 | -12.4, -0.1 | 0.2509 | 7 | 0 | -4.3, 1.6 | 0.8227 |
|  |  | Total | 9 | -0.5 | -6.3, 19.8 | 0.8564 | 14 | -0.8 | -6.7, 5.9 | 0.5531 |
|  | Solid Cancer | Breast cancer | 14 | -4.8 | -10.8, 1.4 | 0.1675 | -- | -- | -- | -- |
|  |  | Other solid cancer | 17 | -3.6 | -9.3, 3.7 | 0.1482 | 10 | -3 | -6.8, 10.8 | 0.7643 |
|  |  | Total | 31 | -3.6 | -10.4, 2.8 | 0.06791 | 10 | -3 | -6.8, 10.8 | 0.7643 |
| 5 doses | Healthy |  | 42 | -0.9 | -6.2, 7.8 | -- | 23 | 2.7 | -3, 7.2 | -- |
|  | Hematological Cancer | Multiple myeloma | 2 | -3.9 | -8.1, 0.4 | 0.6469 | 0 | -- | -- | -- |
|  |  | Non-MM hematological cancer | 5 | 0.3 | -3.8, 0.9 | 0.7002 | 1 | 1.7 | 1.7, 1.7 | 1 |
|  |  | Total | 7 | 0.3 | -6.2, 1.3 | 0.5659 | 1 | 1.7 | 1.7, 1.7 | 1 |
|  | Solid Cancer | Breast cancer | 37 | -1.3 | -5.4, 4.8 | 0.826 | -- | -- | -- | -- |
|  |  | Other solid cancer | 32 | -3.6 | -7.5, 4.7 | 0.205 | 25 | 3.4 | -2.3, 6.8 | 0.6527 |
|  |  | Total | 69 | -1.7 | -6.4, 4.8 | 0.3963 | 25 | 3.4 | -2.3, 6.8 | 0.6527 |

# Supplementary Figures

Supplementary Figure 1


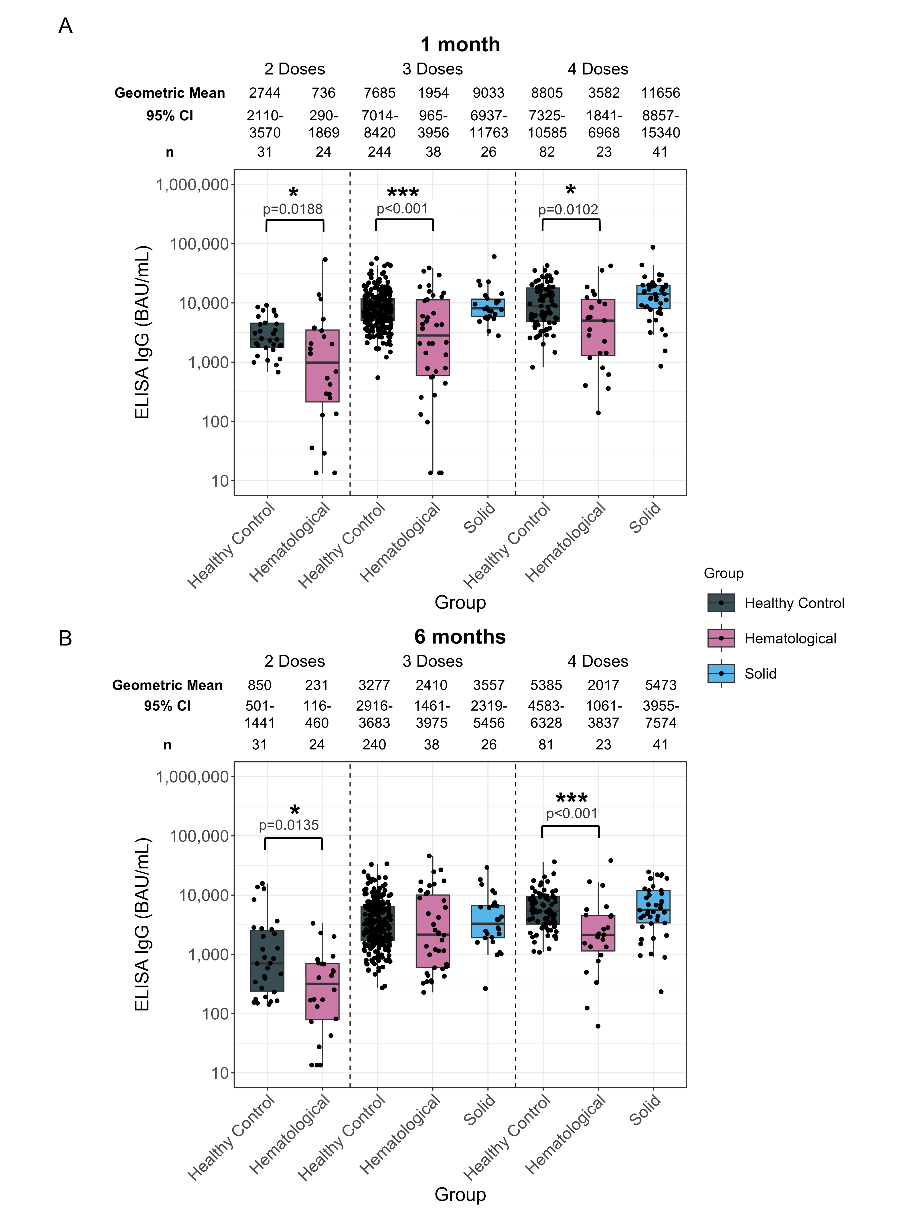


Supplementary Figure 2


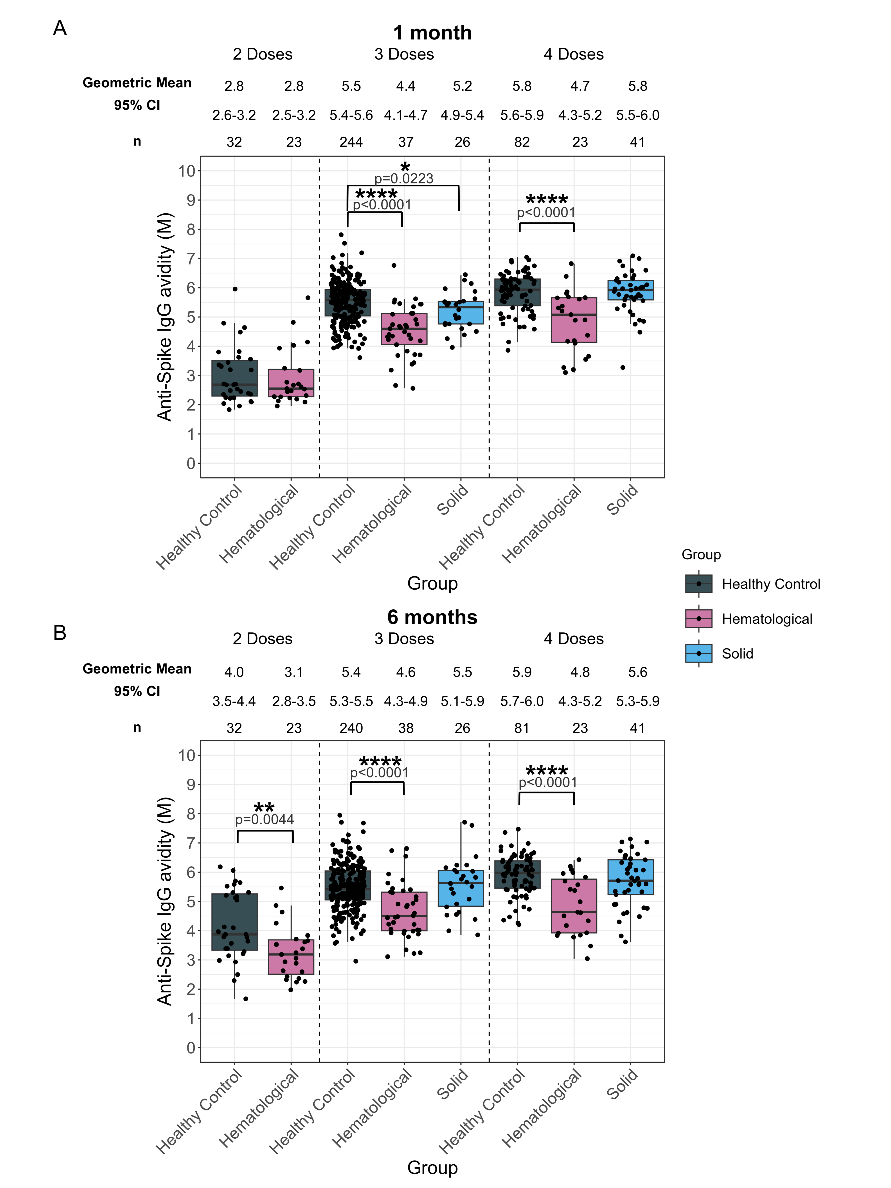


Supplementary Figure 3


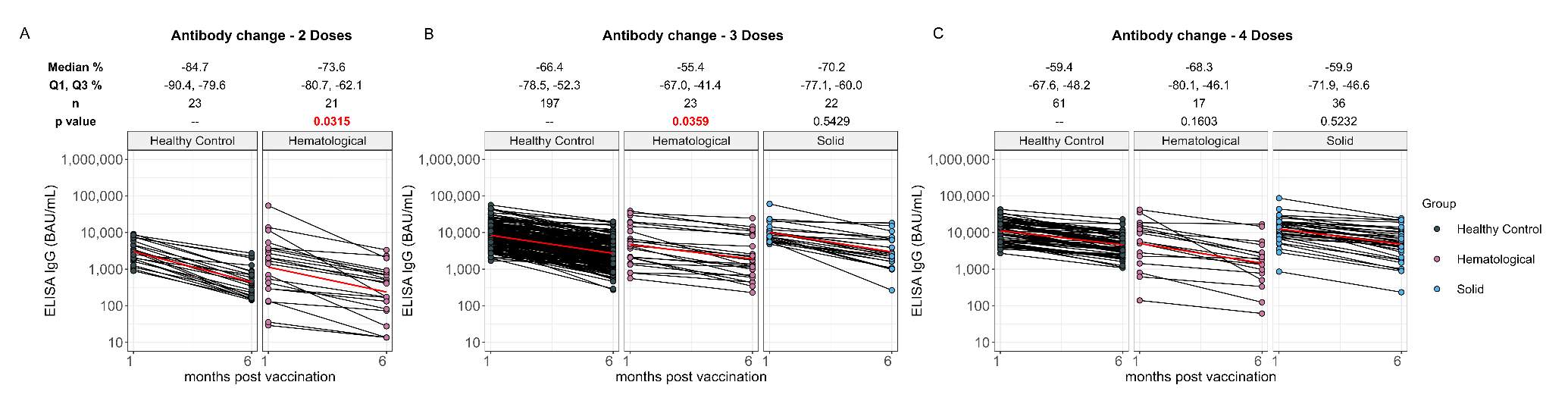


Supplementary Figure 4


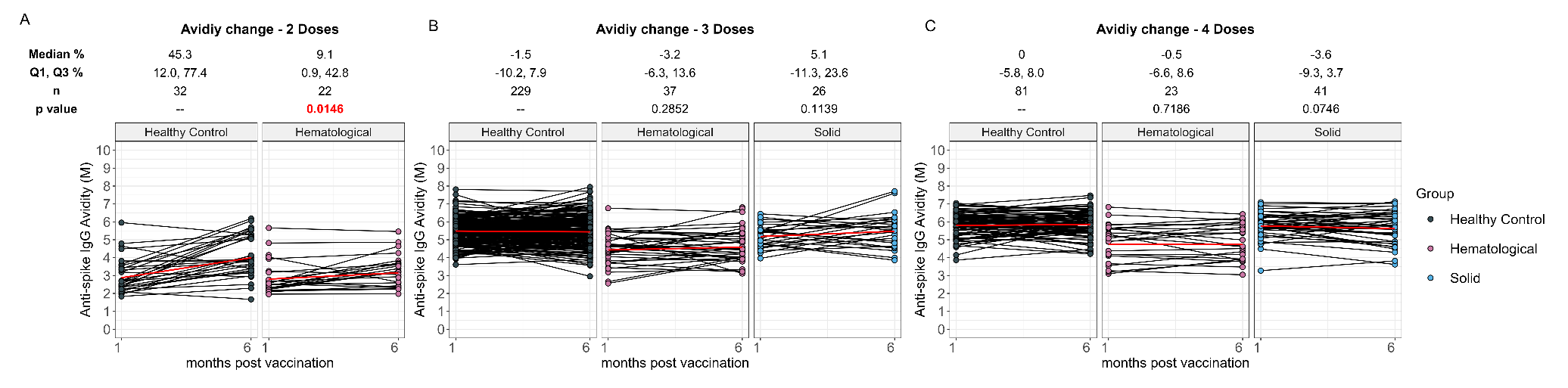


Supplementary Figure 5


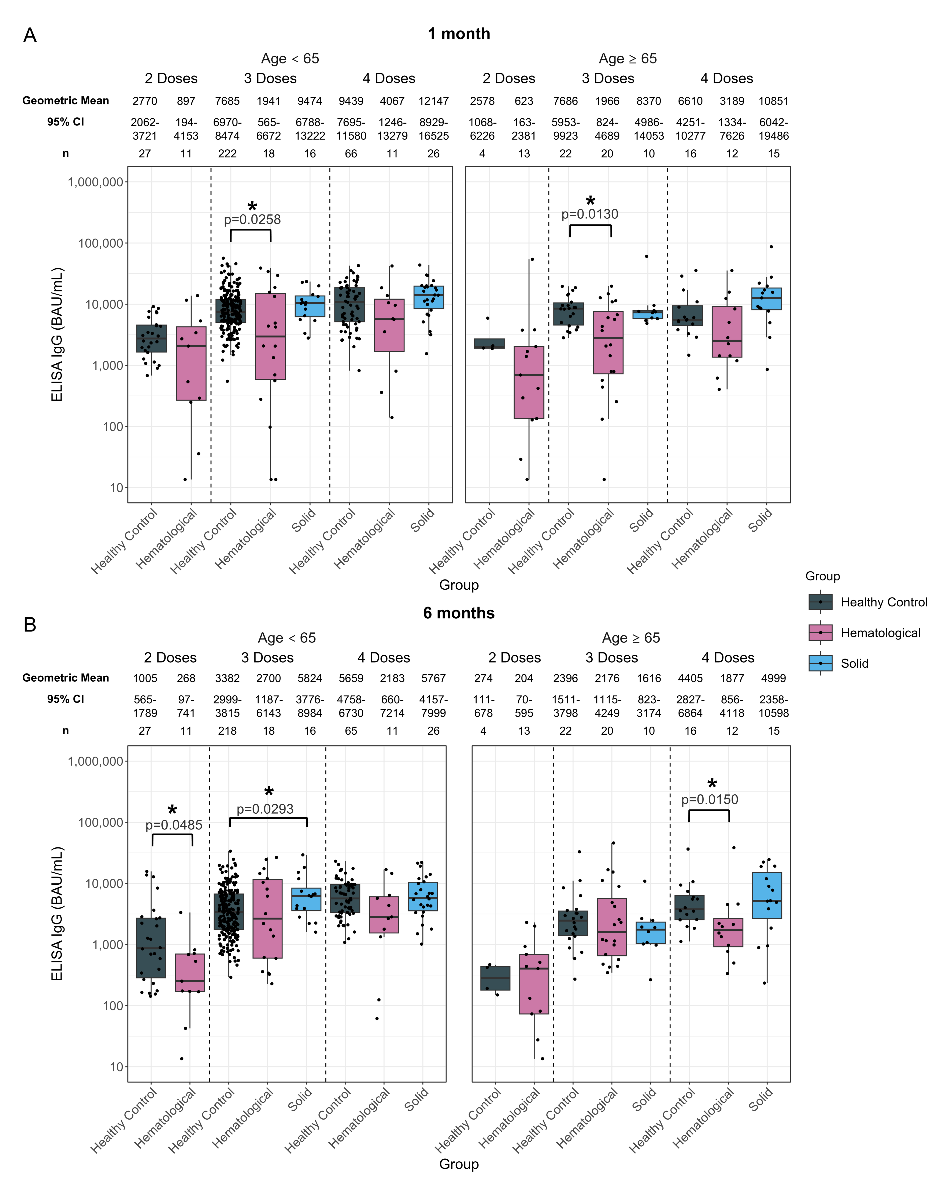


Supplementary Figure 6


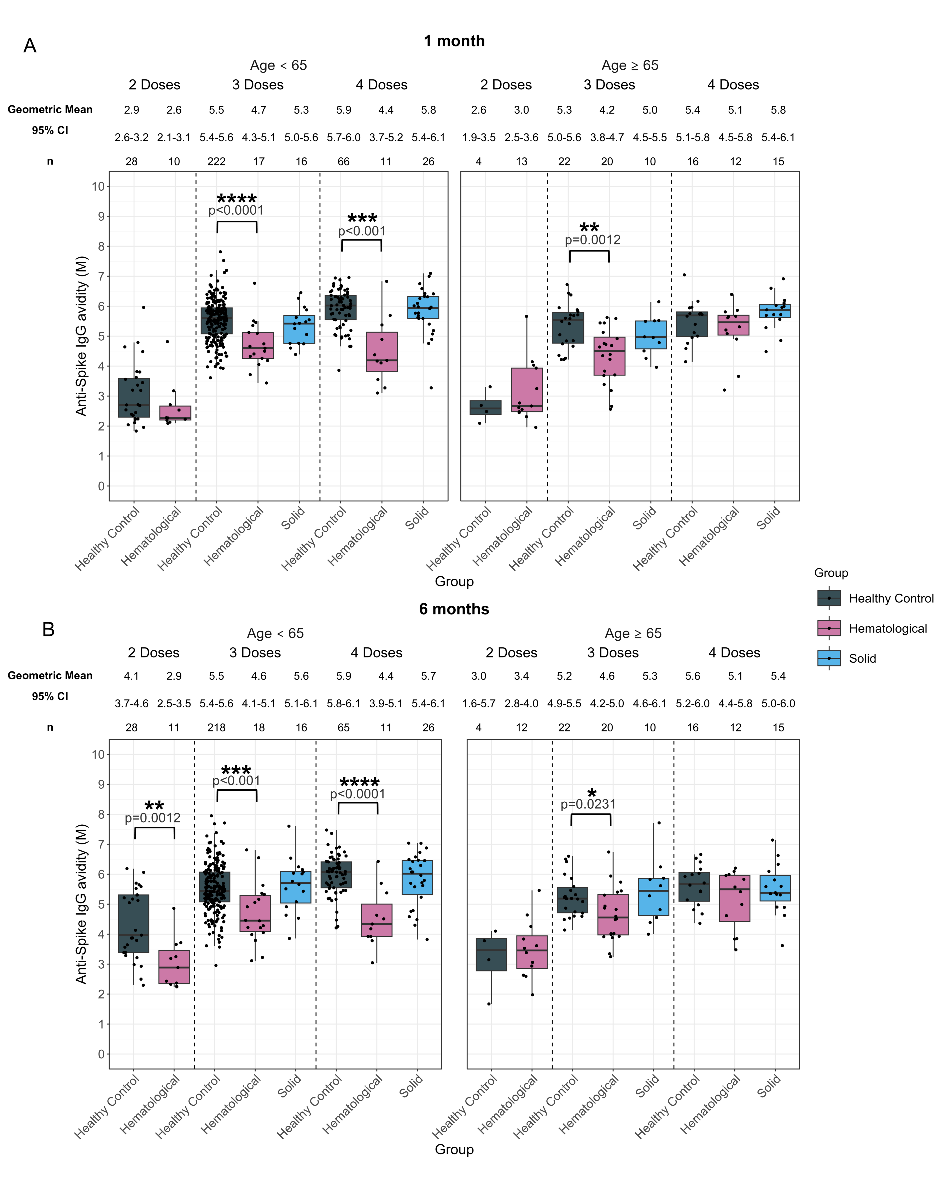


Supplementary Figure 7


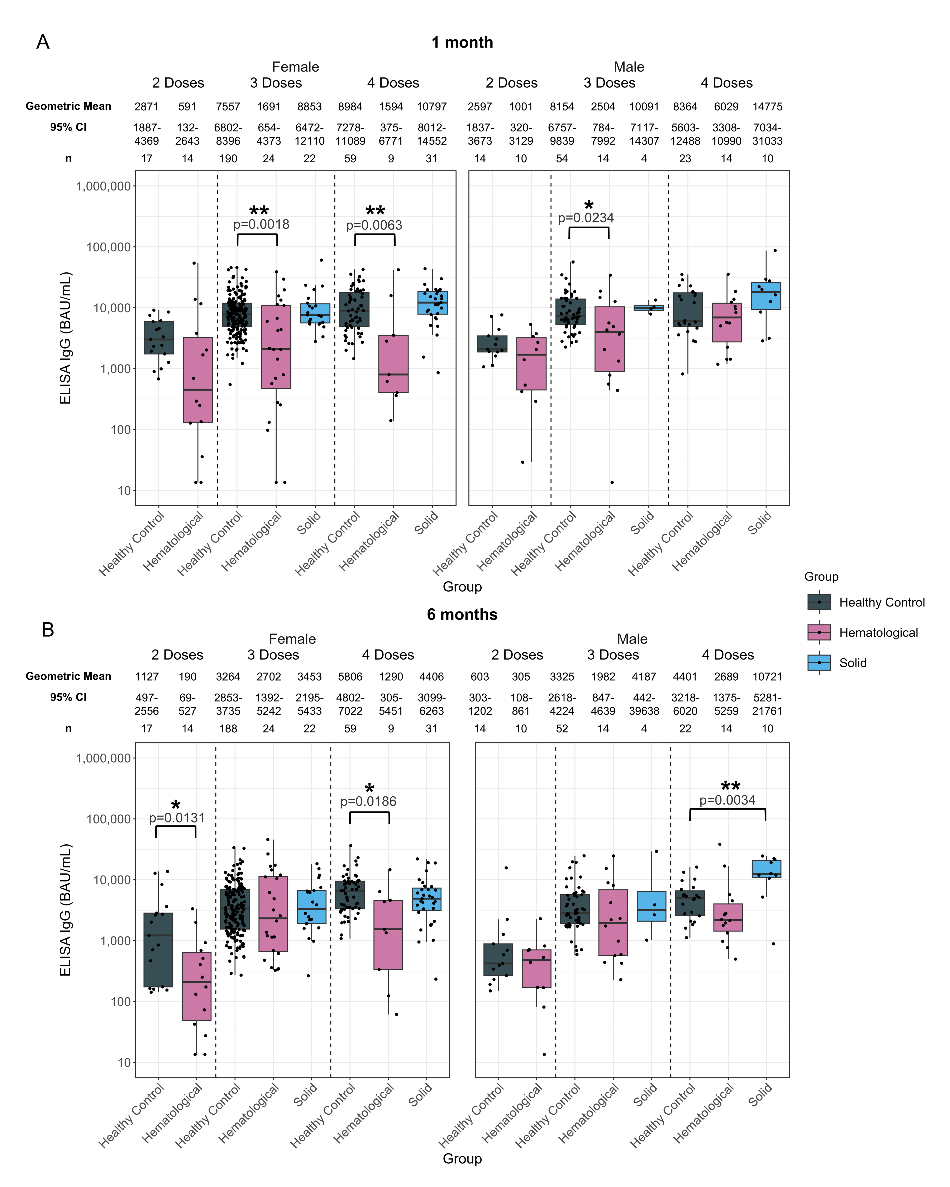


Supplementary Figure 8


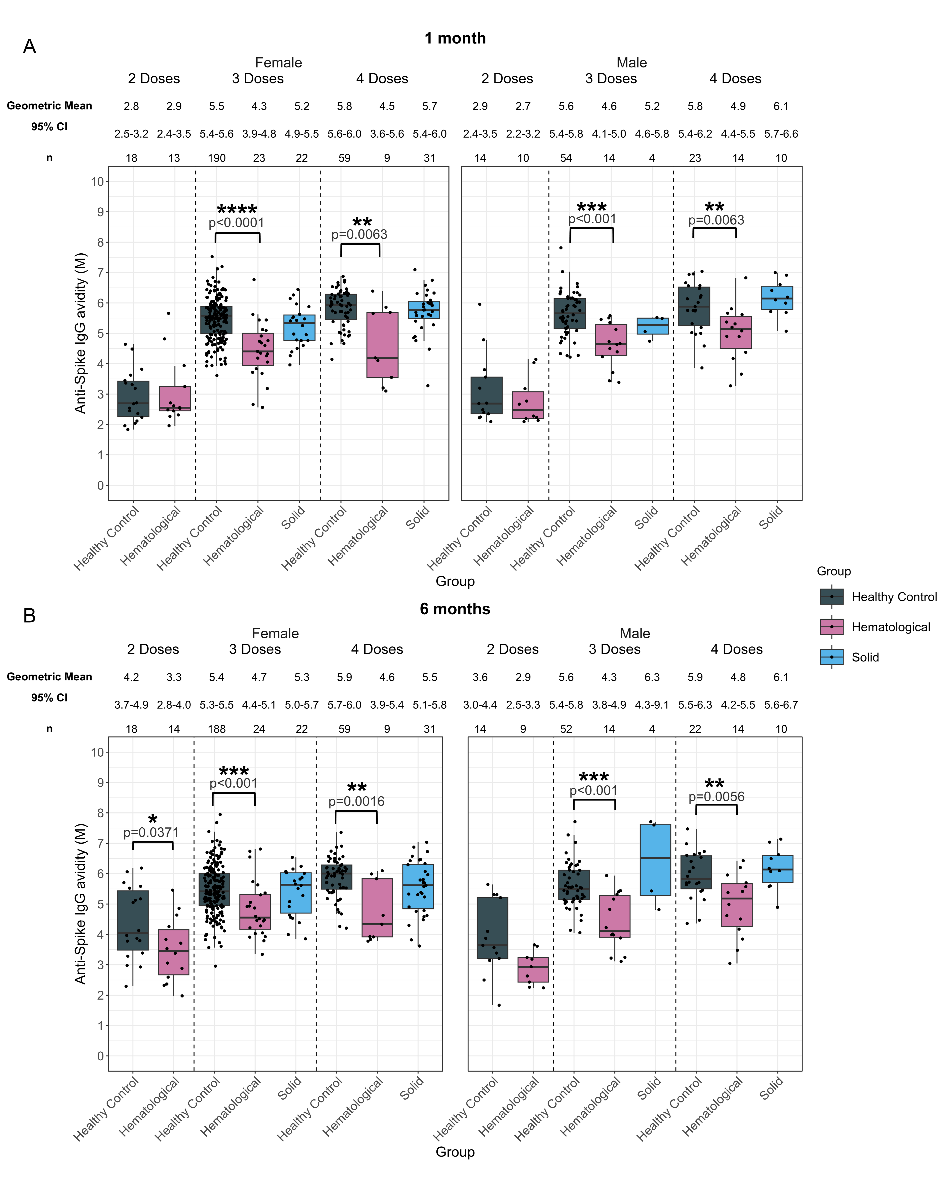


Supplementary Figure 9


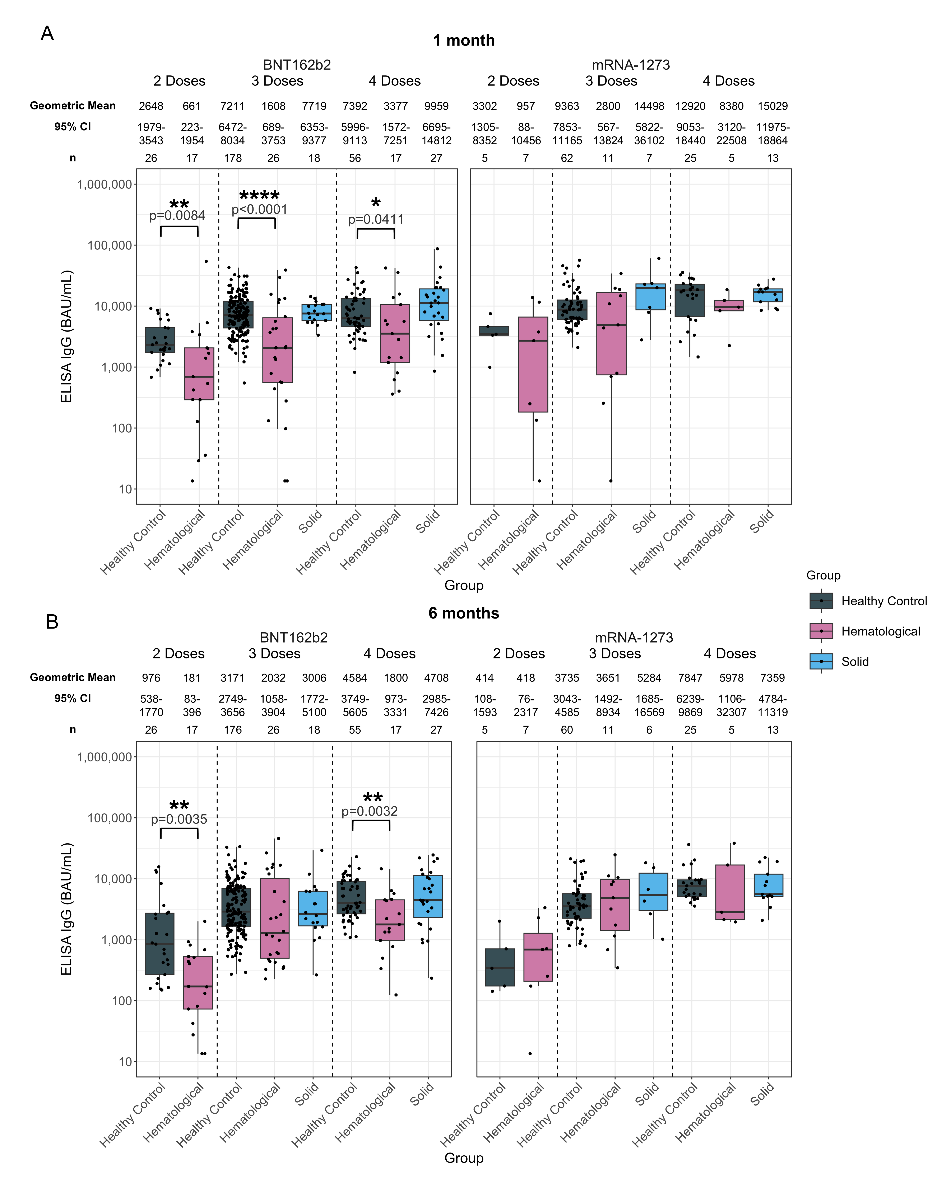


Supplementary Figure 10


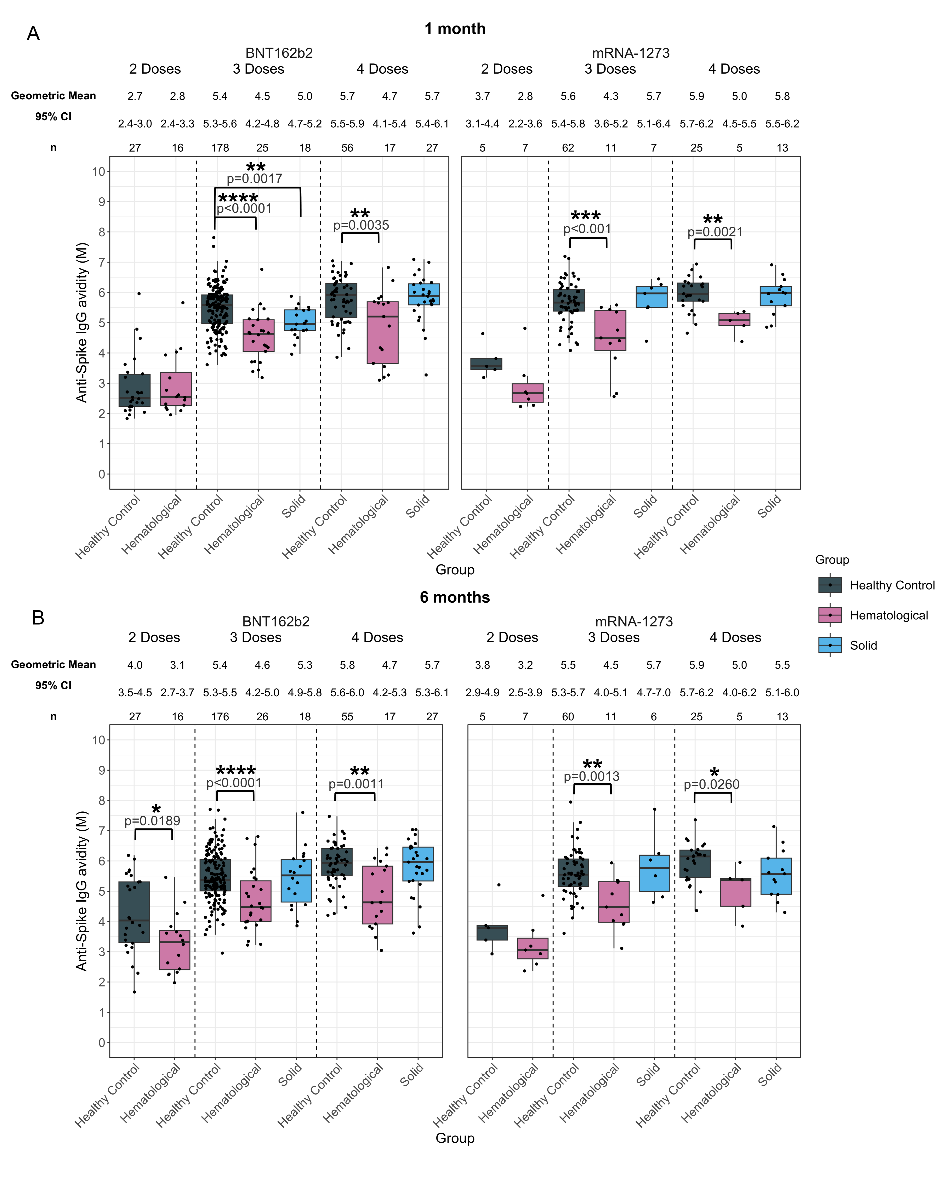


**3. Supplementary Figure Captions**

**Supplementary Figure 1. Anti-SARS-CoV-2 Spike IgG levels post vaccination in serum samples from cancer and healthy cohorts.** Comparison of serum anti-spike IgG levels in healthy, hematological cancer and solid cancer cohorts 1-month **(A)** and 6-month **(B)** post-vaccination. Anti-spike IgG levels were presented as Geometric Mean (GM) with 95% confidence intervals (95% CI). Box plots here and in subsequent figures show median (horizontal bar), the first and third quartiles. Differences assessed by non-parametric Wilcoxon rank sum test. *, p<0.05, **, p<0.01, ***, p<0.001, ****, p<0.0001.

**Supplementary Figure 2. Anti-SARS-CoV-2 Spike IgG avidity post vaccination in serum samples from cancer and healthy cohorts.** Comparison of serum anti-spike IgG avidity in healthy, hematological cancer and solid cancer cohorts 1-month **(A)** and 6-month **(B)** post-vaccination. Anti-spike IgG avidity was presented as Geometric Mean (GM) with 95% confidence intervals (95% CI). Differences assessed by non-parametric Wilcoxon rank sum test. *, p<0.05, **, p<0.01, ***, p<0.001, ****, p<0.0001.

**Supplementary Figure 3. Percent change in anti-spike IgG levels from 1 month to 6 months post vaccination in cancer and healthy cohorts.** Comparison of percent change in serum anti-spike IgG levels between 1 month and 6 months post-vaccination in individuals receiving 2 doses **(A)**, 3 doses **(B)** and 4 doses **(C)** of vaccination in healthy, hematological cancer and solid cancer cohorts. Percent change results were reported as Median with the first and third quartiles (Q1 and Q3). The p values indicate the significance levels of the percent change between 1 month and 6 months in the cancer cohort, compared to the percent change observed in the healthy cohort with the corresponding doses. Solid red line represents the connecting line of geometric mean titer at 1 month and 6 months in each cohort. Differences assessed by non-parametric Wilcoxon rank sum test.

**Supplementary Figure 4. Percent change in anti-spike IgG avidity from 1 month to 6 months post vaccination in cancer and healthy cohorts.** Comparison of percent change in serum anti-spike IgG avidity between 1 month and 6 months post-vaccination in individuals receiving 2 doses **(A)**, 3 doses **(B)** and 4 doses **(C)** of vaccination in healthy, hematological cancer and solid cancer cohorts. Percent change results were reported as Median with the first and third quartiles (Q1 and Q3). The p values indicate the significance levels of the percent change between 1 month and 6 months in the cancer cohort, compared to the percent change observed in the healthy cohort with the corresponding doses. Solid red line represents the connecting line of geometric mean titer at 1 month and 6 months in each cohort. Differences assessed by non-parametric Wilcoxon rank sum test.

**Supplementary Figure 5. Anti-SARS-CoV-2 Spike IgG levels post vaccination in serum samples from cancer and healthy cohorts within different age groups**. - <65 years old and ≥ 65 years old. Comparison of serum anti-spike IgG levels in healthy, hematological cancer and solid cancer cohorts within different age groups 1-month **(A)** and 6-month **(B)** post-vaccination. Anti-spike IgG levels were presented as Geometric Mean (GM) with 95% confidence intervals (95% CI). Differences assessed by non-parametric Wilcoxon rank sum test. *, p<0.05, **, p<0.01, ***, p<0.001, ****, p<0.0001.

**Supplementary Figure 6. Anti-SARS-CoV-2 Spike IgG avidity post vaccination in serum samples from cancer and healthy cohorts within different age groups**. - <65 years old and ≥ 65 years old. Comparison of serum anti-spike IgG avidity in healthy, hematological cancer and solid cancer cohorts within different age groups 1-month **(A)** and 6-month **(B)** post-vaccination. Anti-spike IgG avidity was presented as Geometric Mean (GM) with 95% confidence intervals (95% CI). Differences assessed by non-parametric Wilcoxon rank sum test. *, p<0.05, **, p<0.01, ***, p<0.001, ****, p<0.0001.

**Supplementary Figure 7. Anti-SARS-CoV-2 Spike IgG levels post vaccination in serum samples from cancer and healthy cohorts within different sex groups.** Comparison of serum anti-spike IgG levels in healthy, hematological cancer and solid cancer cohorts within different sex groups 1-month **(A)** and 6-month **(B)** post-vaccination. Anti-spike IgG levels were presented as Geometric Mean (GM) with 95% confidence intervals (95% CI). Differences assessed by non-parametric Wilcoxon rank sum test. *, p<0.05, **, p<0.01, ***, p<0.001, ****, p<0.0001.

**Supplementary Figure 8. Anti-SARS-CoV-2 Spike IgG avidity post vaccination in serum samples from cancer and healthy cohorts within different sex groups.** Comparison of serum anti-spike IgG avidity in healthy, hematological cancer and solid cancer cohorts within different sex groups 1-month **(A)** and 6-month **(B)** post-vaccination. Anti-spike IgG avidity was presented as Geometric Mean (GM) with 95% confidence intervals (95% CI. Differences assessed by non-parametric Wilcoxon rank sum test. *, p<0.05, **, p<0.01, ***, p<0.001, ****, p<0.0001.

**Supplementary Figure 9. Anti-SARS-CoV-2 Spike IgG levels post vaccination in serum samples from cancer and healthy cohorts within different vaccine groups.** Comparison of serum anti-spike IgG levels in healthy, hematological cancer and solid cancer cohorts within different vaccine groups 1-month **(A)** and 6-month **(B)** post-vaccination. Anti-spike IgG levels were presented as Geometric Mean with 95% confidence intervals (95% CI). Differences assessed by non-parametric Wilcoxon rank sum test. *, p<0.05, **, p<0.01, ***, p<0.001, ****, p<0.0001.

**Supplementary Figure 10. Anti-SARS-CoV-2 Spike IgG avidity post vaccination in serum samples from cancer and healthy cohorts within different vaccine groups.** Comparison of serum anti-spike IgG avidity in healthy, hematological cancer and solid cancer cohorts within different vaccine groups 1-month **(A)** and 6-month **(B)** post-vaccination. Anti-spike IgG levels were presented as Geometric Mean with 95% confidence intervals (95% CI). Differences assessed by non-parametric Wilcoxon rank sum test. *, p<0.05, **, p<0.01, ***, p<0.001, ****, p<0.0001.
