# Supplementary figures and images for: Circulating SARS-CoV-2 spike IgG antibody responses in cancer patients following multiple COVID-19 vaccination boosters
Source: Front Immunol. 2025 Aug 12;16:1629473. doi: 10.3389/fimmu.2025.1629473 (PMC12378708; doi:10.3389/fimmu.2025.1629473)

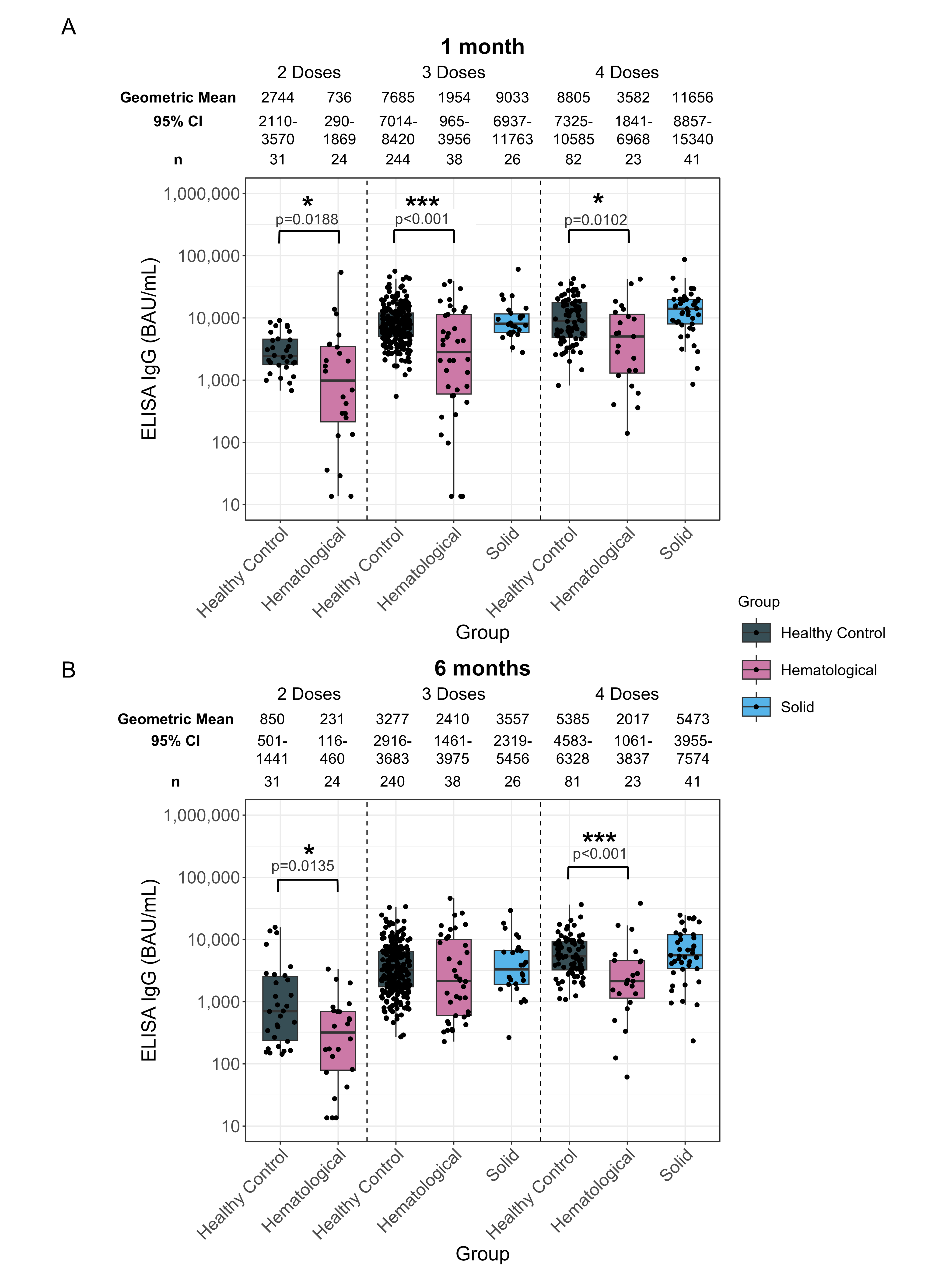

Supplement: Supplementary file 2 [file Image1.tif]

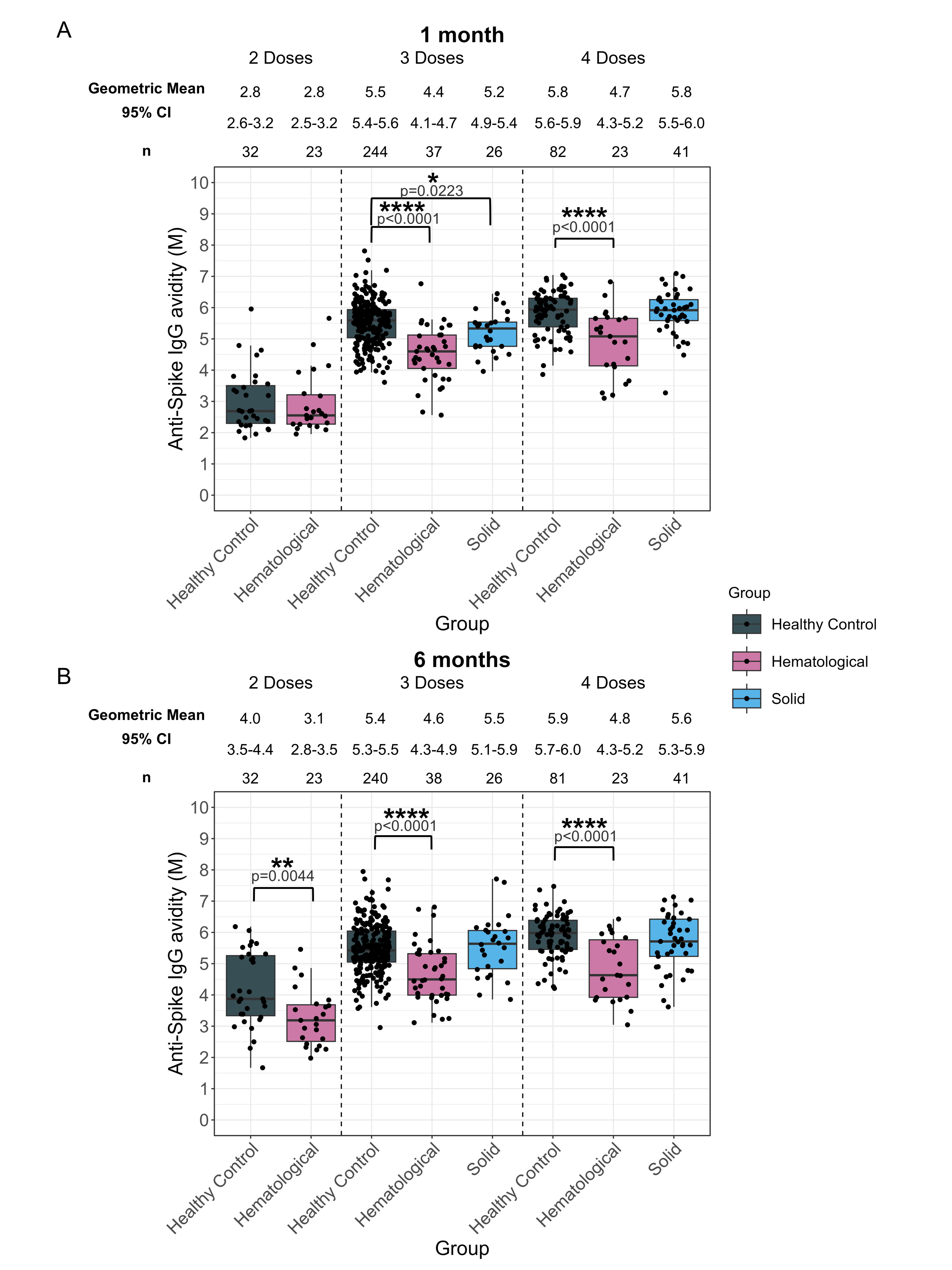

Supplement: Supplementary file 3 [file Image2.tif]

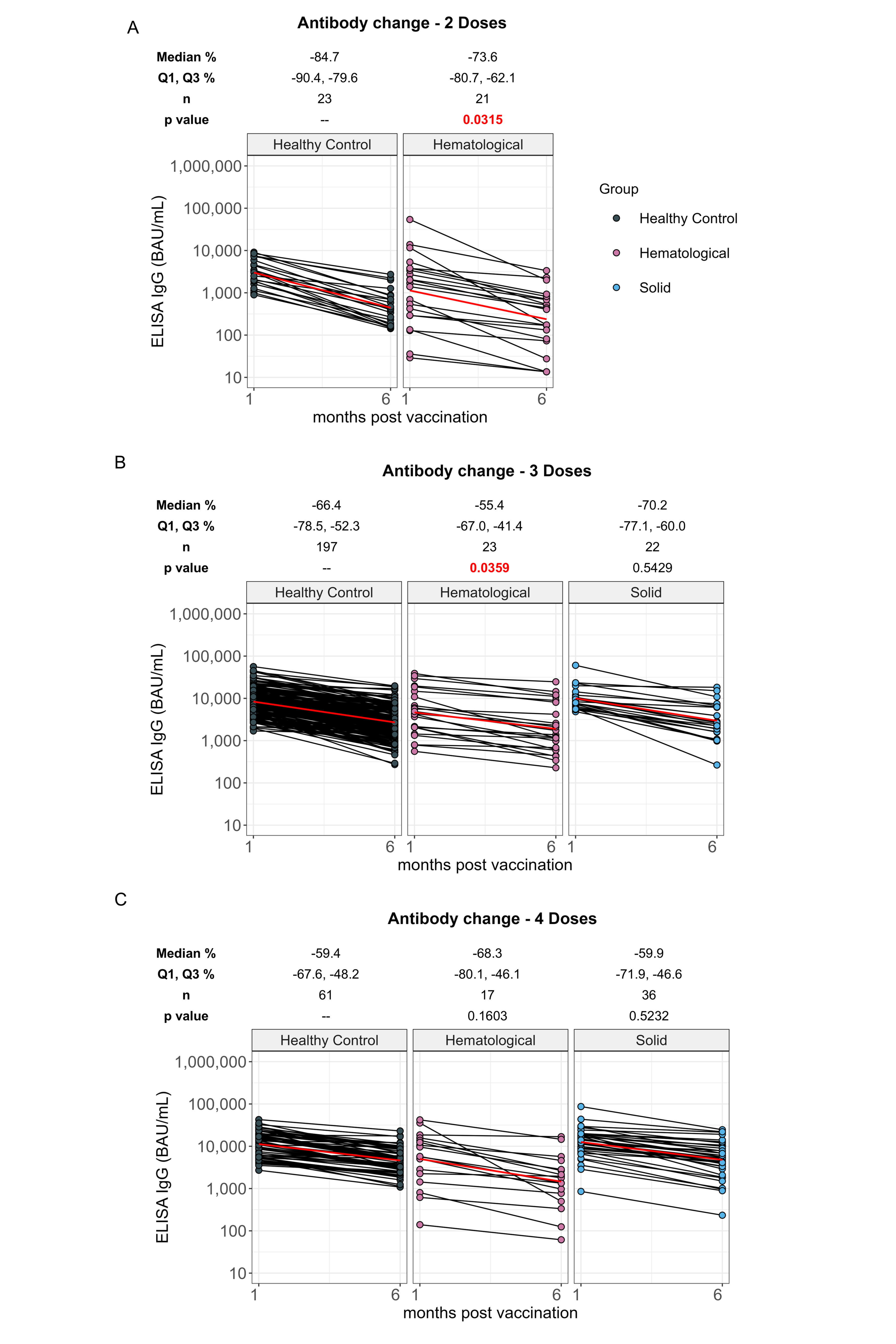

Supplement: Supplementary file 4 [file Image3.tif]

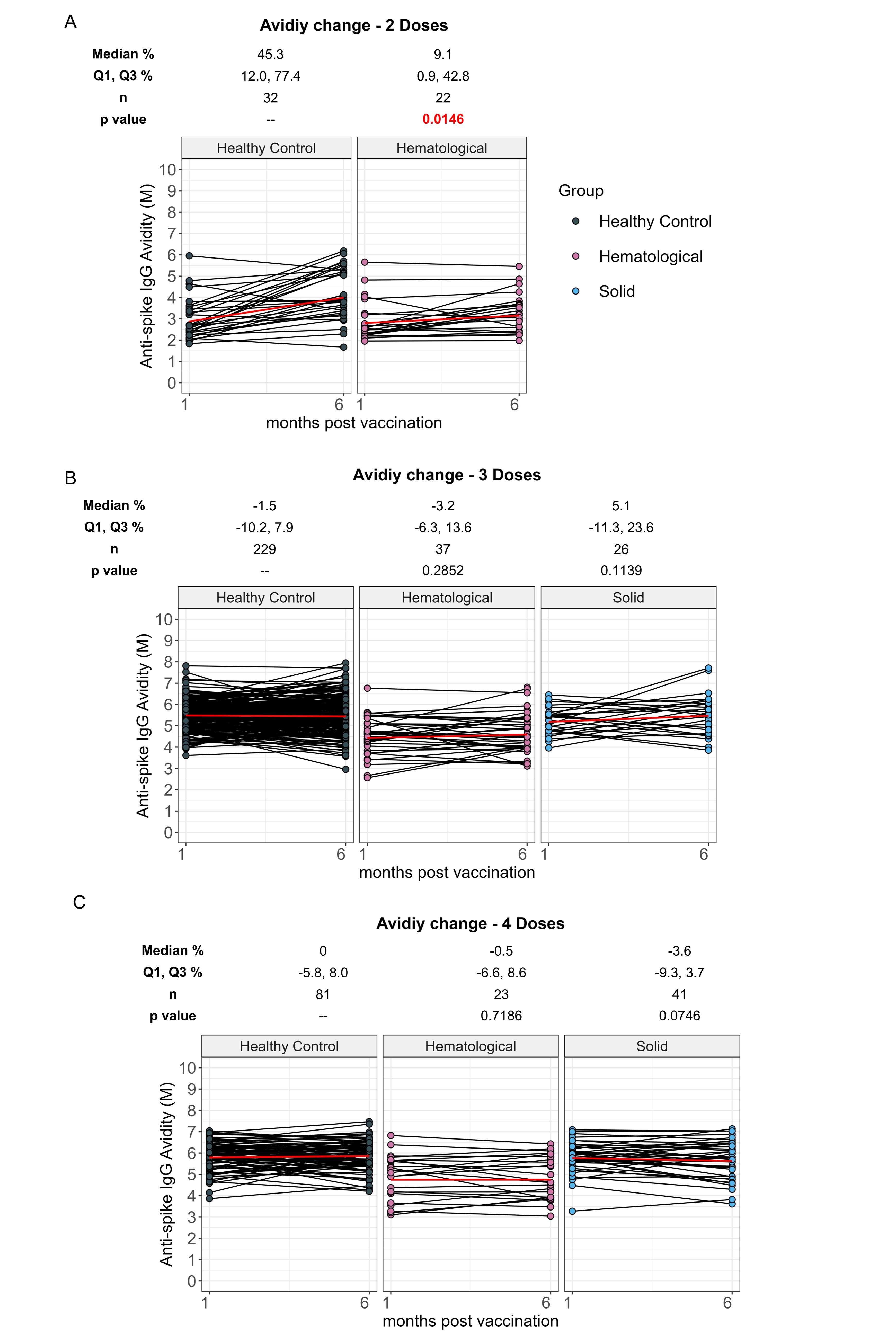

Supplement: Supplementary file 5 [file Image4.tif]

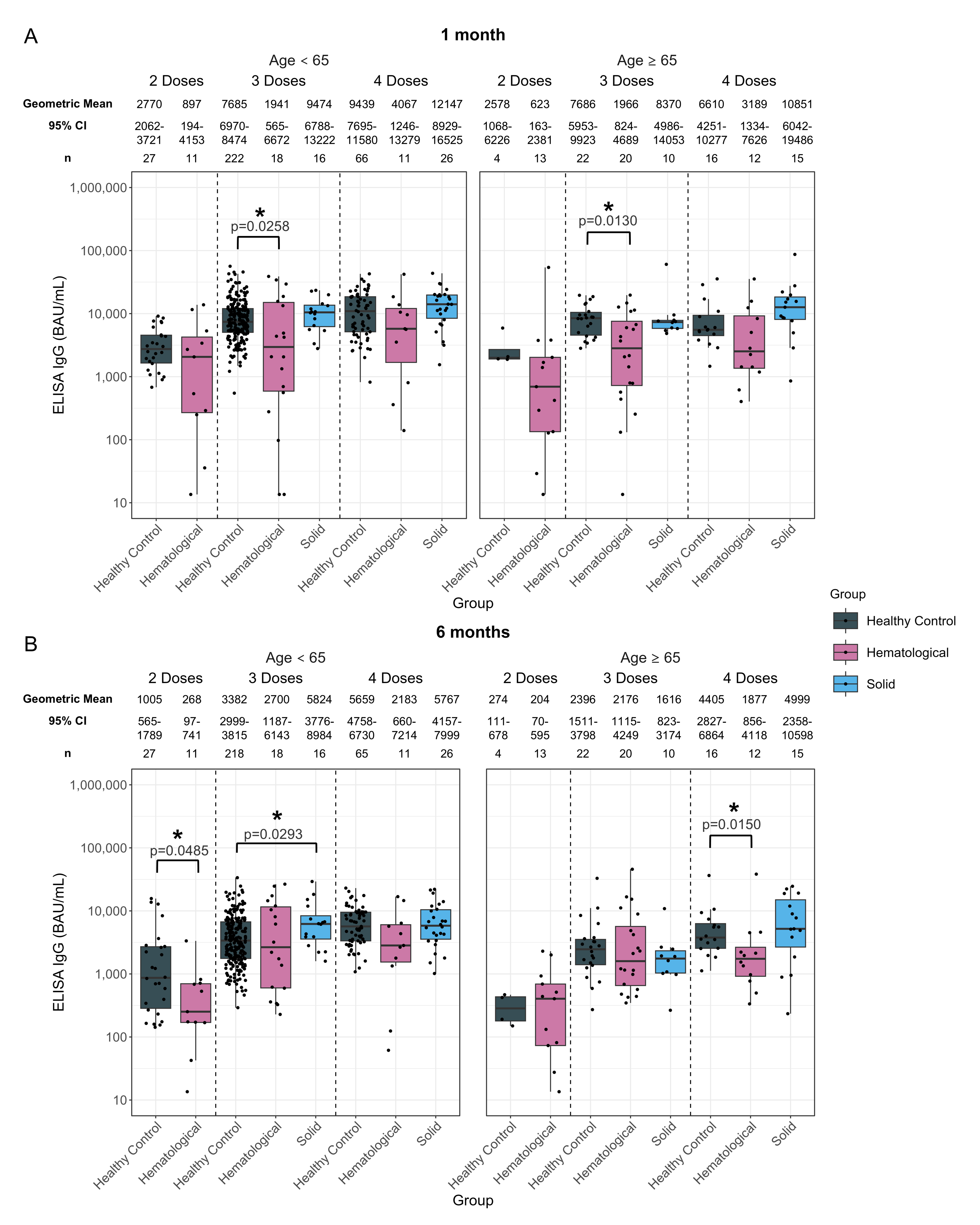

Supplement: Supplementary file 6 [file Image5.tif]

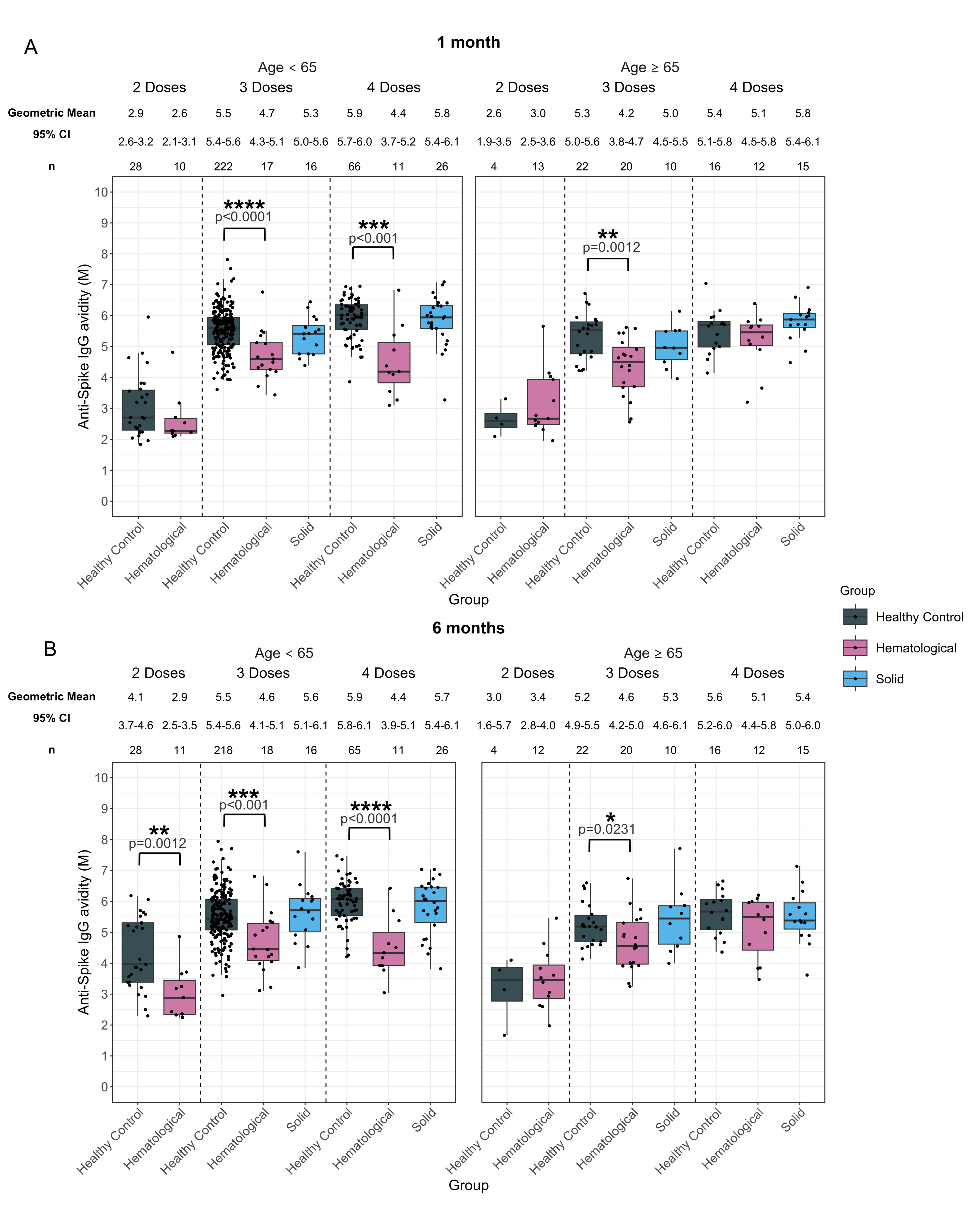

Supplement: Supplementary file 7 [file Image6.tif]

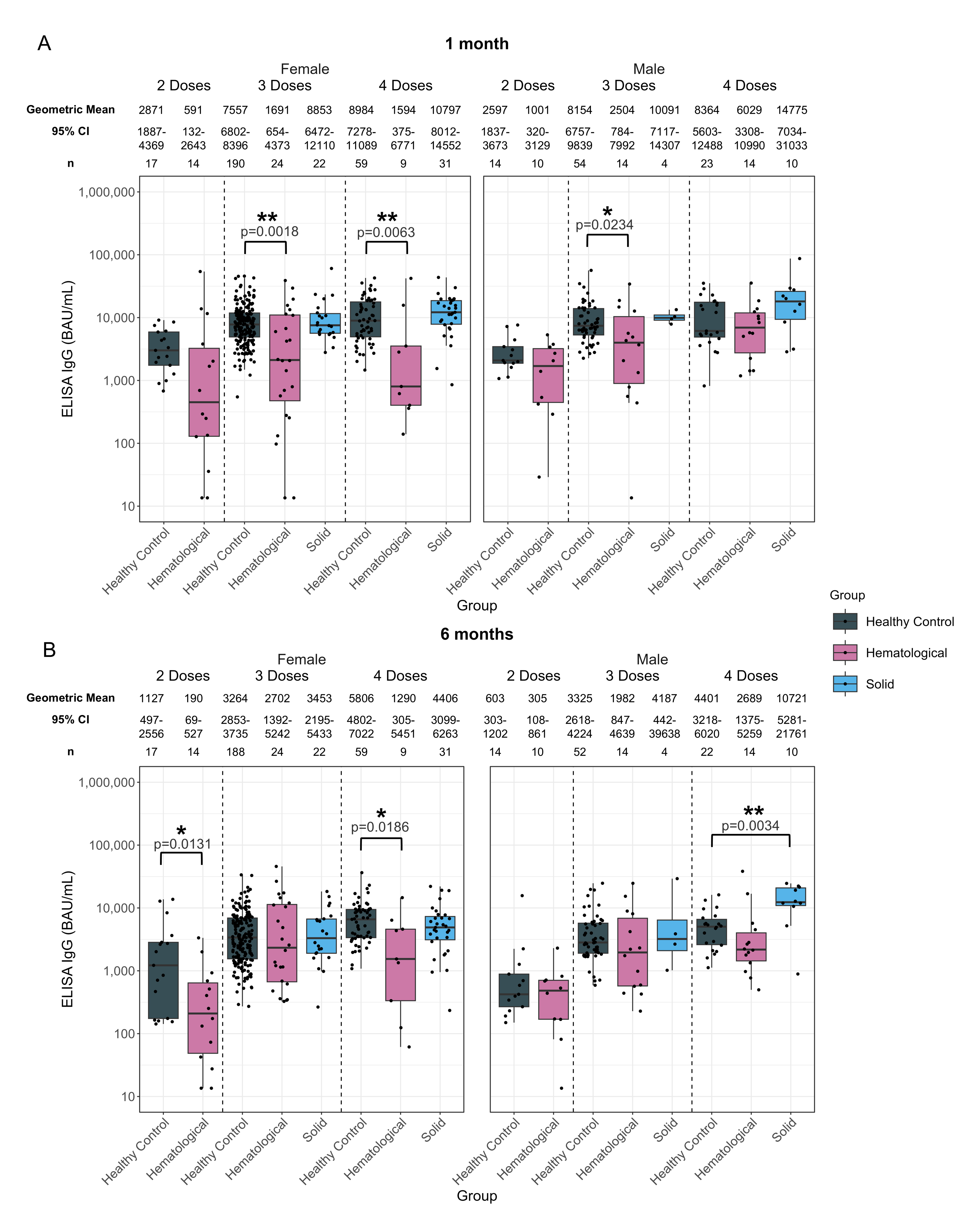

Supplement: Supplementary file 8 [file Image7.tif]

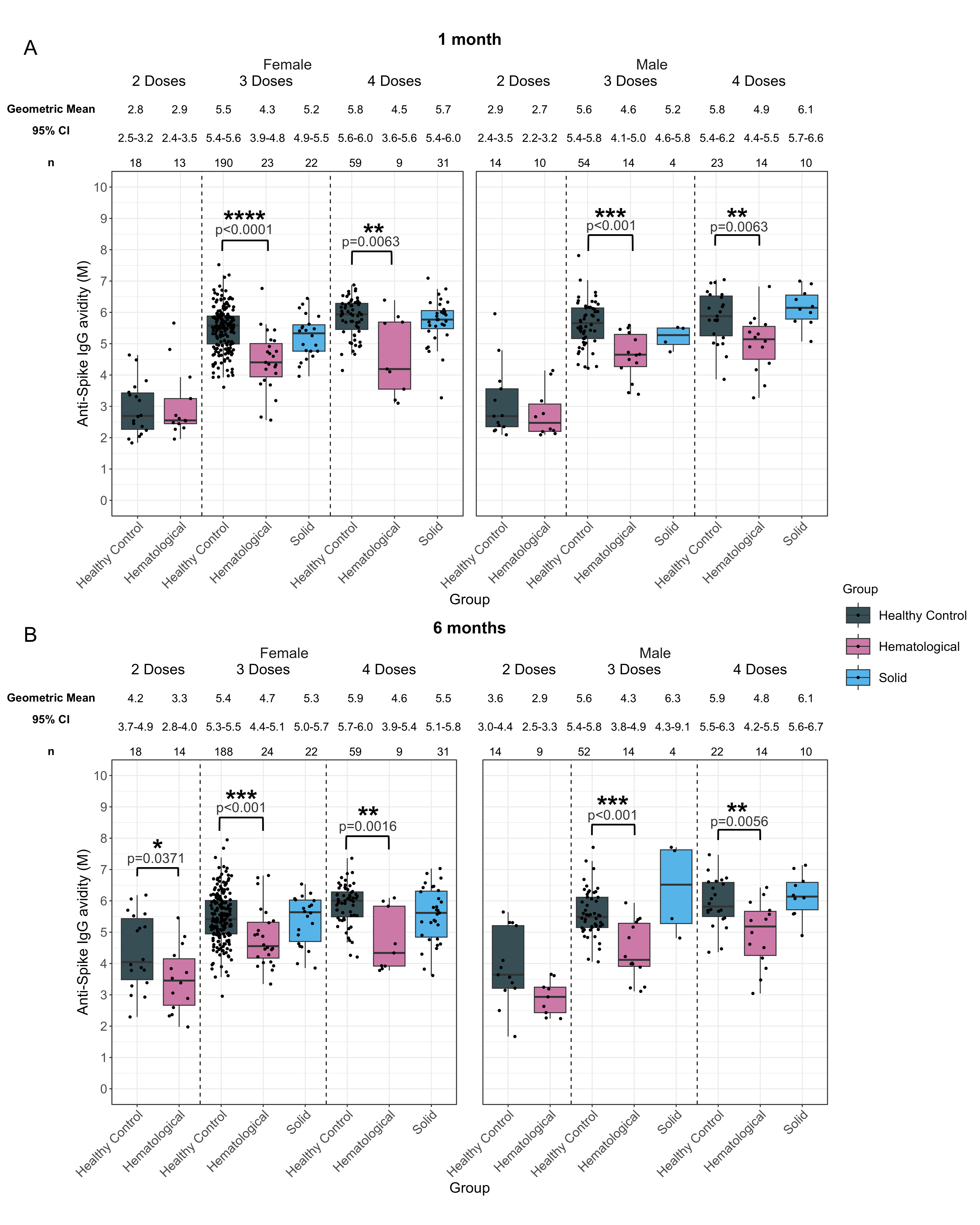

Supplement: Supplementary file 9 [file Image8.tif]

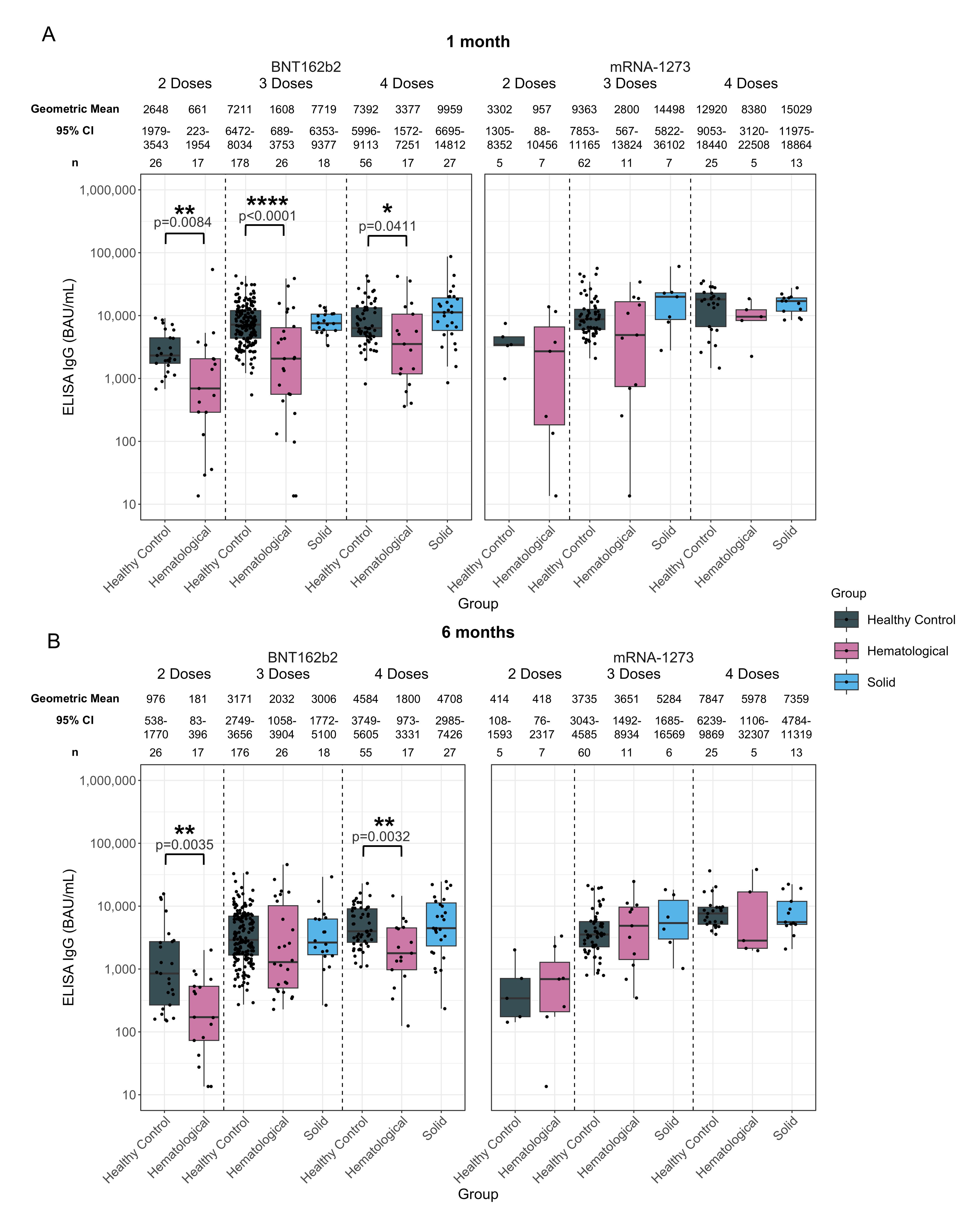

Supplement: Supplementary file 10 [file Image9.tif]

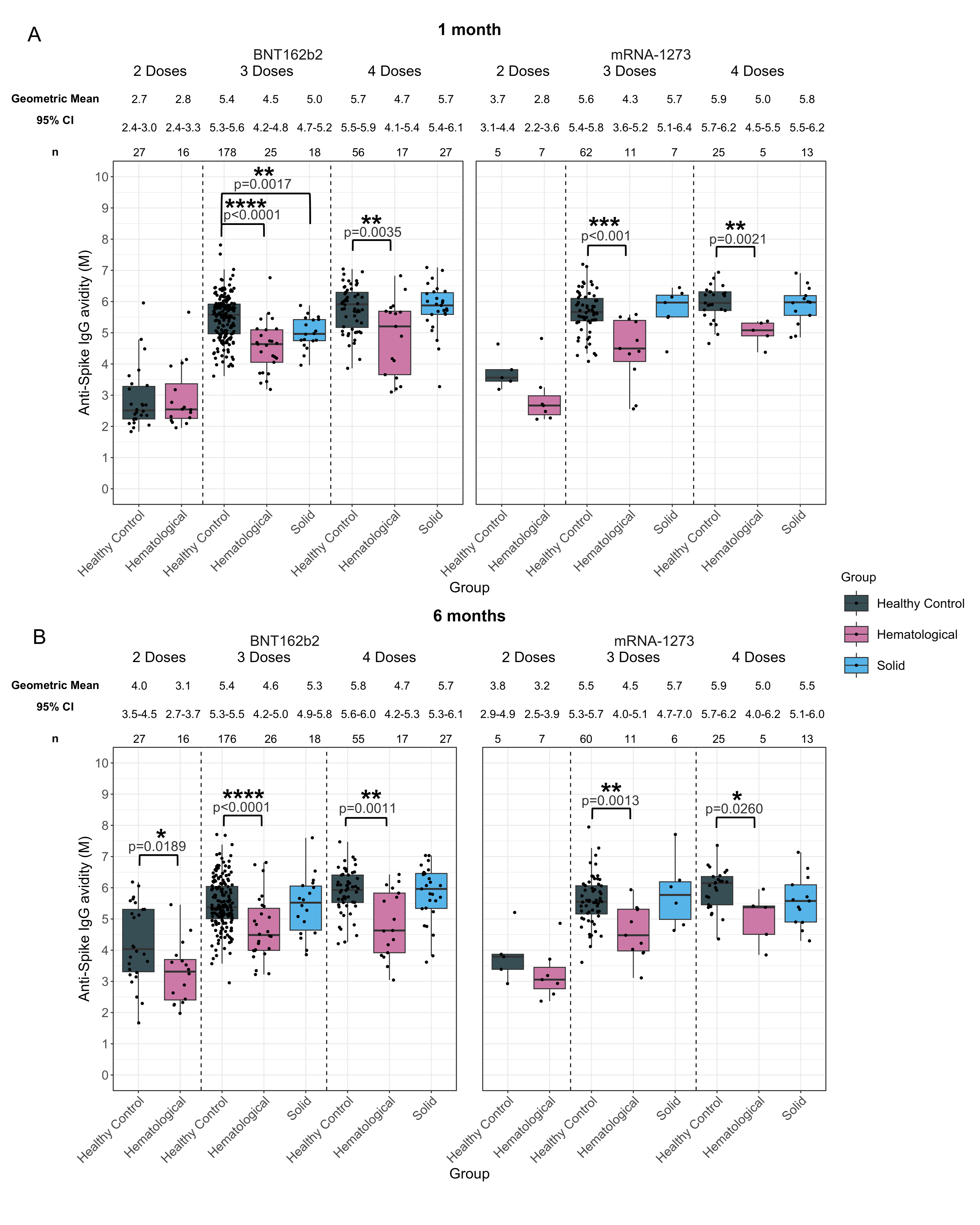

Supplement: Supplementary file 11 [file Image10.tif]
